# A Ceratopsian Dinosaur from the Lower Cretaceous of Western North America, and the Biogeography of Neoceratopsia

**DOI:** 10.1371/journal.pone.0112055

**Published:** 2014-12-10

**Authors:** Andrew A. Farke, W. Desmond Maxwell, Richard L. Cifelli, Mathew J. Wedel

**Affiliations:** 1 Raymond M. Alf Museum of Paleontology, Claremont, California, United States of America; 2 Department of Biological Sciences, University of the Pacific, Stockton, California, United States of America; 3 Sam Noble Oklahoma Museum of Natural History, Norman, Oklahoma, United States of America; 4 College of Podiatric Medicine, Western University of Health Sciences, Pomona, California, United States of America; Penn State University, United States of America

## Abstract

The fossil record for neoceratopsian (horned) dinosaurs in the Lower Cretaceous of North America primarily comprises isolated teeth and postcrania of limited taxonomic resolution, hampering previous efforts to reconstruct the early evolution of this group in North America. An associated cranium and lower jaw from the Cloverly Formation (?middle–late Albian, between 104 and 109 million years old) of southern Montana is designated as the holotype for *Aquilops americanus* gen. et sp. nov. *Aquilops americanus* is distinguished by several autapomorphies, including a strongly hooked rostral bone with a midline boss and an elongate and sharply pointed antorbital fossa. The skull in the only known specimen is comparatively small, measuring 84 mm between the tips of the rostral and jugal. The taxon is interpreted as a basal neoceratopsian closely related to Early Cretaceous Asian taxa, such as *Liaoceratops* and *Auroraceratops*. Biogeographically, *A. americanus* probably originated via a dispersal from Asia into North America; the exact route of this dispersal is ambiguous, although a Beringian rather than European route seems more likely in light of the absence of ceratopsians in the Early Cretaceous of Europe. Other amniote clades show similar biogeographic patterns, supporting an intercontinental migratory event between Asia and North America during the late Early Cretaceous. The temporal and geographic distribution of Upper Cretaceous neoceratopsians (leptoceratopsids and ceratopsoids) suggests at least intermittent connections between North America and Asia through the early Late Cretaceous, likely followed by an interval of isolation and finally reconnection during the latest Cretaceous.

## Introduction

**Figure 1:**
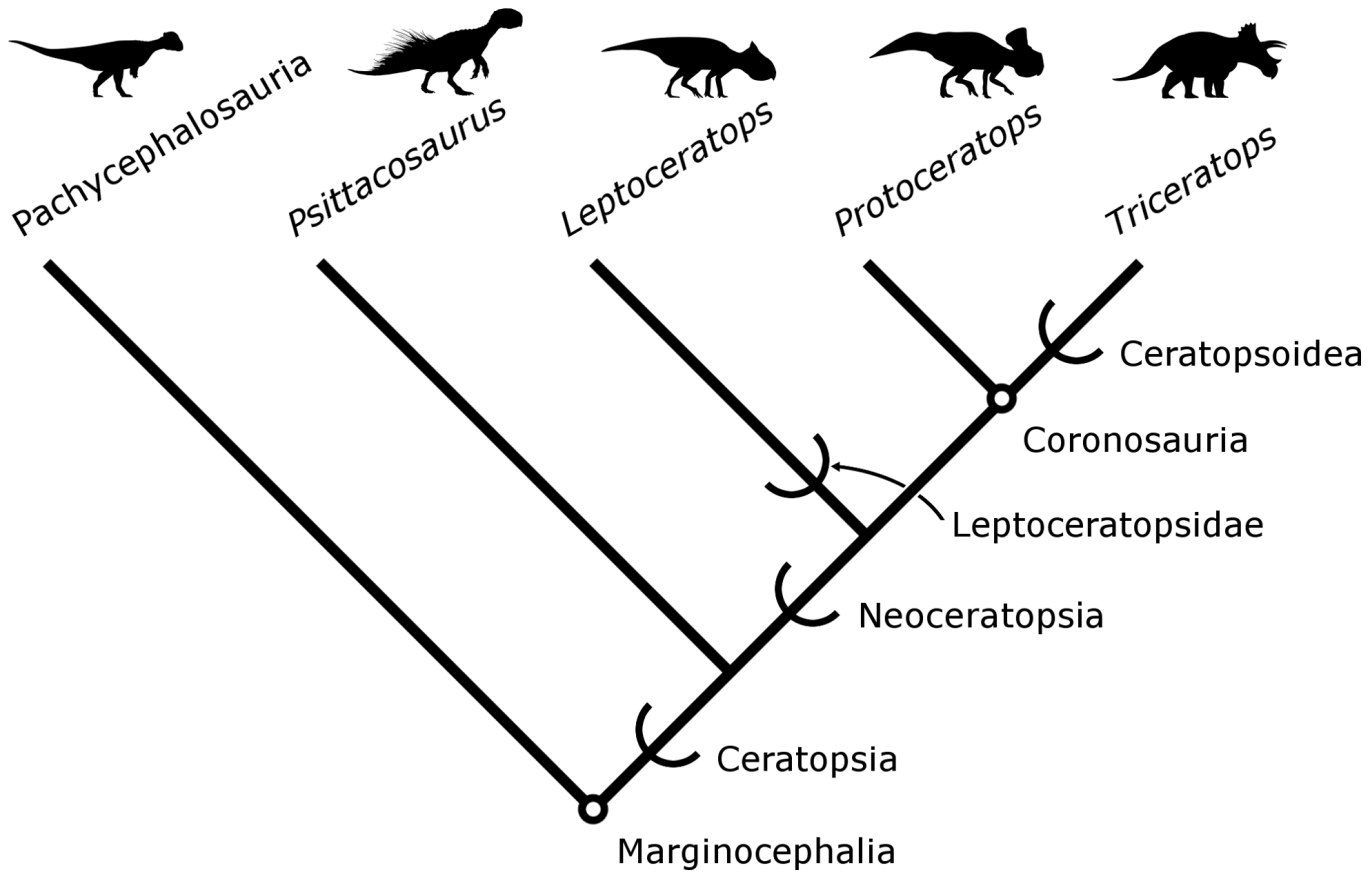
Summary phylogeny of Ceratopsia with nomenclatural conventions used in this paper. Marginocephalia and Coronosauria are node-based clades, indicated by circles. The rest of the clades shown here are stem-based, indicated by half-brackets. See text for definitions and taxonomic authorities. Silhouettes are not to scale (*Acrotholus* by G. Monger, *Psittacosaurus* by J. Headden, *Triceratops* by R. Amos; all others by A. Farke; all images are CC-BY and provided via www.phylopic.org).

**Figure 2:**
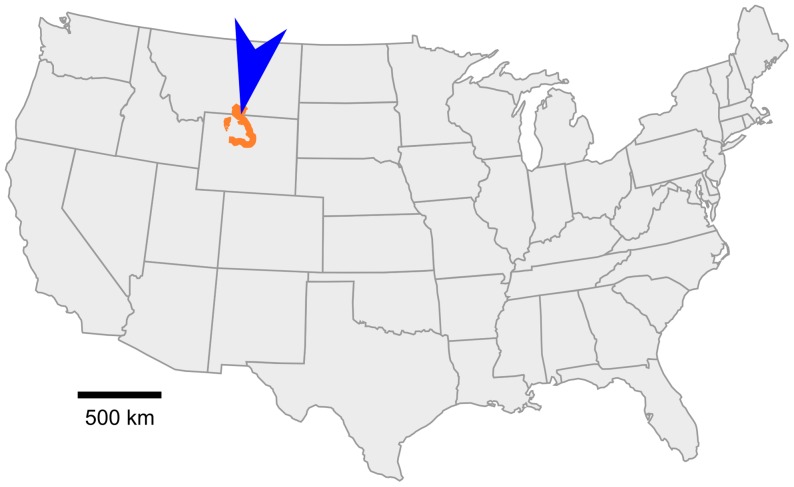
Location map for the holotype of *Aquilops americanus* within the contiguous United States of America. Outcrops of Cloverly Formation within the Bighorn Basin of Montana and Wyoming are shown in orange; the actual width of the outcrop band has been exaggerated for visual clarity. The approximate location of OMNH locality V1057, within Carbon County, Montana, is indicated by the blue arrow.

**Figure 3:**
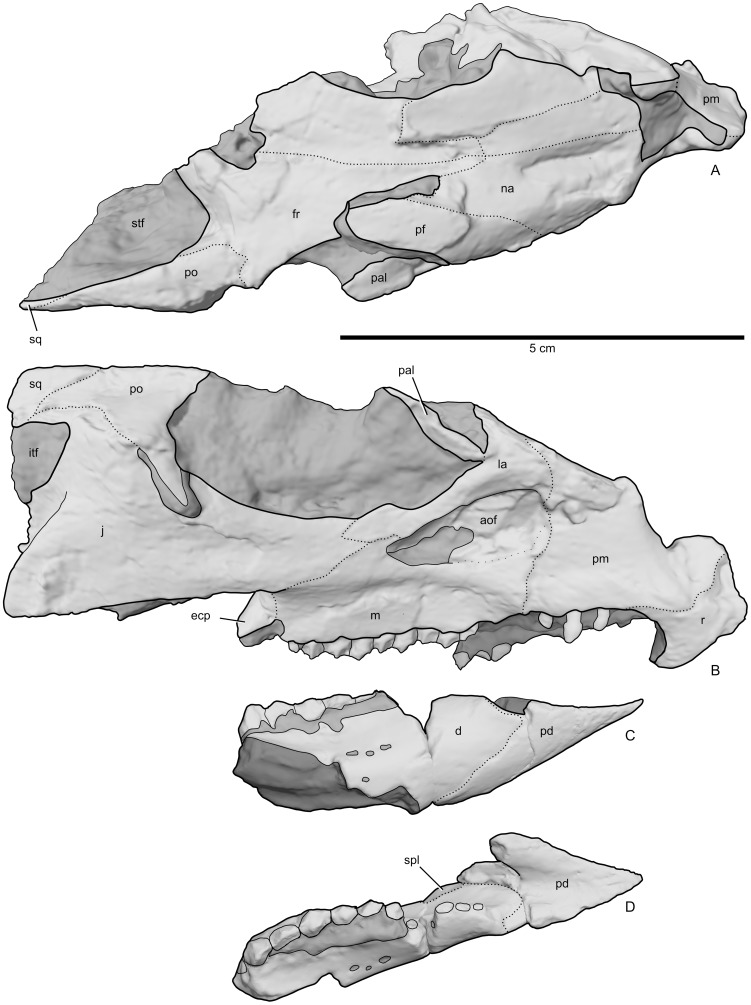
Skull of *Aquilops americanus*, OMNH 34557 (holotype). Partial cranium in A) dorsal and B) right lateral views. Partial lower jaw in C) right lateral and D) dorsal views. This interpretive figure is based on surface scans of the original specimen, with sutures highlighted. The lower jaw is reversed, to facilitate placement with the skull. Abbreviations: aof, antorbital fossa; d, dentary; ecp, ectopterygoid; fr, frontal; itf, infratemporal fenestra; j, jugal; la, lacrimal; m, maxilla; na, nasal; pal, palpebral; pd, predentary; pf, prefrontal; pm, premaxilla; po, postorbital; r, rostral; spl, sutural surface for splenial; sq, squamosal; stf, supratemporal fenestra.

Neoceratopsia constitute one of the most taxonomically diverse and morphologically disparate clades of ornithischian dinosaurs during the Cretaceous, and are particularly known for their highly derived cranial anatomy (Figure 1; [1]–[3]). Early representatives, such as *Liaoceratops yanzigouensis* Xu et al. 2002 [1], exhibit expanded bony platforms for the jaw muscles that are elaborated into a broad frill in later taxa (e.g., *Protoceratops andrewsi* Granger and Gregory 1923 [4]). Ceratopsids, including *Triceratops* Marsh 1891 [5], further augmented the skull with elongate horns and spikes [2].

A series of well-preserved specimens from a variety of taxa has firmly placed the origin and early diversification of Neoceratopsia in the Early Cretaceous of Asia [1], [6]–[8]. However, the timing and phylogenetic affinities of the clade's first dispersal into North America have remained unclear, due to a dearth of fossilized material that, until now, has consisted of relatively uninformative isolated teeth and postcranial elements [9]–[12]. These specimens indicate the presence of Neoceratopsia in the Early Cretaceous of North America, but cannot be further identified. The arrival of neoceratopsians on the continent is important for understanding the nature and timing of broader faunal interchanges during the Cretaceous. Specimens representing other dinosaur clades that potentially dispersed between Asia and North America during the Early Cretaceous—including tyrannosauroids [13], oviraptorosaurs [14], ornithomimids [15], therizinosaurs [16], and shamosaurine ankylosaurs [17]—are similarly fragmentary in most cases and have contributed to uncertainty regarding the mode and timing of faunal exchange. Additionally, there is uncertainty on whether the dispersal was directly between North America and Asia (trans-Beringia) or via Europe [12], [13], [18], although the absence of important Asian clades in Europe somewhat favors a trans-Beringian dispersal hypothesis [13], [18].

A skull from the Albian part of the Cloverly Formation of Montana (Figures 2, 3) represents the first neoceratopsian from the North American Early Cretaceous that is diagnostic to the species-level. This new taxon, *Aquilops americanus* gen et sp. nov., exhibits definitive neoceratopsian features and is closely related to coeval Asian species. Furthermore, the discovery of *Aquilops* adds unambiguous support for a late Early Cretaceous (∼113–105 Ma) intercontinental migratory event between Asia and North America [13], [19], as well as support for a complex set of migratory events for organisms between North America and Asia later in the Cretaceous.

### Geologic setting

The specimen described herein, OMNH 34557, was collected in the Cloverly Formation of Carbon County, Montana (Figure 2). Previous workers have designated subdivisions of the Cloverly Formation either numerically (from lowest/oldest to highest/youngest, Units IV–VII; [20]), alphabetically (from lowest, intervals A–C [21]), or as named members (from lowest, the Pryor Conglomerate, Little Sheep Mudstone, and Himes members [22]). OMNH 34557 originated from a deep red sandy claystone in the basal part of unit VII of the Cloverly Formation, as defined by Ostrom [20], corresponding to the Himes Member of Moberly [22] and within the C interval of Meyers et al. ([21]; redefined by [23]). Summarizing the partly conflicting evidence at hand (below), OMNH 34557 is likely Albian in age; we provisionally regard it as about 104–109 Ma old (middle to early late Albian).

The age of the Cloverly Formation is not well constrained; indeed, as whole, it is best regarded as simply Lower Cretaceous. There is general agreement that its basal part (Pryor Conglomerate Member of Moberly [22]; Unit IV of Ostrom [20]; A interval of Meyers et al. [21]) is pre-Aptian (we avoid usage of the term “Neocomian” following Sames et al. [24]) in age, and that it may correlate with the lower Lakota (L1 interval or Chilson Member) Formation, Wyoming and South Dakota, and the lower Cedar Mountain (Yellow Cat Member) Formation, Utah [23], both of which may be as old as Berriasian–Valanginian [24]–[27], though radiometric dates suggest a younger (Barremian–early Aptian) age for the Yellow Cat Member (see Ludvigson et al. [28], and references therein).

Upper age constraints for the Cloverly Formation are also open to debate. The upper parts of the Cloverly (units V–VII of Ostrom [20]; Little Sheep Mudstone and Himes members of Moberly [22]; intervals B–C of Meyers et al. [21]) have long been regarded as being Aptian and/or Albian in age [20], with recent authors favoring the younger part of this range (e.g., [29]; but also see [30]). Stratigraphically, the Cloverly Formation is overlain by the Sykes Mountain Formation, a nearshore marine unit that lacks age-diagnostic fossils [22]. The Sykes Mountain Formation grades upward into the Thermopolis Shale, which contains the widespread bivalve *Inoceramus comancheanus* Cragin 1894 [31] and is considered to date to the middle part of the Late Albian [32]. This implies an approximate upper age constraint of between 103–105 Ma for the Cloverly Formation. Zaleha ([23], p. 892) reviewed then-available (2006) geochronologic and biochronologic evidence, concluding (largely based on palynomorphs) that Cloverly B–C intervals are “Albian, but no younger than middle Albian” (i.e., about 108–113 Ma). Since then, radiometric dates have appeared in two abstracts but have not been formally published. Burton et al. ([33], p. 52) reported a radiometric determination of 108.5±0.2 Ma (i.e., middle Albian) “from Beaver Creek near Shell, Wyoming at about 75 m above the contact with the underlying Morrison Formation” (hence presumably within units V–VII of Ostrom [20]). D'Emic and Britt ([34]; see also [35]) presented a younger date of 103.49±0.79–1.17 Ma (late Albian) based on detrital zircons from unit VI or basal unit VII.

## Materials and Methods

### Institutional abbreviations

AMNH, American Museum of Natural History, New York, New York, United States of America; CAGS-IG, Chinese Academy of Geological Sciences-Institute of Geology, Beijing, China; CMN, Canadian Museum of Nature, Ottawa, Ontario, Canada; IVPP, Institute of Vertebrate Paleontology and Paleoanthropology, Beijing, China; OMNH, Sam Noble Oklahoma Museum of Natural History, Norman, Oklahoma, United States of America.

### Conventions

Within this paper, we use the following explicit phylogenetic definitions implemented by previous authors (see also Figure 1). Neoceratopsia Sereno 1986 [36] includes all ceratopsians closer to *Triceratops* than to *Psittacosaurus* Osborn 1923 [37], and Coronosauria Sereno 1986 [36] is defined as the most recent common ancestor of *Protoceratops* and *Triceratops* as well as all of the descendants of this ancestor [38]. Ceratopsoidea Hay 1902 [39] includes *Triceratops* and all taxa closer to it than to *Protoceratops* [38]. Leptoceratopsidae Nopsca 1923 [40] includes *Leptoceratops gracilis* Brown 1914 [41] and all species closer to it than to *Triceratops horridus* Marsh 1889 [5], [42]. All ages presented herein follow the Geological Time Scale 2012 [43].

### Permits

No permits were required for the described study, which complied with all relevant regulations.

### Nomenclatural Acts

The electronic edition of this article conforms to the requirements of the amended International Code of Zoological Nomenclature, and hence the new names contained herein are available under that Code from the electronic edition of this article. This published work and the nomenclatural acts it contains have been registered in ZooBank, the online registration system for the ICZN. The ZooBank LSIDs (Life Science Identifiers) can be resolved and the associated information viewed through any standard web browser by appending the LSID to the prefix “http://zoobank.org/”. The LSID for this publication is: urn:lsid:zoobank.org:pub:C835BEAB-3A4C-47B5-8B23-1A244086B3D7. The electronic edition of this work was published in a journal with an ISSN, and has been archived and is available from the following digital repositories: PubMed Central, LOCKSS.

## Results

### Systematic Paleontology

Dinosauria Owen 1842 [44]

Ornithischia Seeley 1887 [45]

Ceratopsia Marsh 1890 [46]

Neoceratopsia Sereno 1986 [36]

*Aquilops* gen. nov.

urn:lsid:zoobank.org:act:B339924F-A48D-4125-B793-9396ECE7891D

*Aquilops americanus* sp. nov.

urn:lsid:zoobank.org:act: 4F4D937E-855B-4D81-A73E-562A145CF358

#### Holotype

**Figure 4:**
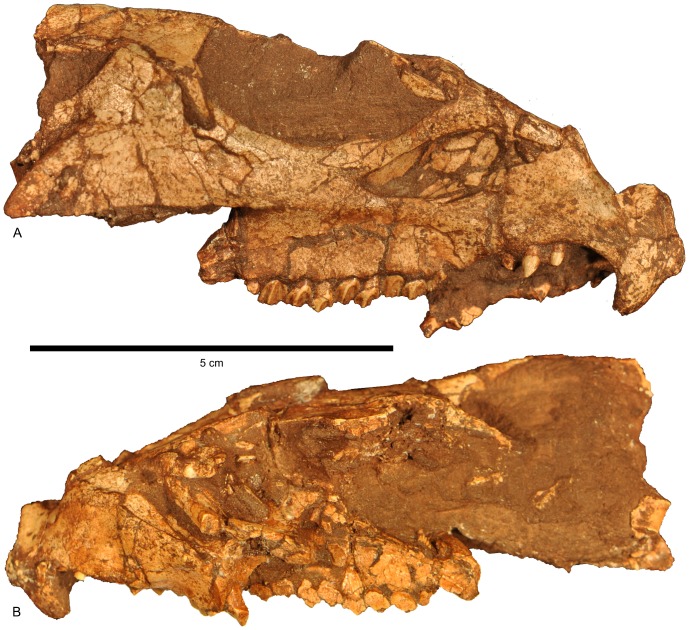
Cranium of *Aquilops americanus*, OMNH 34557 (holotype). A) right lateral and B) left lateral views.

**Figure 5:**
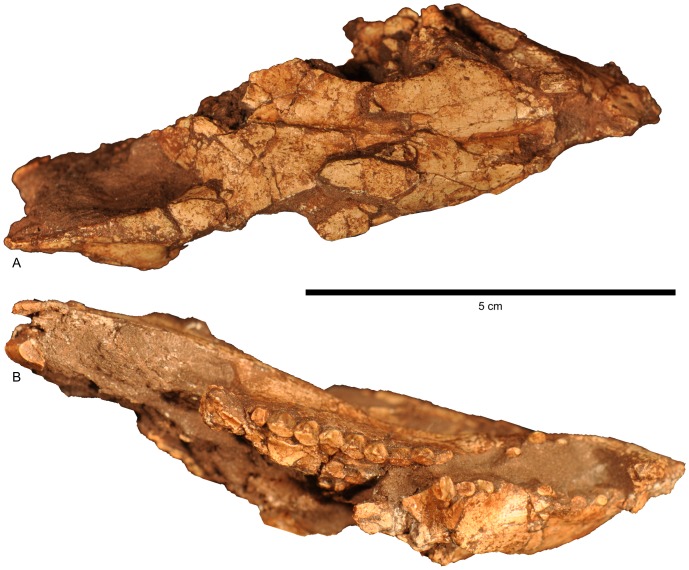
Cranium of *Aquilops americanus*, OMNH 34557 (holotype). A) dorsal and B) ventral views. The rostral end of the skull is to the right side of the image.

**Figure 6:**
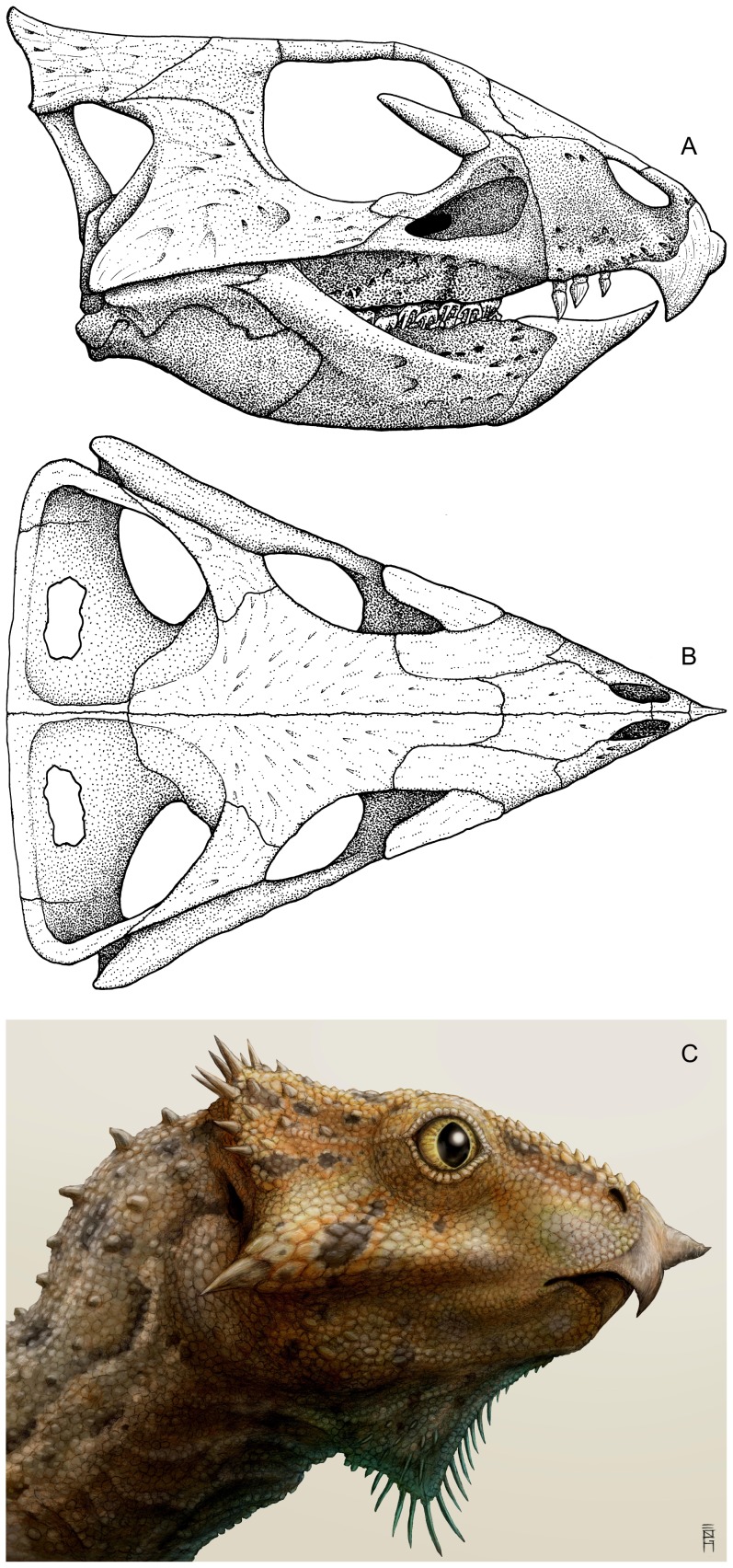
Cranial reconstruction and life restoration of *Aquilops americanus*. Cranium in A) right lateral and B) dorsal views; C) life restoration in right lateral view. The rendering is based on OMNH 34557 (holotype), with missing details patterned after *Liaoceratops yanzigouensis* and *Archaeoceratops oshimai*. Life restoration by Brian Engh.

**Figure 7:**
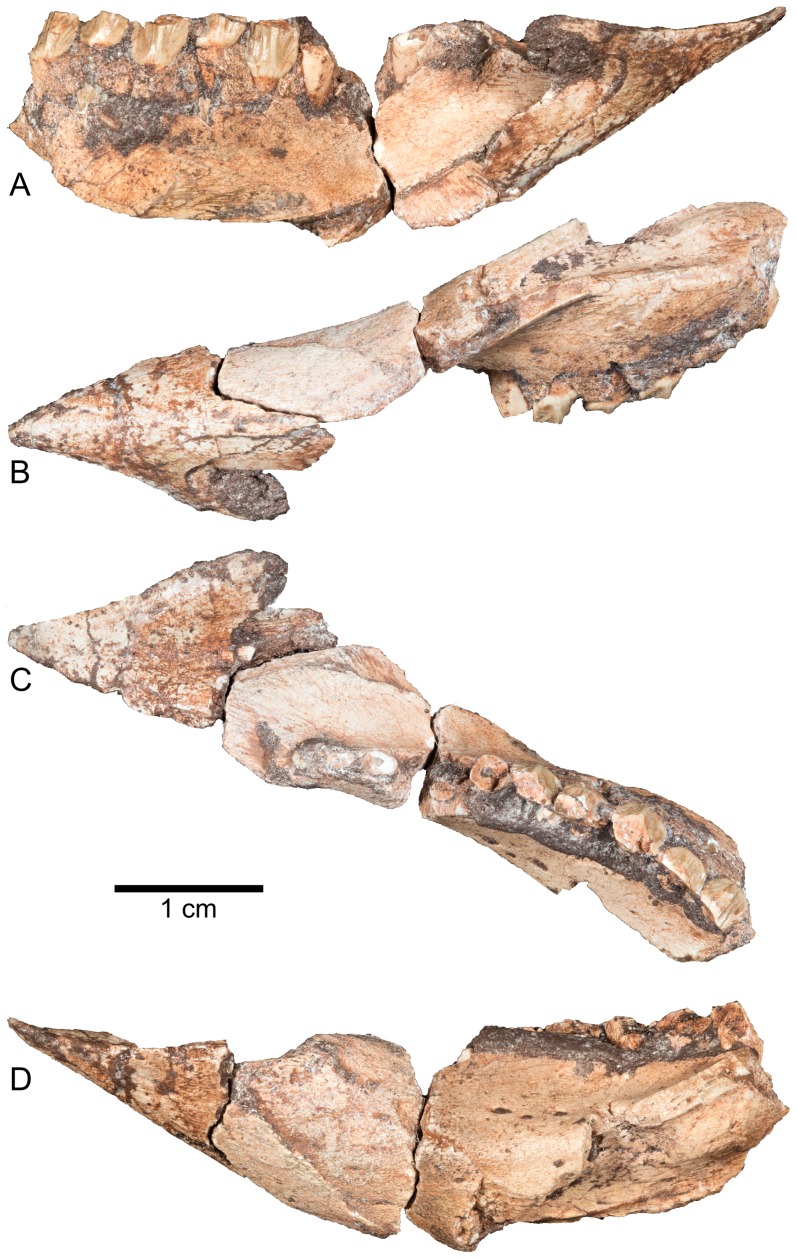
Lower jaw of *Aquilops americanus*, OMNH 34557 (holotype). Predentary and left dentary in A) medial; B) ventral; C) dorsal; and D) lateral views. The three fragments, although unattached, were placed into articulation for these photos. The rostral direction is to the right in A and to the left in B–D.

**Figure 8:**
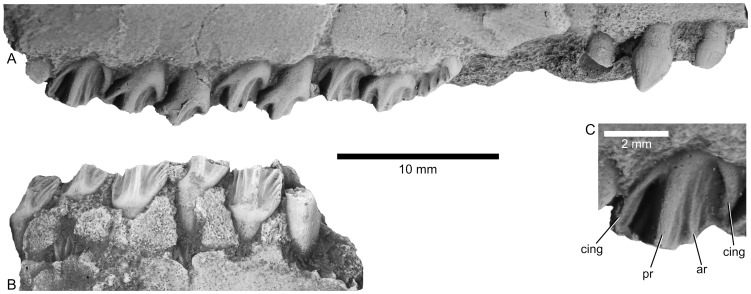
Dentition of *Aquilops americanus*, OMNH 34557 (holotype). A) Upper right dentition in buccal view. B) Lower left dentition in lingual view. C) Detail of maxillary tooth in buccal view, showing selected features. The photographs shown here were taken of casts coated with ammonium chloride, to even out color variations in the specimen. Abbreviations: ar, accessory ridge; cing, cingulum; pr, primary ridge.

**Figure 9:**
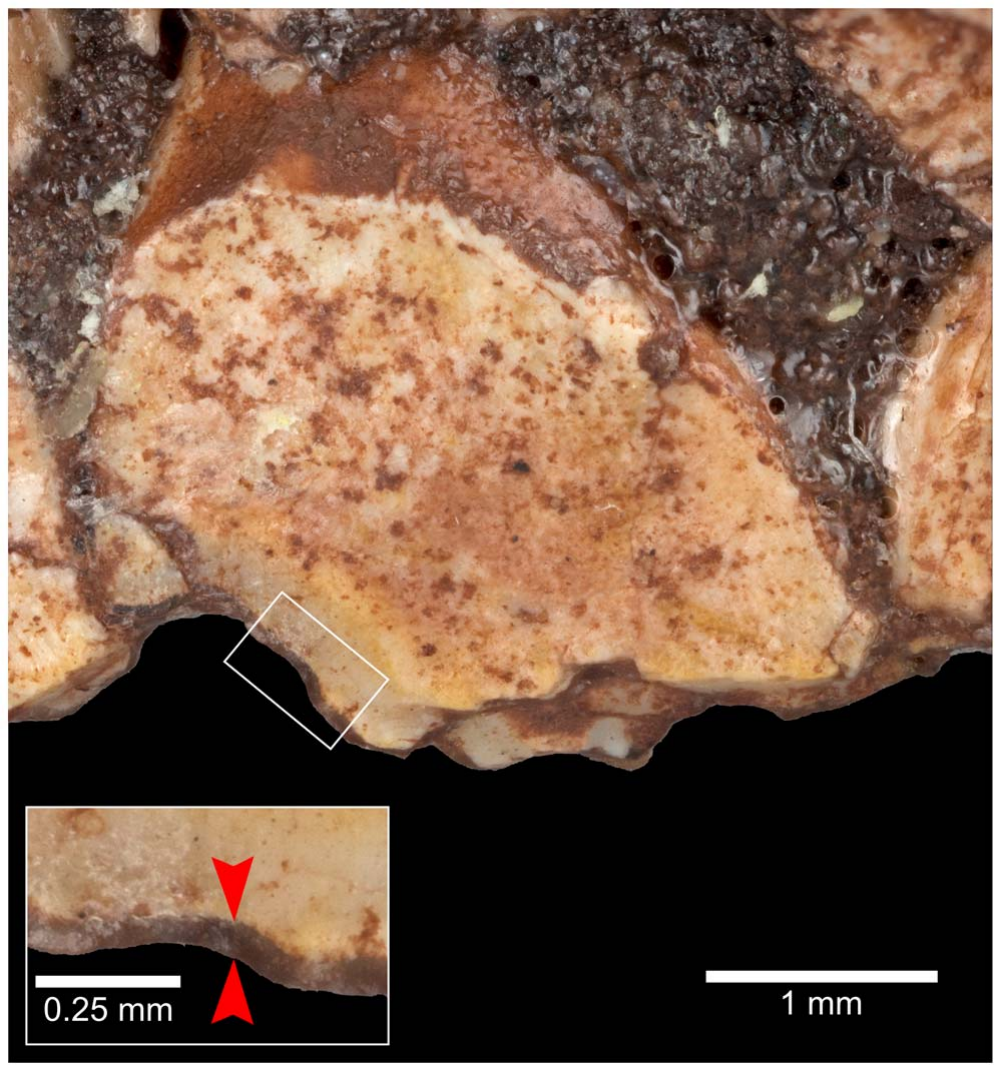
Occlusal (lingual) surface of seventh right maxillary tooth in *Aquilops americanus*, OMNH 34557 (holotype). Note that enamel (highlighted by arrows in the inset) only occurs on the labial surface, and does not continue to the buccal extremity of the tooth at the bottom of the image. The discoloration adhering to the tooth at the top of the image (lingual side of the tooth) is sediment impregnated with consolidant resin, not enamel.

OMNH 34557, a partial skull, with associated predentary, partial left dentary, and additional associated but unidentifiable fragments (Figs. 3–9; three-dimensional digital scans are contained in Files S7–S12).

#### Etymology

The genus name is derived from the Latin *aquila*, meaning “eagle,” and the Greek *ops*, meaning “face,” referring to the hooked beak on the skull of the animal. The species name, meaning “American” (Latin), reflects the species' status as the earliest unequivocal neoceratopsian in North America.

#### Locality and horizon

OMNH locality V1057, 45°N 109°W, Carbon County, Montana, United States of America (Figure 2); basal (?middle–late Albian) part of Unit VII (as defined by [20]), Cloverly Formation. Precise locality data are on file at OMNH and are available to qualified investigators upon request.

#### Diagnosis

A small neoceratopsian with the following autapomorphies: rostral with a ventrally-directed tip and a strongly arched keel possessing a boss; oral margin rostral to maxillary tooth row entirely concave in lateral view; antorbital fenestra more than twice as long as tall and tapering to a sharp point below the orbit.

#### Differential diagnosis

In addition to the autapomorphies listed above, *Aquilops americanus* is distinguished from non-neoceratopsians (e.g., *Yinlong downsi* Xu et al. 2006 [6], *Chaoyangsaurus youngi* Zhao et al. 1999 [47], *Psittacosaurus mongoliensis* Osborn 1923 [37]) by a sharply keeled rostral with a ventral process and sharp keel on rostral edge; exclusion of the postorbital from the margin of the infratemporal fenestra; maxillary teeth with a weak and wide median ridge; and tooth crowns that are ovate in lateral view with enamel restricted to one side, among other features. *Aquilops americanus* is distinguished from *Liaoceratops yanzigouensis* by the suborbital ramus of the jugal being as deep as the orbital ramus; a rounded edge (rather than a sharp ridge) to the predentary oral margin; and the presence of pronounced cingula on the maxillary teeth. *Aquilops americanus* is distinguished from *Yamaceratops dorngobiensis* Makovicky and Norell 2006 [3] by the sharp keel on the rostral surface of the rostral and cheek teeth with cylindrical roots and pronounced cingula. *Aquilops americanus* is distinguished from *Helioceratops brachygnathus* Jin et al. 2009 [48] by having a less steeply inclined ventral predentary facet and a more shallow dentary. *Aquilops americanus* is distinguished from *Archaeoceratops* spp. Dong and Azuma 1997 [49] on the restriction of enamel to one side of the tooth, and differs from *Archaeoceratops oshimai* Dong and Azuma 1997 [49] in the absence of a convexity along the oral margin at the premaxilla/maxilla suture in lateral view. *Aquilops americanus* is distinguished from *Auroraceratops rugosus* You et al. 2005 [8] by a rounded beveled edge on the cutting surface of the predentary. *Aquilops americanus* is distinguished from Leptoceratopsidae by a postorbital with rounded dorsal part overhanging lateral edge of supratemporal fenestra; weak and wide median ridge on maxillary teeth (except for *Cerasinops hodgskissi* Chinnery and Horner 2007 [50]); and cheek teeth with cylindrical roots. *Aquilops americanus* is distinguished from Coronosauria in having a flat, thin, and unmodified dorsum of the frontal; three teeth in the premaxilla; and teeth with cylindrical roots and a weak rather than distinct primary ridge.

### Description

#### Cranium

As preserved, the sagittal plane of the cranium in OMNH 34557 (Figures 3–5) is moderately skewed to the right, along with some moderate mediolateral compression and minor dorsoventral compression (primarily affecting the dorsal portion of the skull). The right side of the skull (Figures 3B, 4A) is better preserved and more complete than the left side (Figure 4B). Elements missing from the right side include the quadratojugal, and most of the quadrate and parietal. The left side preserves only the premaxilla, prefrontal, nasal, partial maxilla, partial frontal, and partial lacrimal. The midline rostral bone is also preserved. The rostral end of the nasals, the braincase, and most of the palatal bones are missing. As preserved, the skull measures 84.2 mm long from the tip of the rostral to the tip of the jugal. Basic measurements for the cranial bones and dentition are in Tables 1 and 2.

**Table 1:** Measurements of cranial bones for OMNH 34557, the holotype of *Aquilops americanus*.

Element	Dimension	Measurement (mm)
Skull	maximum length, tip of rostral to tip of jugal	84.2
Orbit	maximum length	32.0
Antorbital fossa	maximum cranio-caudal length	19.8
	maximum dorsoventral height	8.6
	maximum depth	3.5
Bony naris	maximum length (incomplete)	10.5
Palpebral	maximum length	14.7
Postorbital	rostrocaudal length	27.6
	maximum dorsoventral height	18.8
	maximum mediolateral width	6.1
Jugal	maximum length from distal tip to ventral margin of orbit	31.3
	maximum width of rostral limb	10.4
	maximum width of caudal limb	12.5
Rostral	maximum length of oral margin	11.4
	maximum length of rostral margin	17.9
Premaxilla	maximum length	22.7
	maximum height	20.0
	maximum width across oral margin (distorted)	11.7
Nasal	maximum length (incomplete)	19.1
	maximum width across pair	18.9
Frontals	minimum width across pair at orbits	15.6
Maxilla	maximum length at ventral edge	38.8
	maximum height	15.7
	maximum length of tooth row	29.8
Dentary (left)	maximum height below tooth row	15.2
	maximum length of tooth row (incomplete)	30.3
Predentary	maximum length along ventral edge	22.6
	maximum width of oral margin	12.7
	maximum length along oral margin, right side	19.1
	maximum depth of “scoop” on dorsal surface	3.0

All measurements are from the right side unless otherwise indicated.

**Table 2:** Measurements of dentition for OMNH 34557, the holotype of *Aquilops americanus*.

Tooth	Dimension	Measurement (mm)
Premaxillary tooth 1	apical-basal height, crown (worn)	2.3
	mesio-distal length, crown	2.1
Premaxillary tooth 2	apical-basal height, crown	3.4
	mesio-distal length, crown	2.6
	lingual-buccal width, crown	1.8
Premaxillary tooth 3	mesio-distal length, crown (impression)	1.6
Maxillary tooth 2	mesio-distal length, crown	3.4
Maxillary tooth 4	mesio-distal length, crown	3.3
Maxillary tooth 6	mesio-distal length, crown (replacement)	3.2
	apical-basal height, crown (replacement)	4.8
Maxillary tooth 8	mesio-distal length, crown	3.9
Dentary tooth 6	mesio-distal length, crown	3.7
Dentary tooth 8	mesio-distal length, crown	3.9
Dentary tooth 9	mesio-distal length, crown	4.0
Dentary tooth 10	mesio-distal length, crown	3.9
	apical-basal height, crown	3.7

All measurements for upper dentition are from the right side and all for lower dentition are from the left side. Unless otherwise indicated, all measurements are for functional teeth.

The dorsal margin of the preorbital portion of the skull was steeply inclined as compared to the postorbital portion (Figures 3B, 4A, 6A). Despite the crushing, the breadth of the frontals relative to the surrounding parts of the skull strongly suggests that the skull was triangular in dorsal view (Figures 3A, 5A, 6B), although perhaps not as strongly so as in larger species. This reduced prominence in triangularity may in part be due to the possible juvenile nature of the specimen, as seen in *Protoceratops* ([51]; see below).

#### Rostral

The rostral bone is unelaborated compared to the condition in ceratopsids (e.g., *Triceratops horridus*), with short dorsal and lateral (buccal) processes (Figure 3B). Part of the dorsal process on the right side is broken away, but the sutural surface with the premaxilla remains, allowing confident reconstruction of the original morphology in the holotype. The rostral (anterior) surface is gently keeled, more prominently than seen in *Yamaceratops dorngobiensis* (see [3]) but less than the condition in *Protoceratops andrewsi* or *Leptoceratops gracilis*. A midline boss or rugosity (5.3 mm long by 1.2 mm tall) caps the leading edge of the rostral, unique among ceratopsians. Furthermore, the lateral profile of the leading edge is strongly arched, contrasting with the gently arched leading edge seen in other ceratopsians (e.g., *Psittacosaurus mongoliensis*, AMNH 6254; *Archaeoceratops oshimai*, IVPP V11114; *Protoceratops andrewsi*, AMNH 6466). In lateral view, the ventral margin of the rostral is strongly hooked. As articulated with the premaxilla, the tip of the rostral forms a nearly 90 degree angle with the line of the maxillary teeth. This feature is autapomorphic for *Aquilops*. Although *Archaeoceratops oshimai* has been illustrated with a similar condition ([7]: fig. 1), direct examination of the original specimen (IVPP V11114) shows that the degree of hooking was exaggerated in the published drawings. Thus, the morphology for the holotype of *A. americanus* is indeed unique. In other neoceratopsians, the angle of the rostral margin relative to the maxillary teeth is much broader (e.g., ∼150 degrees in *Liaoceratops yanzigouensis*, IVPP V12738).

#### Premaxilla

The premaxilla is roughly triangular in lateral view. The ventral border is straight, with the caudal third supporting three teeth (described below). There is no evidence of a prominent bump or convexity in the oral margin at the interface between the maxilla and premaxilla, unlike the condition in many other ceratopsians (e.g., *Chaoyangsaurus youngi*, CAGS-IG V371; *Archaeoceratops oshimai*, IVPP V11114; *Leptoceratops gracilis*, CMN 8887, 8889; *Protoceratops andrewsi*, AMNH 6466; [7], [47]), and paralleling the morphology seen in *Liaoceratops yanzigouensis* (IVPP V12738). The articulated maxilla, premaxilla, and rostral together produce a unique concave profile to the oral margin rostral to the maxillary teeth (Figures 3B, 4, 6). A slight lateral swelling occurs immediately dorsal to the oral margin and is confluent with the rostral bone. The swelling terminates at its tallest point just rostral to the premaxillary-maxillary suture and tapers rostrally. As in *A. oshimai* and other non-coronosaurian neoceratopsians, the caudal margin of the premaxilla is vertical and forms part of the rostral border of the antorbital fossa. As preserved, the external naris is 9 mm long and is therefore quite small in proportion to the orbit and antorbital fossa (Table 1), as is typical for non-ceratopsids (e.g., *Liaoceratops yanzigouensis*; *Protoceratops andrewsi; Yinlong downsi*). The naris is oriented at a 60 degree angle to horizontal, and the preserved parts of the narial margin indicate an overall elliptical shape. The naris is confluent with a shallow depression on the rostral end of the premaxilla.

In ventral view (Figure 5B), the oral margin of the paired premaxillae is broadest at its midpoint, and is pinched cranially (to receive the rostral bone) and caudally (at the suture with the paired maxillae). Accounting for distortion, the alveoli for the premaxillary teeth would have been in line with, and not outside of, many of the maxillary teeth, as in other ceratopsians with premaxillary teeth. Although the teeth in the holotype of *Archaeoceratops yujingziensis* (CAGS-IG-VD-003) are described as falling outside the line of the maxillary teeth [52], this appears to be a function of specimen orientation rather than genuine morphology; thus the anatomy in *A. yujingziensis* probably matches that in other ceratopsians.

#### Maxilla

The maxilla is elongate and roughly trapezoidal in lateral view, bounded dorsally by the jugal and lacrimal (Figure 3B). A prominent buccal emargination characterizes the lateral surface of the maxilla; it slopes from its highest point caudally to the rostral end, where the emargination intersects with the oral margin at the maxillo-premaxillary suture. At least four neurovascular foramina mark the lateral surface of the emargination. As typical for non-ceratopsids, the postalveolar process is quite short, extending only 5.4 mm behind the last tooth position. In ventral view (Figure 5B), the alveolar margin is slightly sinuous, with a moderate lateral concavity at the cranial third of the element. The right and left maxillae are closest to each other at their rostral ends, with a moderate lateral deflection at the premaxillary suture, as seen in many other basal neoceratopsians (e.g., *Archaeoceratops* spp. [CAGS-IG-VD-003; IVPP V11114]; *Auroraceratops rugosus*, CAGS-IG-2004-VD-001; *Liaoceratops yanzigouensis*, IVPP V12738; [52]). The tooth row is also set slightly below the premaxilla in lateral view, and the maxillary teeth are oriented horizontally (Figures 3B, 6A). Although this is slightly accentuated by crushing, the profile does not seem to be due solely to taphonomic processes.

#### Nasal

The rostral ends of the paired nasals are missing, and crushing has slightly obscured their original shape and sutural relationships (Figure 3A). The caudal ends of the nasals contact a narrow rostral process of the paired frontals, completely separating the prefrontals from each other. The dorsal surface of the nasals is relatively flat and untextured, as seen in many other non-coronosaurs (e.g., *Archaeoceratops oshimai*, IVPP V11114; *Yinlong downsi*, IVPP V14530). Although there is some crushing on the right nasal, the dorsal surface of the left nasal is quite flat, showing that OMNH 34557 lacks a fossa on the dorsal surface, as seen in *Liaoceratops yanzigouensis* (IVPP V12738; [1]).

#### Palpebral

The palpebral is broadest at its attachment with the prefrontal and lacrimal, tapering to a gently rounded profile at its free end (Figures 3A,B, 4A, 5A). The dorsal surface of the element is flat. A loose element, interpreted as the left palpebral, shows a triangular coronal cross-section. Overall, the element in OMNH 34557 is quite similar to the morphology seen in other basal neoceratopsians.

#### Antorbital fossa

The cranio-caudally elongate and triangular antorbital fossa is bordered caudally by the jugal, ventrally by the maxilla, rostrally by the premaxilla (for a very small extent), and dorsally by the lacrimal (Figures 3B, 4A). The fossa is tallest at its rostral end, and the caudal portion of the fossa is deepest. Its ventral border is nearly horizontal. The dorsal edge of the fossa is sharply defined at its portion immediately ventral to the orbit, with a distinct bar of bone. This distinction becomes less defined in the rostral direction. Compared to other ceratopsians, the antorbital fossa in OMNH 34557 is elongate, with a height:length ratio of 0.43 (measurements in Table 1). This compares to 0.73 in *Archaeoceratops oshimai*, IVPP V11114 (20.8 by 28.5 mm on the better preserved right side), and 0.72 in *Auroraceratops rugosus*, CAGS-IG-2004-VD-001 (20.6 by 28.7 mm on the left side), and the relatively equidimensional, circular fossae in leptoceratopsids and protoceratopsids.

#### Lacrimal

Ventral to the orbit, the lacrimal inserts into the jugal along a V-shaped contact (Figures 3B, 4A), a condition that is widespread across Neoceratopsia (e.g., *Liaoceratops yanzigouensis*, IVPP V12738; *Archaeoceratops oshimai*, IVPP V11114; *Leptoceratops gracilis*, CMN 8889; *Protoceratops andrewsi*, AMNH 6429, 6637). In most specimens belonging to *Psittacosaurus*, the contact is fairly linear (see figures in [53]).

#### Prefrontal

The prefrontal is roughly hatchet-shaped and is narrowest caudally, where it forms the dorsal margin of the orbit, maintaining a relatively uniform width at the portion between the nasal and lacrimal, and expanding laterally at the rostral end to contact the premaxilla (Figure 3A). The dorsal surface of the caudal end of the prefrontal is flat, and it is gently concave at the rostral end.

#### Frontal

The preserved parts of the paired frontals are roughly diamond-shaped in dorsal view (Figure 3A), with a very slight ridge running the length of the interfrontal suture. A similarly slight (<0.5 mm deep) depression parallels this ridge. Otherwise, the dorsal surface of the frontals is quite flat, typical of ceratopsians outside of Leptoceratopsidae and Coronosauria [54]. A narrow prong inserts between the prefrontals and contacts the nasals rostrally (as seen in other basal ceratopsians, such as *Psittacosaurus* spp. and *Liaoceratops yanzigouensis*, IVPP V12738; [53]). Some very faint neurovascular grooves originate in the middle of the dorsal surface of each frontal, approximately at the midpoint of the orbit, and radiate rostrally for several millimeters. Caudally, the suture between the parietals and the frontals is not discernible, assuming that it is even preserved. Thus, it cannot be determined what proportion of the rostral end of the supratemporal fenestra was formed by the frontal and parietal, respectively. The broken edges of the frontal along the caudal end of the bone uniformly approximate only a millimeter in thickness.

#### Postorbital

The postorbital is longer than tall or wide (Figures 3A,B, 4A, 5A). The dorsal and lateral surfaces of this bone are nearly at right angles to each other, separated by a prominent ridge. The dorsal surface of the postorbital is flattened and forms part of the lateral margin of the supratemporal fenestra; the narrow bone tapers caudally along the fenestra's margin. The postorbital abuts the caudal margin of the frontal. The lateral surface of the bone is also flattened, with strong tapers to both the caudal and descending limbs. This latter portion of the postorbital is sandwiched between the orbit and the ascending limb of the jugal. Although there is some cracking in this area, a thin flange of the jugal does seem to separate the postorbital from the infratemporal fenestra (Figure 3B). Consequently, the postorbital is excluded from the infratemporal fenestra as in other non-coronosaurian neoceratopsians (e.g., *Yamaceratops dorngobiensis,* IGM 100/1303; *Liaoceratops yanzigouensis*, IVPP V12738).

#### Jugal

The jugal is almost perfectly planar along most of its lateral surface, except for a prominent ridge and concavity immediately rostral to the infratemporal fenestra (Figures 3B, 4A). The horizontally-oriented rostral limb of the element is comparatively longer and narrower than the ascending, caudal limb. The “blade” of the jugal continues the horizontal orientation of the rostral limb, terminating in a point. No epijugal scar is visible on the specimen, and we thus infer that an ossified epijugal was lacking. This condition also occurs in non-neoceratopsians, *Liaoceratops yanzigouensis* (IVPP V12633, V12738; [1]), and juvenile coronosaurs (e.g., *Protoceratops andrewsi* and *Triceratops* sp. [51], [55]). The caudal margin of the jugal, marked by a prominent concavity, is oriented at approximately 45 degrees to the horizontal. Several lightly incised neurovascular impressions occur in this area, differing from the more heavily sculptured surface seen in most other ceratopsians (e.g., *Yinlong downsi*, IVPP V14530; *Archaeoceratops oshimai*, IVPP V11114; *Liaoceratops yanzigouensis*, IVPP V12738). This may be a juvenile feature of OMNH 34557, as for juvenile ceratopsids [56].

#### Orbit

The exact profile of the orbit is obscured by crushing, although it appears that the orbit was originally longer than tall (Figures 3B, 4A, 6A). The rostral one-third of the ventral margin of the orbit is bound by the lacrimal; the jugal bounds the caudal two-thirds. The caudo-dorsal quadrant of the orbit is bounded by the postorbital, with the remaining dorsal quarter bounded by (from caudal to cranial) the frontal, prefrontal, and palpebral. Relative to the preorbital length, the orbit is comparatively large (32 mm vs. 38 mm, or a ratio of 0.84), indicative of juvenile ontogenetic status in many vertebrates, including ceratopsians [51], [57], [58]. By comparison, the presumably adult holotype of *Archaeoceratops oshimai* (IVPP V11114) has a ratio of 0.63 (47 mm vs. 74 mm) and the holotype of *Auroraceratops rugosus* (CAGS-IG-2004-VD-001) has a ratio estimated at 0.64 (52 mm vs. 81 mm).

#### Ectopterygoid

The right ectopterygoid, missing its caudal end, is visible in lateral, medial, and caudal views (Figures 3B, 4A,B). As is typical for non-ceratopsid neoceratopsians (e.g., *Liaoceratops yanzigouensis*; IVPP V12738; *Protoceratops andrewsi*, AMNH 6429), the element is wrapped around the caudal surface of the alveolar process of the maxilla.

#### Quadrate

A fragment of the shaft of the right quadrate is in place near the ventral margin of the right jugal. The cross-section of the ventral edge of the fragment is D-shaped, with the convex surface pointing caudally.

#### Squamosal

The preserved fragment of the right squamosal forms part of the supratemporal fenestra and is triangular, broadening caudally. The squamosal is situated dorsal and caudal to the postorbital (Figure 3A,B).

#### Predentary

The predentary tapers to a sharp point both in dorsal and lateral views (Figures 3C,D, 7), contrasting with the blunt, U-shaped predentary in psittacosaurids and *Liaoceratops yanzigouensis*, but similar to the condition in all other neoceratopsians [3]. In *Archaeoceratops oshimai* (IVPP V11114) and *Auroraceratops rugosus* (CAGS-IG-2004-VD-001), part of the oral margin is canted dorsolaterally [8], [59], contrasting with the condition in *Liaoceratops yanzigouensis* (IVPP 12738) and *Yamaceratops dorngobiensis* (IGM 100/1867). The latter morphology occurs in OMNH 34557, in which the oral margin is relatively indistinct and faces dorsally to dorsomedially. Thus, the dorsal cutting surface of the predentary smoothly grades into the medial, shallowly scooped aspect of the bone. This shallow scooping is most similar to the condition in basal ceratopsians (e.g., *Archaeoceratops oshimai*, *Chaoyangsaurus youngi*) and differs from the deeply invaginated dorsal midline of leptoceratopsids and coronosaurs [3]. Like all of the aforementioned neoceratopsians, but unlike non-neoceratopsians, *Aquilops americanus* has a blunt rather than a sharp, ridge-like oral margin for the predentary. The ventral process of the predentary is much more prominent and broader than the dorsal process. The left and right lateral surfaces of the predentary meet ventrally along a rounded keel (as in most neoceratopsians), unlike the broadly oval cross-section seen in *Liaoceratops yanzigouensis* (IVPP V11114) and non-neoceratopsians.

#### Dentary

Two fragments represent this element, with a clean contact fit between each other and the predentary (Figures 3C,D, 7). The lingual surface of the incomplete left dentary is convex caudally and concave rostrally, preserving the rostral portion of the Meckelian groove (Figure 7A). This groove is quite broad, as in other neoceratopsians. In dorsal view, the tooth row on the left dentary fragment is concave laterally (Figures 3C, 7C). The dorsal portion of the lateral surface of the dentary shows a strong buccal emargination, and thus the tooth row is greatly inset. The dentition extends rostrally up to the point of the suture for the predentary, so that the length of the diastema at the rostral end of the dentary was minimal (Figures 3D, 7C). This morphology is typical of basal ceratopsians but unlike that in ceratopsids [3].

#### Splenial

Although the splenial itself is not preserved, its sutural surface with the dentary is discernible on that element. The very rostral end of the splenial contacted the ventral process of the predentary (Figures 3D, 7A,C). Thus, based on the configuration of the predentary, splenial, and dentary contacts, it can be inferred that the contact between the dentaries at the midline was minimal, as is typical of non-ceratopsids [59]. The splenials also presumably contacted each other along the midline, although this cannot be verified.

#### Dentition

The premaxilla contains three ventrally-directed teeth, with the rostral-most tooth substantially smaller than the caudal two teeth (Figures 3B, 4A, 8A). In buccal view, the premaxillary teeth are roughly teardrop-shaped, broadest at the crown base and gently tapering toward the tip (Figure 3A). The base of each premaxillary tooth crown is mesiodistally elongate, and a carina marks the rostral and caudal edges of each tooth's crown. The crown lacks denticles (unlike the teeth in *Archaeoceratops yujingziensis*, *Liaoceratops yanzigouensis*, and *Yamaceratops dorngobiensis*; [3],[52],[60]), and both buccal and lingual surfaces bear enamel. The first premaxillary tooth on the right side is slightly rounded at its apex, suggestive of a wear facet. No wear facet is evident on the second premaxillary tooth, although this may be a function of relatively recent eruption.

The right maxilla bears nine closely-packed teeth (Figure 8A), which are separated from the premaxillary teeth by a 6 mm diastema. The teeth in the middle tooth positions are largest. In buccal view, each tooth crown tapers towards its occlusal end. A broad, distally-placed primary ridge divides the buccal surface of each crown, with at least one less pronounced accessory ridge distally and one or two mesially (Figure 8C; as seen in *Archaeoceratops oshimai*, IVPP V1114, and *Auroraceratops rugosus*, CAGS-IG-2004-VD-001) but unlike the very weakly developed accessory ridges and centrally placed primary ridge of *Liaoceratops yanzigouensis* (IVPP V12633) and non-neoceratopsians [60]. The primary ridge merges with a well-defined basal cingulum on each tooth (Figure 8C). At least three “waves” (Zahnreihen of Edmund [61]) of teeth, moving from back to front, are preserved. The unworn crowns bear at least three denticles on each of their occlusal edges, but none of the unworn teeth is sufficiently exposed to allow a more detailed description of this feature. On worn teeth, three to four “cusps” are present, representing the occlusal expression of the buccal ridges. Only the buccal surface of each tooth crown bears enamel (Figure 9; the derived condition within Neoceratopsia, exclusive of *L. yanzigouensis*), and the wear surface of the maxillary teeth is angled laterally from base to tip, deviating approximately 20–30 degrees from the vertical.

Twelve incomplete, closely-packed teeth and tooth roots are preserved in the left dentary fragments (Figures 3C,D, 7, 8B). Here, the major ridge on the lingual surface is mesially placed with respect to the center of each tooth crown and is much more weakly developed than in the maxillary teeth. Weaker subsidiary ridges on each side of the primary carina terminate in fine denticles (3–4 distally, 4–5 mesially) at the occlusal edge of each dentary tooth. Similarly, the cingula are much more poorly developed in the dentary teeth than in the maxillary teeth; this condition also occurs in *Archaeoceratops oshimai* (IVPP V11114). Only the lingual surfaces of the tooth crowns bear enamel. As noted for *Yamaceratops dorngobiensis* (see [3]), the mesial ridges are angled relative to the primary ridge whereas the distal ridges are subparallel to the primary ridge. Replacement teeth are visible at two points ventral to the functional teeth; there does not appear to be room for a second row of underlying replacement teeth. Although no complete, unworn tooth crowns are visible, the maxillary and dentary teeth generally seem proportionately broader mesiodistally than in *Archaeoceratops* spp. [52], [60]. Based on the diameter of the preserved portions, the rostral-most dentary teeth were smaller than those more caudally placed.

The number of dentary teeth greatly exceeds the number of maxillary teeth (9 and at least 12, respectively) in OMNH 34557. This condition is shared with *Liaoceratops yanzigouensis* of all known ontogenetic stages (11 or 12 vs. 15 maxillary and dentary teeth in IVPP V12738, the largest described skull; 10 vs. 12 in CAGS-IG-VD-002, the smallest described skull). In other basal neoceratopsians, the maxillary and dentary tooth counts are more closely matched (e.g., 13 vs. 12, respectively, in *Auroraceratops rugosus*, CAGS-IG-2004-VD-001; 13 or 14 vs. 14 in *Archaeoceratops oshimai*, IVPP V11114; 17 vs. 16 teeth in *Leptoceratops gracilis*, CMN 8889).

#### Ontogenetic status of OMNH 34557

The holotype for *Aquilops americanus* is approximately 60 percent the size of the presumed adult holotypes for *Liaoceratops yanzigouensis* (IVPP V12738) and *Archaeoceratops oshimai* (IVPP V11114), but about the same size as a referred juvenile specimen of *L. yanzigouensis* (IVPP V12633), judging by preorbital length. Additionally, the orbit in OMNH 34557 is comparatively large relative to the rest of the skull, and the cranial bones are minimally textured. Based on reconstructed ontogenetic series for other ceratopsians, this suggests that OMNH 34557 is not fully grown. However, we also note that some features of the *Aquilops americanus* holotype are found in many adult basal neoceratopsians but not necessarily juveniles (e.g., well-defined accessory ridges on maxillary teeth; elongated contact between jugal and lacrimal). Thus, the mix of potential juvenile and adult features as well as intermediate size suggests identification of OMNH 34557 as “subadult” (comparable to assessments made for *Protoceratops* [62]). We hypothesize that the autapomorphies seen in the specimen as well as the unique combination of other features (even relative to known juveniles) support naming and diagnosis of *Aquilops americanus*.

#### Phylogenetic analysis

In order to assess its position within Ceratopsia, *Aquilops* was scored using previously published matrices, with additions and revisions as noted below. Characters 1–133 were taken from Makovicky and Norell [3], characters 134–147 were taken from Makovicky [63], characters 148 and 149 were taken from characters 135 and 136 of Lee et al. [64], and characters 150 and 151 are from Ryan et al. [65]. In order to help resolve Ceratopsidae, characters 152 (circumnarial depression, if deep, simple or complex) and 153 (narial spine absent or present) were added. Following Ryan et al. [65], character 140 was replaced with the construction and codings of character 134 from Lee et al. [64]. A full list of characters is provided in Text S1 (File S1). *Ajkaceratops kozmai* Ősi et al. 2010 [66], *Helioceratops brachygnathus*, *Auroraceratops rugosus* You et al. 2005 [8], *Chasmosaurus belli* Lambe 1902 [67], *Diabloceratops eatoni* Kirkland and DeBlieux 2010 [68], *Gryphoceratops morrisoni* Ryan et al. 2012 [65], *Koreaceratops hwaseongensis* Lee et al. 2011 [64], *Turanoceratops tardabilis* Nessov and Kaznshkina in Nessov et a. 1989 [69], *Unescoceratops koppelhusae* Ryan et al. 2012 [65], and *Zhuchengceratops inexpectus* Xu et al. 2010 [70] were also added to the matrix. The codings for character 138 for *Centrosaurus apertus* Lambe 1904 [71] and *Triceratops horridus* were changed from 1 to 0. Character 6 was recoded for all taxa, to correct a misprint in the original matrix. The codings for characters 17, 52, 71, and 96 were changed from? to 1, and the coding for character 51 was changed to 1 for *Zuniceratops christopheri* [72], following examination of the original material.

The matrix (Text S2 in File S1, File S2) was run in TNT 1.1 [73] using the tree bisection reconnection algorithm, with 10,000 replicates, up to 10,000 trees saved per replication, and branches with a minimum length of 0 collapsed. *Hypsilophodon foxii* Huxley 1869 [74] was set as the outgroup relative to all other taxa, and all characters were unordered. Bremer support values were also calculated, along with bootstrap support (using sampling with replacement and 10,000 replicates).

**Figure 10:**
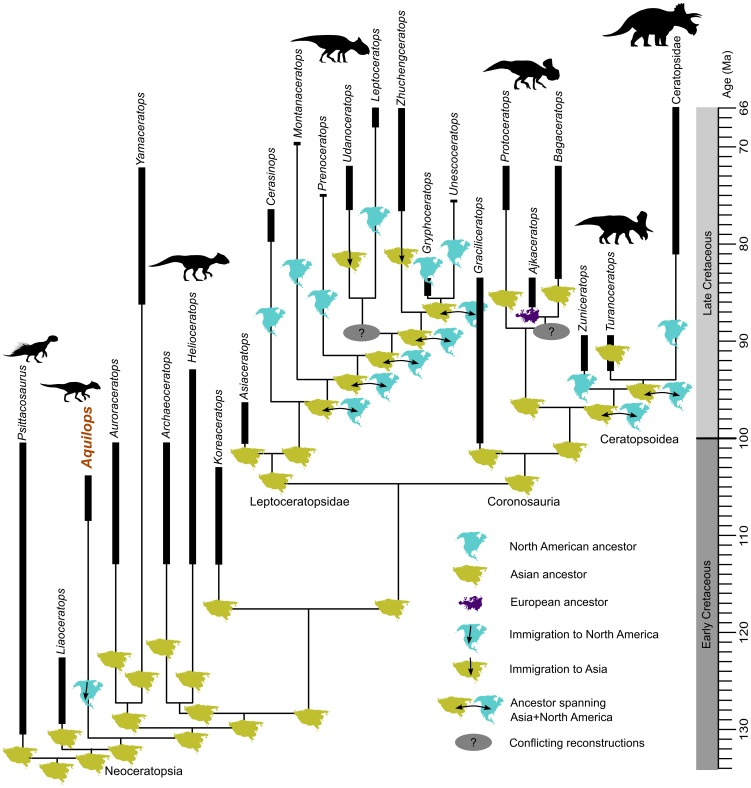
Hypothesis of phylogeny and biogeography for Neoceratopsia. Some terminal taxa have been combined for space considerations, and the range bars for each taxon indicate uncertainty rather than known geological ranges. Continent icons indicate the ancestral areas reconstructed by DEC modeling. Silhouettes depict representative members of major clades and grades (*Psittacosaurus* by J. Headden, *Zuniceratops* by N. Tamura and modified by T. M. Keesey; *Triceratops* by R. Amos; all others by A. Farke; all images are CC-BY and provided via www.phylopic.org). Full results are presented in File S1.

Two equally most parsimonious trees (length  = 288) were recovered, with *Aquilops americanus* firmly within Neoceratopsia (Figure 10; Figures S1 and S2 in File S1). *Ajkaceratops kozmai*, the only taxon to vary in position, was recovered as either a basal neoceratopsian (Figure S1 in File S1) or sister to *Bagaceratops rozhdestvenskyi* (Figure S2 in File S2). Due to the extensive ghost lineages required by the former option, we consider the latter most likely. This uncertainty appears to be due to character conflict as well as the incomplete nature of *Ajkaceratops*. The uncertain position of *Ajkaceratops* resulted in rather low bootstrap values, with most nodes in Neoceratopsia scoring less than 50 percent (Figure S3 in File S1). It took two additional steps to force *Aquilops* as a leptoceratopsid, but it took only one additional step to force *Aquilops* as sister to leptoceratopsids + coronosaurs or sister to coronosaurs alone. Five additional steps were required to force *Aquilops* as a ceratopsoid. The overall structure of the tree is, unsurprisingly, similar to that produced using other recent versions of the matrix [63], [65].

Multiple synapomorphies identify *Aquilops* as a neoceratopsian. In the tree that recovers *Ajkaceratops* as a basal neoceratopsian, these include: a rostral with a ventral process and a keeled face; enlarged antorbital fossa; postorbital excluded from margin of laterotemporal fenestra; wide Meckelian groove separating dentigerous portion of mandible from external surface; predentary buccal margin rounded; maxillary teeth with median ridge; and ovate tooth crowns with enamel restricted to one side. In the tree that recovers *Ajkaceratops* as sister to *Bagaceratops*, an additional two synapomorphies were recognized as placing *Aquilops* within Neoceratopsia, including a convex buccal process rostral to the maxillary tooth row as well as pronounced cingula on cheek teeth.

If OMNH 34557 represents a subadult individual, this could influence the phylogenetic position recovered for *Aquilops*. Juvenile individuals often show primitive character states relative to adults of a species, which may pull operational taxonomic units coded from juvenile material toward the root relative to their actual position or destabilize the tree in other ways (e.g., [75]–[77]). We surveyed ceratopsians for which at least partial growth series were available (including *Psittacosaurus*, *Liaoceratops*, *Protoceratops*, *Bagaceratops*, *Centrosaurus*, and *Triceratops*) in order to evaluate which characters coded for *Aquilops* potentially were ontogenetically labile. The primary relevant, ontogenetically variable characters for the holotype of *Aquilops* were 29 (presence of epijugal) and 98 (presence and morphology of primary ridge on teeth) [3], [62]. Coding these characters as unknown for *Aquilops* (along with character 30, position of epijugal, which was coded as inapplicable in the original matrix; Text S3 in File S1; File S3) resulted in 18 equally most parsimonious trees of 288 steps each (Figures S4 and S5 in File S1). In all of these trees, *Aquilops* was still recovered as a neoceratopsian outside of the clade Leptoceratopsidae + Coronosauria, although its relationship relative to other “basal” neoceratopsians varied. The basal position of *Aquilops* within Neoceratopsia is also congruent with its geological age. Full results are presented in File S1.

#### Biogeographic analysis

In order to evaluate the biogeographic patterns of North American ceratopsians relative to those from other areas, the Dispersal-Extinction-Cladogenesis (DEC) model [78], [79] was used to reconstruct ancestral ranges within Ceratopsia. The DEC model is a likelihood model incorporating temporal data [79], and is increasingly used in studies of dinosaur biogeography (e.g., [80], [81]). Taxa were assigned to one of three biogeographic areas: North America, Asia, and Europe. Although the position of *Ajkaceratops* was variable in the phylogenetic analysis, we used the topology that placed it as sister to *Bagaceratops* rather than the topology that placed it as sister to *Liaoceratops* (Figure S6 in File S1); the former scenario was considered most likely because it minimized the lengths of ghost lineages and also is more congruent with the geological age of *Ajkaceratops*. Taxa outside of Ceratopsia (*Hypsilophodon* and *Stegoceras*) were trimmed from the analysis for two reasons: 1) because the outgroups used in the phylogenetic analyses represent incomplete samples of their respective group diversity and geographic/temporal range; and 2) because Neoceratopsia are the focus of the analysis. The temporal range of each taxon was estimated from the literature, and the midpoint of this range, rounded to the nearest 0.1 Ma, was used as the datum for each species unless more precise data were available (details in Table S1 in File S1; File S4). A time-calibrated phylogeny was produced using the timePaleoPhy function of paleotree 2.0, as implemented within R [82]. Here, the branches were time-scaled using the “aba” method, so that 0.1 Ma were added to all zero-length branches; this effectively minimized ghost lineage lengths (a “strict” time calibration; File S5). A second data set was also produced using the “equal” option within timePaleoPhy, so that the lengths of ghost lineages were more equally distributed and thus longer than the “aba” method (a “smoothed” time calibration; File S6). Analyses were run using Lagrange 2.0, release 20130526 (available from https://github.com/rhr/lagrange-python).

The DEC model reconstructs an Asian origin for nearly all major ceratopsian clades (Figure 10; Table S2 in File S1 and Figure S5 in File S1). The ancestor of *Aquilops* dispersed from Asia into North America. The common ancestor of ceratopsoids is reconstructed as spanning North America and Asia, as for the ancestry of the clade including *Turanoceratops* + Ceratopsidae. Although the common ancestor of leptoceratopsids was most likely an Asian form, the ancestor of most groups within Leptoceratopsidae spanned Asia and North America together. All together, the DEC model as applied here reconstructs three different dispersals into North America from Asia—for *Aquilops* (and perhaps other, more poorly documented occurrences of Neoceratopsia near the Early–Late Cretaceous boundary of North America, noted below), most leptoceratopsids, and ceratopsoids. Full results are included in Table S2 in File S1 and Figure S5 in File S1.

## Discussion

*Aquilops* provides the first phylogenetically diagnostic evidence of a neoceratopsian dinosaur from the Early Cretaceous of North America. Other Early–early Late Cretaceous probable neoceratopsians from North America are represented by teeth from the Arundel Clay of Maryland [10], believed to be early Albian (∼113–110 Ma; [83]); teeth from the uppermost Cedar Mountain Formation of Utah [10], of earliest Cenomanian age (∼98 Ma; [9]) and a partial postcranial skeleton from the middle to upper Albian Wayan Formation of Idaho (∼110–101 Ma; [11]). Though these fossils suggest the geographically widespread presence of Neoceratopsia in North America during the late Early Cretaceous of North America, their affinities within the group are problematic. The new find from the Cloverly Formation, in concert with taxa known from elsewhere, permits a much more detailed reconstruction of biogeographic scenarios during the Cretaceous.

Somewhat surprisingly, *Aquilops* is not closely related to later ceratopsians from North America: instead, phylogenetic and biogeographic data suggest a complex history of interchanges between North America and Asia (Figure 10). Given the age of *Aquilops* and its closest relatives, the ancestor of *Aquilops* dispersed into North America by the late Albian (presumably sometime before 104 Ma) and possibly as early as the Aptian (∼125 Ma, based on the date for the beds containing *Liaoceratops*; [84]). The ancestors of other North American ceratopsians may have entered North America at this time (implying extensive ghost lineages) or, more probably, later (discussed below). Although the DEC model cannot completely exclude alternative possibilities, we posit that the bulk of the evidence supports at least two dispersals in addition to that for *Aquilops*: one for leptoceratopsids and one for ceratopsoids. This is consistent with many previous models of ceratopsian biogeography [10], [12], [50], [63], [85], but adds some noteworthy details.

The timing of the dispersal of leptoceratopsids into North America is uncertain. The basalmost leptoceratopsid, *Asiaceratops*, is early Cenomanian in age (96.2–100.5 Ma), suggesting that the ancestor of North American leptoceratopsids was in North America sometime after that interval. The oldest leptoceratopsid known from North America, *Gryphoceratops*, is late Santonian in age (83.6–84 Ma). Given the poor record of early Late Cretaceous vertebrates in North America and the phylogenetic position of *Gryphoceratops* (deeply nested within the family; see [65]; see also Figure 10 herein), a pre-Santonian arrival in North America appears likely for Leptoceratopsidae. Interestingly, DEC reconstructs the ancestor of most leptoceratopsid clades as spanning both Asia and North America. This result should be regarded as extremely tentative, in part due to the lability of the reconstruction of leptoceratopsid relationships. Additionally, the ancestral ranges for nodes within leptoceratopsids seem unlikely based on the apparent separation of North America and Asia from the Turonian until the late Campanian or early Maastrichtian, as suggested by coastline reconstructions and biogeographic evidence (e.g., [81], [86]). Resolution of this issue requires additional analysis and basic data improvement, in the form of new fossils from the early Late Cretaceous.

Although relationships among leptoceratopsids are subject to debate, it is worth noting that two Asian taxa (*Udanoceratops* and *Zhuchengceratops*) are nested deeply within the group, suggesting the possibility of one or two dispersals from North America into Asia. The age of *Zhuchengceratops* is imprecisely known, beyond the observation that it comes from an Upper Cretaceous horizon [87]. *Udanoceratops*, from the Djadokhta Formation, is probably Campanian in age [88], perhaps 71–75 Ma [89], suggesting that dispersal (if any) occurred before or during that time interval. Branch support within leptoceratopsids is quite low, however, leaving open the possibility that Asian leptoceratopsids form an exclusive clade.

A biogeographic connection between North America and Asia before or during the Turonian is suggested by the occurrence of Turonian-aged Ceratopsoidea on both landmasses: *Zuniceratops* in New Mexico [72] and *Turanoceratops* in Uzbekistan [90]. Thus, DEC reconstructs the clade's common ancestor as spanning Asia and North America. This connection is in concordance with inferred low sea levels [91], [92] and paleogeographic reconstructions indicating subaerial causeways between the continents at these times [86]. Ceratopsidae are reconstructed by DEC as originating in North America, which is consistent with fossil evidence.

In sum, the phylogenetic and biogeographic evidence suggests that the immigration event(s) for *Aquilops* and the ancestors of other North American clades—including ceratopsoids and leptoceratopsids—began sometime within the interval spanning the Barremian and Albian (as indicated by the probable Barremian or early Aptian age of *Liaoceratops* [93], [94], a close relative of *Aquilops,* as well as neoceratopsian teeth from the early Albian Arundel Clay of Maryland [12], [83]. In the case of ceratopsoids, the dating of *Zuniceratops* as Turonian indicates that this clade entered North America no later than about 90 Ma. Ceratopsoids and leptoceratopsids may have entered North America simultaneously or separately, an issue that cannot be resolved with the patchy mid-Cretaceous fossil record.

Within the broader context of Cretaceous North American dinosaur biogeography, this new analysis is consistent with several previously proposed hypotheses. First, the occurrence of *Aquilops* in the late Albian of North America is congruent with the evidence from other clades for faunal interchange between North America and Asia in the latter part of the Early Cretaceous [9], [12], [13]. Second, the temporal and geographic distribution of other North American neoceratopsians (leptoceratopsids and ceratopsoids) suggests at least intermittent connections between North America and Asia up to and including the Turonian, likely followed by an interval of isolation and then reconnection, potentially during the late Campanian. This, too, is consistent with records for therizinosaurs [95], hadrosauroids [96], [97], tyrannosauroids [81], and other clades, as well as with many paleogeographic reconstructions [86].

Given the probable origin of Neoceratopsia in Asia and their appearance in North America by the late Early Cretaceous, how did the group disperse from one landmass to the other—directly through Beringia, or via a trans-European route? As has long been recognized, Late Jurassic and earliest Cretaceous vertebrate assemblages of the western USA share points of similarity with earliest Cretaceous faunas of Europe [18], [27], [98]–[102]. Chinnery-Allgeier and Kirkland [12] suggested that trans-European dispersal explains the presence of neoceratopsian teeth in the Arundel Clay of Maryland. This scenario is uncertain due to the imprecisely known age of the Arundel (though no data known to us place it anywhere near the Barremian–Aptian boundary, as suggested by Chinnery-Allgeier and Kirkland [12]; an early Albian age is far more likely [83]) and the incomplete nature of the fossils. The Arundel specimens are almost certainly neoceratopsian, based on their morphology, but placement beyond this (particularly relative to *Aquilops*) is uncertain. A European dispersal route from Asia is possible biogeographically, particularly in light of European occurrences of gobiconodontid and spalacolestine mammals during the Early Cretaceous [98], [103]–[105]. However, these groups (and others not known from Europe) also occur in Asia (see [106] and references therein; [107]–[109]), and Asia is also the likely source for lizard groups appearing by the Albian–Cenomanian boundary in North America [110]. Many other North American taxa (including dinosaurs) from the Aptian–Albian are not particularly closely allied with European species yet have plausible close Asian relatives (see summary in [13]). Addressing the point directly in the present context, no ceratopsians earlier than Santonian are known from Europe, and thus Europe doesn't figure in biogeographic reconstructions by the DEC model. Most critically, a denser sampling of Aptian and Albian vertebrates from Europe is necessary to establish the presence of ceratopsians there. This also does not exclude the possibility of occasional dispersals from Asia to North America via Europe as well as across Beringia. A trans-European model could be correct, but the biogeographic evidence at present is not particularly strong, as noted by Zanno and Makovicky [13], [18]. Furthermore, paleogeographic reconstructions do not strongly indicate a direct subaerial connection between western North America and Europe after the Barremian (e.g., [86], [111], but see [112] for a contrasting interpretation; summary in [12]). These reconstructions are hampered, in turn, by spotty preservation of appropriately aged rocks.

We believe that the evidence in hand shows the origin of Neoceratopsia in Asia and dispersal to North America by the Albian, but cannot conclusively speak to European vs. Beringian models. At present we favor the latter, largely on the admittedly negative evidence for earlier (Barremian or Aptian) presence of Neoceratopsia in North America and the absence of phylogenetically relevant materials from the European record. In order to better elucidate the timing and mode of these events, additional fieldwork in appropriate stratigraphic intervals for Asia, Europe and North America (especially in the Aptian–Santonian) will be most helpful.

## Supporting Information

File S1 **Supporting Information.** Including the following: 1) Text S1, character list used for phylogenetic analysis; 2) Text S2, codings for phylogenetic analysis, in TNT format; 3) Text S3, coding for phylogenetic analysis, in TNT format, with ontogeny-dependent characters of *Aquilops* scored as unknown; 4) Figure S1, most parsimonious tree, recovering *Ajkaceratops* as most basal neoceratopsian, with synapomorphy list; 5) Figure S2, most parsimonious tree, recovering *Ajkaceratops* as sister to *Bagaceratops*, with synapomorphy list; 6) Figure S3, bootstrap and Bremer support values for phylogenetic hypothesis; 7) Figure S4, strict consensus of 18 equally parsimonious trees, with selected ontogenetic-variant characters for *Aquilops americanus* scored assuming that the holotype is a juvenile; 8) Figure S5, strict consensus of 18 equally parsimonious trees, with selected ontogenetic-variant characters for *Aquilops americanus* scored assuming that the holotype is a juvenile (*Ajkaceratops* excluded). 9) Figure S6, phylogenetic tree recovering *Ajkaceratops* as sister to *Bagaceratops*, used for DEC modeling, with nodal numbers referenced in results (Table S1); 10) Table S1, temporal calibrations used for DEC analysis; 11) Figure S7, Summary figure of DEC modeling results; 12) Table S2, node-by-node results for DEC analysis; 13) Literature cited.
(PDF)

File S2 **Character matrix for phylogenetic analysis of Ceratopsia in TNT format.**
(TNT)

File S3 **Character matrix for phylogenetic analysis of Ceratopsia in TNT format, with ontogeny-dependent characters of** ***Aquilops*** **scored as unknown.**
(TNT)

File S4 **Files used for running biogeographic analysis of Ceratopsia.** Including the following: script.txt, R script for creating time calibrated trees; ceratopsian.nex, phylogenetic tree to which time calibration was applied; ceratopsian_ages.txt, ages (in Ma) for taxa; ranges.txt, geographic ranges for use in Lagrange.
(ZIP)

File S5 **Python script for biogeographic analysis in Lagrange 2.0, for phylogeny time-scaled using “aba” option.**
(PY) Click here for additional data file.

File S6 **Python script for biogeographic analysis in Lagrange 2.0, for phylogeny time-scaled using “equal” option.**
(PY) Click here for additional data file.

File S7 **Surface scans of elements from the lower jaw of** ***Aquilops americanus*** **, OMNH 34557 (holotype).**
(ZIP)

File S8 **Surface scan of the cranium of** ***Aquilops americanus*** **, OMNH 34557 (holotype).**
(ZIP)

File S9 **Color surface scan of the cranium of** ***Aquilops americanus*** **, OMNH 34557 (holotype).**
(ZIP)

File S10 **3D PDF of the lower jaw of** ***Aquilops americanus*** **, OMNH 34557 (holotype), based on scans from File S7.**
(PDF)

File S11 **Color 3D PDF of the cranium of** ***Aquilops americanus*** **, OMNH 34557 (holotype), based on scans from File S9.**
(PDF)

File S12 **3D PDF of the cranium of** ***Aquilops americanus*** **, OMNH 34557 (holotype), based on scans from File S8.**
(PDF)
