# Supplementary material for: A Ceratopsian Dinosaur from the Lower Cretaceous of Western North America, and the Biogeography of Neoceratopsia
Source: PLoS One. 2014 Dec 10;9(12):e112055. doi: 10.1371/journal.pone.0112055 (PMC4262212; doi:10.1371/journal.pone.0112055)
Supplement: File S1 — Supporting Information. Including the following: 1) Text S1, character list used for phylogenetic analysis; 2) Text S2, codings for phylogenetic analysis, in TNT format; 3) Text S3, coding for phylogenetic analysis, in TNT format, with ontogeny-dependent characters of Aquilops scored as unknown; 4) Figure S1, most parsimonious tree, recovering Ajkaceratops as most basal neoceratopsian, with synapomorphy list; 5) Figure S2, most parsimonious tree, recovering Ajkaceratops as sister to Bagaceratops, with synapomorphy list; 6) Figure S3, bootstrap and Bremer support values for phylogenetic hypothesis; 7) Figure S4, strict consensus of 18 equally parsimonious trees, with selected ontogenetic-variant characters for Aquilops americanus scored assuming that the holotype is a juvenile; 8) Figure S5, strict consensus of 18 equally parsimonious trees, with selected ontogenetic-variant characters for Aquilops americanus scored assuming that the holotype is a juvenile (Ajkaceratops excluded). 9) Figure S6, phylogenetic tree recovering Ajkaceratops as sister to Bagaceratops, used for DEC modeling, with nodal numbers referenced in results (Table S1); 10) Table S1, temporal calibrations used for DEC analysis; 11) Figure S7, Summary figure of DEC modeling results; 12) Table S2, node-by-node results for DEC analysis; 13) Literature cited. (PDF) [file pone.0112055.s001.pdf]

File S1. Supporting Information

A Ceratopsian Dinosaur from the Lower Cretaceous of Western North America, and the Biogeography of Neoceratopsia

Andrew A. Farke[1,3]*, W. Desmond Maxwell[2,3], Richard L. Cifelli[3], Mathew J. Wedel[4,3]

[1]Raymond M. Alf Museum of Paleontology, Claremont, California, United States of America; [2]Department of Biological Sciences, University of the Pacific, Stockton, California, United States of America; [3]Sam Noble Oklahoma Museum of Natural History, Norman, Oklahoma, United States of America; [4]College of Podiatric Medicine, Western University of Health Sciences, Pomona, California, United States of America

*Email: afarke@webb.org

Contents

Text S1. Character list used for phylogenetic analysis.
Text S2. Codings for phylogenetic analysis, in TNT format. See also File S2.
Text S3. Codings for phylogenetic analysis, in TNT format, with ontogeny-dependent characters of *Aquilops* scored as unknown. See also File S3.
Figure S1. Most parsimonious tree, recovering *Ajkaceratops* as most basal neoceratopsian, with synapomorphy list.
Figure S2. Most parsimonious tree, recovering *Ajkaceratops* as sister to *Bagaceratops*, with synapomorphy list.
Figure S3. Bootstrap and Bremer support values for phylogenetic hypothesis.
Figure S4. Strict consensus of 18 equally parsimonious trees, with selected ontogenetic-variant characters for *Aquilops americanus* scored as unknown, assuming that the holotype is a juvenile.
Figure S5. Strict consensus of 18 equally parsimonious trees, with selected ontogenetic-variant characters for *Aquilops americanus* scored as unknown, assuming that the holotype is a juvenile. In this version of the tree, the wildcard taxon *Ajkaceratops* has been pruned *post hoc*.
Figure S6. Phylogenetic tree recovering *Ajkaceratops* as sister to *Bagaceratops*, used for DEC modeling, with nodal numbers referenced in results (Table S1).
Table S1. Temporal calibrations used for DEC analysis.
Figure S7. Summary figure of DEC modeling results.
Table S2. Node-by-node results for DEC analysis.
Literature Cited

Text S1. Character list used for phylogenetic analyis. Characters 1–133 are taken directly from Makovicky and Norell (2006; with 140 modified following character 134 of Lee et al. 2011), characters 134–147 from Makovicky (2010), characters 148 and 149 from Lee et al. (2011; corresponding to their characters 135 and 136); characters 150 and 151 from Ryan et al. (2012). The full character list is provided here as a convenience to the reader; please see the main text and referenced publications for additional explanations and the history of usage for each character.

1. Head size small relative to body (0) or large relative to body (1).
2. Head shape in dorsal view: elongate, ovoid (0), or triangular, wide over jugals (1).
3. Orbit diameter more than 20% of skull length (0) or less (1).
4. Preorbital region more than 40% (0) or less than 40% (1) the length of the skull.
5. Tip of rostral low and level with maxillary tooth row (1) or raised and dorsal to maxillary tooth row (1).
6. Rostral bone forming beak absent (0) or present (1).
7. Rostral ventral (buccal) process absent (0) or present (1).
8. Anterior face of rostral round, convex (0) or sharply keeled (1).
9. Premaxillary palatal region flat in ventral view (0) or vaulted dorsally (1).
10. Relative height of premaxilla (snout) to orbital region low (0) or deep (1).
11. Premaxilla–prefrontal contact absent (0) or present (1).
12. Convex buccal process anterior to maxillary tooth row formed by premaxilla or premaxilla and maxilla absent (0) or present (1).
13. Premaxilla–maxilla buccal margin relatively straight in ventral view, tooth rows/buccal margins converge rostrally (0) or buccal margin sinuous in ventral view, with premaxillary palatal region flaring widely rostral to tooth row (1).
14. Anterior end of the nasal (internarial bar) above (0) or below and far rostral to the external naris (1).
15. Nares position close to buccal margin (0) or dorsal, away from buccal margin (1) or very far dorsal, level with upper part of orbit (2).
16. Ventral border of external nares significantly below (0), about the level of (1), or significantly above (2) lower rim of infratemporal fenestra.
17. Large depression excavating premaxilla anteroventral to naris absent (0) or present (1).
18. Nasal horn absent (0), small (1), or large (2).
19. Naris width (excluding narial depression) less than 10% of skull length (0) or more than 10% of skull length (1).
20. Position of choana on palate: anterior to maxillary tooth row (0) or level with maxillary tooth row (1).
21. Maxillae from opposite sides separated by vomers at anterior border of the internal choanae (0) or maxillae contact each other anterior to choanae in palatal view and tip of vomer obscured from view (1).
22. Dentigerous margin of maxilla straight (0) or ventrally convex (1).
23. Antorbital fossa reduced or absent (0) or large and triangular or rounded in shape (1).
24. Eminence or tubercle on the rim of the buccal emargination of the maxilla near the junction with the jugal absent (0) or present (1).
25. Palpebral free, articulating with lacrimal (0) or fused to orbital margin (1).
26. Jugal–lacrimal contact reduced (0) or expanded (1).
27. Jugal horns absent (0) or present and laterally directed (1) or present and ventrally directed (2).
28. Jugal suborbital ramus not as deep as subtemporal ramus (0) or suborbital ramus as deep or deeper than orbital ramus (1).
29. Epijugal ossification absent (0) or present (1).

30. Epijugal position on jugal: along dorsal edge of horn (epijugal trapezoidal) (0) or capping end of horn (epijugal conical) (1).
31. Orbital horns absent (0) or present (1).
32. Postorbital inverted L-shaped (0) or triangular and platelike (1).
33. Postorbital with dorsal part rounded and overhanging lateral edge of supratemporal fenestra (0) or with concave dorsal shelf bordering supratemporal fenestra (1).
34. Laterotemporal fenestra with postorbital participation in margin (0), postorbital excluded from margin (1), or jugal–squamosal contact very wide and postorbital situated far from fenestra (2).
35. Laterotemporal fenestra width more than 10% of skull length (0) or less than 10% of skull length (1).
36. Squamosal subtriangular in lateral view (0) or T-shaped, with postquadratic process (1).
37. Temporal process of squamosal simple (0) or deeply bifurcate around temporal process of postorbital (1).
38. Posterior edge of squamosal angled anteromedially (0) or posteromedially, squamosal contributing lateral portion of frill margin (1).
39. Temporal bars of squamosals parallel (0) or posteriorly divergent (1).
40. Quadratojugal mediolaterally flattened (0) or transversely expanded and triangular in coronal section (1) or triangular in coronal section, but with slender anterior prong articulating with jugal (2).
41. Quadrate shaft anteriorly convex in lateral view (0) or straight (1).
42. Elongate parasagittal process of the palatine absent (0) or present (1).
43. Ectopterygoid exposed in palatal view (0) or reduced and concealed in palatal view (1).
44. Ectopterygoid contacts jugal (0) or ectopterygoid reduced and restricted to contact with maxilla (1).
45. Pterygopalatine foramen (modified suborbital fenestra) large (0) or diminutive (1).
46. Ventral ridge on mandibular process of pterygoid defining "Eustachian canal" absent (0) or present (1).
47. Pterygoid–maxilla contact at posterior end of tooth row absent (0) or present (1).
48. Prominent posterior midline process on pterygoid absent (0) or present (1).
49. Pterygoid mandibular process short (0) or long, extending well below maxillary tooth row (1).
50. Pterygoid mandibular process formed only by pterygoid (0) or jointly by pterygoid and ectopterygoid (1).
51. Parieto-frontal contact flat (0), depressed (1), or invaginated by fontanelle (2).
52. Parieto-squamosal frill absent (0) or parietal frill less than 70% of basal length of skull (1) or more than 70% of basal length (2).
53. Dorsal edge of squamosal temporal bar curves medially at the posterior end, arcing confluently into posterior frill margin (0) or dorsal edge of squamosal meets posterior margin of frill at acute angle (1).
54. Frill solid (0) or fenestrated near posterior margin (1).
55. Distinctive indentation on midline of the posterior parietals present (0) or absent (1).
56. Epoccipital ossifications/frill scallops absent (0) or present (1).
57. Basioccipital participates in foramen magnum (0) or basioccipital is excluded from foramen magnum and exoccipitals form less than onethird of condyle (1) or exoccipitals form about half or more of occipital condyle (2).
58. Basioccipital excluded from basal tubera by basisphenoid and limited to occipital midline (0) or basioccipital tubera present (1).
59. Basipterygoid process orientation anterolateral (0), ventral (1), or posteroventral (2) when braincase is oriented with condyle pointing posteriorly.
60. Basioccipital tubera flat, in plane with basioccipital plate (0) or everted posterolaterally,

forming lip beneath occipital condyle (1).

61. Notch between posteroventral edge of basisphenoid and base of basipterygoid process deep (0) or notch shallow and base of basipterygoid process close to basioccipital tubera (1).
62. Exoccipital with three exits for cranial nerves X–XII near occipital condyle (0) or with two exits (1).
63. Exoccipital–quadrate separated by ventral flange of squamosal (0) or in contact (1).
64. Paroccipital processes deep (height $ K length) (0) or significantly narrower (1).
65. Supraoccipital participates in dorsal margin of foramen magnum (0) or excluded from foramen magnum by exoccipitals (1).
66. Supraoccipital anteriorly inclined relative to basioccipital (0) or in same plane as posterior face of basioccipital (1).
67. Supraoccipital shape tall, triangular (0) or wider than tall, trapezoid (1) or square (2).
68. Tip of predentary shallow (0) or scooplike (1).
69. Predentary with rounded anterior margin and distally broad posteroventral process (0) or with pointed anterior margin and distally narrow posteroventral process (1).
70. Predentary less than two-thirds of dentary length (0) or equal to or more than two-thirds of dentary length (1).
71. Predentary buccal margin sharp (0) or with a rounded, beveled edge (1) or with grooved, triturating edge (2).
72. Tip of dentary smooth (0) or grooved dorsally for reception of the lateral process of the predentary (1) or bears large pit for reception of the lateral process of the predentary (2).
73. Dentary symphyseal area small (0) or large, forming strong immobile bond with participation of splenial (1).
74. Diastema between predentary and first dentary tooth absent (0) or present (1).
75. Ventral margin of dentary curved (0) or straight (1) in lateral view.
76. Dentary flange absent (0) or present along ventral edge (1).
77. Prominent medial expansion of the central mandible in the middle of the tooth row formed by wide Meckelian groove separating tooth-bearing part of the jaw from external surface absent (0) or present (1).
78. Labial face of dentary smooth below tooth row (0) or rugose and sculpted (1).
79. Contact between dentary and prearticular absent (0) or present (1).
80. Posterior end of splenial simple or with shallow dent (0) or with bifid overlap of angular (1).
81. Distal end of coronoid process rounded (0) or with anterior expansion (1).
82. Coronoid process positioned close to main axis of dentary and posterior to tooth row (0) or set lateral to tooth row, and end of tooth row covered by anterior part of coronoid process (1) or tooth row level with posterior edge of coronoid process (2).
83. Coronoid straplike and with subequal depth throughout (0) or with lobate, highly expanded dorsal end much deeper than ventral end that slots between splenial and dentary (1).
84. Surangular without distinct lateral ridge or shelf overhanging angular (0) or shelf/ridge present (1).
85. Lateral surface of surangular flat or only weakly convex (0) or with pronounced laterally convex curvature (in the transverse plane) between the coronoid process and glenoid region (1).
86. Tab on surangular forming lateral wall to glenoid cotyle absent (0) or present (1).
87. Angular without one or more small, lateral tubercles along ventral rim below glenoid articulation (0) or tubercles present (1).
88. Lateral surface of angular flat or slightly convex (0) or angular bears a raised emargination along posteroventral margin of mandible, lateral surface distinctly concave (1).
89. Angular–surangular–dentary contact triradiate (0) or surangular with long ventral process overlapping angular, dentary–surangular and angular–surangular sutures form acute angle on

lateral face of mandible (1).

90. Mandibular glenoid narrow and flush with medial margin of surangular flange in dorsal view (0) or glenoid region medially expanded and forming lingual process in dorsal view (1).
91. Surface of prearticular and articular below glenoid smooth (0) or with wide, semicircular ventral process near medial face of glenoid (1).
92. Retroarticular process long (0) or short or absent (1).
93. Three or more teeth in premaxilla (0) or two teeth in premaxilla (1) or 1 tooth in premaxilla (2) or premaxilla edentulous (3).
94. Premaxillary teeth with carinae, and in some cases serrations (0) or premaxillary teeth peglike, crown without carinae (1).
95. Teeth with single roots (0) or with double roots (1).
96. Cheek teeth spaced (0) or closely apressed with determinate eruption and replacement pattern (1).
97. Teeth occlude at an oblique angle (0) or at a vertical angle (1) or at a vertical angle, but dentary teeth have a horizontal shelf on the labial face (2).
98. Teeth without distinct median primary ridge (0) or with very weak and wide median ridge on at least some maxillary teeth (1) or all maxillary and dentary teeth with distinct primary ridge (2).
99. Base of primary ridge confluent with the cingulum on maxillary teeth (0) or base of primary ridge set back from cingulum, which forms a continuous ridge at the crown base (1).
100. Pronounced cingula on cheek teeth absent (0) or present (1).
101. Tooth row double, with only one replacement tooth present at a time (0) or battery-like with multiple ($\geq$3) rows of replacement teeth (1).
102. Both lingual and buccal sides of teeth covered with enamel (0) or enamel restricted to lateral side of maxillary and medial side of dentary teeth (1).
103. Dentary tooth crowns with continuous, smooth root crown transition (0) or bulbous expansion at root-crown transition on labial side of tooth, sometimes worn to form notch or shelf (1).
104. Number of alveoli in dentary less than 20 (0) or more than 20 (1).
105. Cheek teeth with cylindrical roots (0) or roots with mesial and distal faces flattened to slightly grooved (1).
106. Tooth crowns radiate or pennate in lateral view (0) or crowns ovate in lateral view (1).
107. Atlas intercentrum semicircular (0) or disc shaped (1).
108. Atlas intercentrum not fused to odontoid (0) or fused (1).
109. Atlas neurapophyses free (0) or fused to intercentrum/odontoid (1).
110. Axial neural spine low (0) or tall and hatchetshaped (1) or elongate and posteriorly inclined (2).
111. The neural spine of the axis anteroposteriorly short (0) or long, extending caudally to the posterior end of the centrum of the succeeding cervical (1).
112. Syncervical absent (0), partially fused (centra but not arches) (1), or completely coossified (2).
113. Dorsal vertebrae with flat articulations on zygapophyses (0) or tongue and grooves articulations on zygapophyses (1).
114. Number of sacrals: five (0) or six (1) or seven (2) or eight or more (3).
115. Outline of sacrum defines rectangle or hourglass in dorsal view (0) or oval in dorsal view (1).
116. Caudal neural spines short and inclined (0) or tall and straight (1).
117. Tail terminates with series of cylindrical caudals that are devoid of neural spines and chevrons (0) or neural spines and chevrons persist virtually to the end of tail (1).
118. Distal chevrons with lobate expanded shape (0) or rodlike (1).
119. Clavicles absent (0) or present (1).
120. Scapula distinctly curved in sagittal view (0) or relatively flat (1).
121. Scapular blade at acute angle relative to glenoid (0) or almost perpendicular to glenoid (1).
122. Coracoid with smooth, arcuate anterior portion (0) or bearing large anterolateral ridge near

confluence of anterior and ventral margins (1).

123. Olecranon process relatively small (0) or enlarged (≥1/3 of ulnar length) (1).
124. More than two distal carpals (0) or less than two distal carpals (1).
125. Manus much smaller than pes (0) or closer to pes in size (1).
126. Shaft of postpubis round (0) or mediolaterally flattened, bladelike (1) in cross section.
127. Postpubis long and ventrally oriented (0) or short and posteriorly directed (1).
128. Prepubis short and rod-shaped (0) or long and flared at anterior end (1).
129. Ischial shaft straight (0) or with posterodorsally convex curvature (1).
130. Femoral fourth trochanter large and pendant (0) or reduced (1).
131. Tibio-femoral ratio more than one (0) or less than one (1).
132. Foot gracile with long, constricted metatarsus, elongate phalanges (0) or short and uncompressed, all phalanges wider than long (1).
133. Pedal unguals pointed (0) or rounded, hooflike (1).
134. Postorbital lateral surface smooth (0) or strongly sculptured (1).
135. Angular lateral surface smooth (0) or strongly sculptured (1).
136. Rostral end of quadratojugal contacting jugal undivided (0) or bifid around caudal end of jugal (1).
137. Nasals flat or convex on midline (0) or with distinct nasal midline depression (1).
138. Parietal roof flat or gently convex (0) or with sharp midline crest (1).
139. Surangular without a lateral process below glenoid (0) or surangular knob (1).
140. Height of middle caudal neural spine less than or equal to 2 (0), 2.1 to 3 times (1), 3.1 to 4 times (2) or more than 4.1 times (3) the height of the associated centrum.
141. Ridge along the caudoventral edge of squamosal absent (0) or present (1).
142. Occipital surface of supraoccipital flat, convex, or with midline ridge (0) or with midline depression along base of midline ridge (1).
143. Accessory antorbital fenestra between naris and antorbital fenestra absent (0) or present (1).
144. Radius without lateral and medial tuberosities along distal half of shaft (0) or tuberosities present (1).
145. Coronoid process notch along caudal edge of dentary coronoid process absent (0) or present (1).
146. Coronoid process notch wide (0) or constricted notch (1).
147. Cervical centra with ventral keels (0) or some or all postaxial centra without keels (1).
148. Width of proximal end of ungual relative to width of distal end of the preceding phalanx equal (0) or wider (1).
149. Height of the caudal neural spine equal to or less than the length of the corresponding chevron (0) or greater (1).
150. Anteroventral chin absent (0), poorly developed (1) or well-developed (2).
151. Base (lower half) of the coronoid process vertical (0) or steeply inclined (>40%).
152. Circumnarial depression, if deep, simple (0) or complex (1).
153. Narial spine absent (0) or present (1).

Text S2. Codings for phylogenetic analysis, in TNT format. Results are presented in Figures S1, S2, and S3. See also File S2.

```
xread
153 33
Hypsilophodon  00000???00000000000000001000000-00000000000000?0001100--
     00000001?
     1000010000010001000000000000000000000000000000000000000000000000
     000000000000000000-00000-0
Stegoceras     00000???0000000000000000011000-0??010010?000000011100--
     0000000?00000?00?000100100000000?0000000000000000000??0?0?
     10????00?0?0???0100011?-0?0?0000-?0000-0
Asiaceratops   ????1???1?????1?0????
     01?????????????????????????????????11?00?????????1101??0???????
     01??????3-000?000?001??????????1????1???????????0??0??
     0??????????????
Bagaceratops   11001111101?01?01?0101000111101010101121100?111????
     1110111010010011111111110?01101111?111013-000201010011????
     1??????????????0??????000010300 1?0-?1100-0
Chaoyangsaurus 110111001??00?120????0?1??010?????0????0????
     00001????????01?0????????1100?101001??00000000?
     11110000000000000000000?????????????????????110??0?????0-0??00-?
Centrosaurus
          11101111110110011211100011211111021101111211111100221 1?
     1211011101121102111 10?01012101100 0?013-
     1112011101111112120310111010111111111111000000000010-1100001
Leptoceratops  11001111101101200001110001110011000110110 0??111?
     10100001 1?100110101111210001010011011010101013-
     0022110110110101110101110110000000000000000001000001100011 0-0
Graciliceratops     1100?????????????????0??????1????????
     101111??????????????100?1??????????????????1?1??0??01?111???1????
     000201010 0?????????1??1???1??????0??0000000?10?00000-?1?0?-?
Montanaceratops     1????????1????1????010100?1?10011??011?
     11?????????0?0???112100?1010?1???1?101010011111 0??1013?
     0022110110?10111?11201110110?0000000000?00?10211??1100100-?
Protoceratops  110011111010012010010100011110101010101121100111 11?
     1211[0
     1]011101001001111111100101101111101110111000201010011011111112011
     10110000100000010 00?10200000-11100-0
Psittaco_mongol     1101110011101122000110010011 0?
     0000000000001000111101001001000001001000011 0-1000?01?000000100 3-
     0000000000000000100000000111000000000000001011000000-00000-0
Triceratops

     1110111111001001121110001121111102110111121111110022101121101110
     112110211110?0101210110001013-1112011101 1?
     111212031011101011111111111100000000000 10-1100010
Udanoceratops  1??0111111?0??1?00?0?1????1110?????????
     11?????????????????????????????1??21?0??0?001?011??01013-
```

002211011011??????02011??1100??????0????000101??01???1100-0
Zuniceratops ???????1????????10???0????1???1???????????????????
1??????1?????????????211?1??0??0????????????-01120101?
01???????????0????????????1????0?00??0?01?0-???0000
Psittaco_sinen 110111001110112200 0??00000110-0000000000?00????1?
010010???????0???0000110100 0??01?000000??03-00?0?000?000??0010?
1000???10??????0?000010110 0?000-?0000-0
Xuanhuaceratops ????????????
0??
11010?1?????0?000?1111?000000000000000????0??????110???????0?0?
110?????????-0??0???
Yamaceratops 11??1110????1??????
01010001010010100110111000001110 10??111010?100111?11101010?
001111110?101[0 1]00001000100 11??????0??1???????????????00000?
10?00??0-0??00-0
Prenoceratops ??0?1???1?00101?000??0100?1110011??011?11???????????
0???11210??????11?12100010??011011???1013-0022110110?
1???????????????????????????0?00?0000001000110-0
Cerasinops 1????????????????????0????10??01???011?11?????????
110100111100?1010?????1?0??????0[1 2]1111???1?11?00210?0?1????0?
1101[1 2]?1?1?1100?????1000000??10?00??0-00?01-0
Yinlong 01001100?0?0?001000??00001000-01000000000000?00?1?00--
1100200??00?10100100000 0?0000000101?10000000000000?0000??0??00?
0?????00100?0??0110111?000?0-???00-0
Koreaceratops ???
??
101??????????0??00???????3????????01????
Zhuchengceratops 1????????????????????1???1??????????????????
111111??????????????????10??11000010111000 01?101???
0221101101 1???010??????????????????????????0???0?????101??00??
Liaoceratops ?100111111101011000010110010 0-
010100110011000001110101100110 10?10011?0011001100001?
0101001010000010 0?10001??????????????????0??????????????000110?000?
0-???00-0
Aquilops ?1001111?1?1101?000??01000110-0101?????????????????
0?????????????????11?1110??10????????????????00000101010?
01?????????????????????????????0??0????00???????0?-?
Archaeoceratops 1100111111011011000??0100011100 1?110??01110?00?
11?010???11101?010011101?101010??01?11100?1010000010 1?000?1000??
0100?0????????????00??00000110000 0?0-00000-0
Auroraceratops ?1001???1101101100 0??010?01110010?00?101110?00?11?
0101??11101?0100?1102?101010000 1?11110010100?00101?100?
1???????????????????????????000?10?000?0-???00-0
Unescoceratops ??
?????????????????2100000??11??????????????00?2?10110?
1??21??
Gryphoceratops ??
?????????????????????????000??????????????????????00?????0??
0??1??

```
Ajkaceratops   ?????1111????01?0???
     1???????????????????????????????????????????????1?
     0???????????????????????????????????????????????????????????????
     ????????1????????-0
Diabloceratops 1110111111011001121??
     00011211111021101111211111100221111?
     1101110112???????????????????????013-111201?1???
     1???????????????????????????0?000??001??-?????01
Helioceratops  ??????????????????????????????????????????????????????
     ?????????????????1101010???1?????????????00101?000?
     1??????????????????????????????????????0-???00-?
Turanoceratops ?????????????????????0????????11??????????????????
     1????????????????1??2????????????????????????1121111??
     11???????????????????????????????????????????????
Chasmosaurus

     1110111111001001121110001121111102110111121111110022111121101110
     112110211110?01012101100010 13-11120111011?
     1112120310111010111111111110000000000010-1100010
;


proc /;
comments 0
;
```

Text S3. Codings for phylogenetic analysis, in TNT format, with ontogeny-dependent characters of *Aquilops* scored as unknown. Results are presented in Figure S4 and S5. See also File S3.

```
xread
153 33
Hypsilophodon  00000???000000000000000001000000-00000000000000?0001100--
     00000001?
     1000010000010001000000000000000000000000000000000000000000000000000
     000000000000000000-00000-0
Stegoceras     00000???000000000000000000011000-0??010010?000000011100--
     0000000?00000?00?00010010000000?00?0000000000000000000??0?0?
     10?????00?0?0???0100011?-0?0?0000-?0000-0
Asiaceratops   ????1???1?????1?0????
     01????????????????????????????????????11?00??????????1101??0??????
     01??????3-000?000?001??????????1??1????1???????????0??0??
     0??????????????
Bagaceratops   110011111101?01?01?0101000111101010101121100?111????
     1110111010010011111111110?011011111?111013-000201010011????
     1??????????????0??????0000103001?0-?1100-0
Chaoyangsaurus 110111001??00?120????0?1??010?????0????0????
     00001????????01?0????????1100?101001??00000000?
     1111000000000000000000?????????????????????110??0?????0-0??00-?
Centrosaurus
          1110111111011001121110001121111102110111121111110022 11?
     121101110112110211110?0101210110 00?013-
     1112011101111112120310111010111111111110000000000010-1100001
Leptoceratops  1100111110110120000111000111001100011011 00??111?
     10100001 1?1001101011112100010100110110101013-
     0022110110110101110101110110000000000000000010000011000110-0
Graciliceratops     1100???????????????0??????1????????
     101111??????????????100?1????????????????1?1??0??01?111???1????
     000201010 0????????1??1???1??????0??0000000?10?00000-?1?0?-?
Montanaceratops     1????????1????1????010100?1?10011??011?
     11?????????0?0???112100?1010?1???1?1010100111110??1013?
     0022110110?10111?1120110110?0000000000?00?10211??1100100-?
Protoceratops  110011111101001201001010001111010101010112110011111?
     1211[0
     1]0111010010011111111100101101111101110111000201010011011111112011
     101100001000000100 0?10200000-11100-0
Psittaco_mongol     1101110011101122000110010011 0?
     00000000000010001111010010010000010010000110-1000?01?0000001003-
     000000000000000001000000000111000000000000000101100000 0-00000-0
Triceratops

     111011111100100112111000112111110211011112111111002210112110111 0
     1121102111 10?0101210110001013-1112011101 1?
     111212031011101011111111111000000000010-1100010
Udanoceratops  1??0111111?0??1?00?0?1????1110?????????
     11????????????????????????????1??21?0??0?001?011??01013-
```

002211011011??????02011??1100??????0????000101??01???1100-0
Zuniceratops ???????1????????10???0????1???1???????????????????
1??????1????????????211?1??0??0????????????-01120101?
01??????????0????????????1????0?00??0?01?0-???0000
Psittaco_sinen 11011100111011220000??00000110-00000000000?00????1?
010010???????0???0000110100 0??01?000000??03-00?0?000?000??0010?
1000????10??????0?000010 1100?000-?0000-0
Xuanhuaceratops ????????????
0??
11010?1?????0?000?1111?00000000000000????0??????110???????0?0?
110?????????-0??0???
Yamaceratops 11??1110????1??????
0101000101001010011011100000111010??111010?100111?11101010?
001111110?101[0 1]0000100010011??????0??1???????????????00000?
10?00??0-0??00-0
Prenoceratops ??0?1???1?00101?000??0100?1110011??011?11???????????
0???11210??????11?12100010??011011???1013-0022110110?
1????????????????????????????0?00?0000001000110-0
Cerasinops 1????????????????????0????10??01???011?11?????????
110100111100?1010?????1?0?????0[1 2]1111???1?11?00210?0?1????0?
1101[1 2]?1?1?1100?????1000000??10?00??0-00?01-0
Yinlong 01001100?0?0?001000??00001000-0100000000000?00?1?00--
1100200??00?10100100000 0?00000000101?1000000000000?0000??0??00?
0?????00100?0??0110111?000?0-???00-0
Koreaceratops ??
??
101?????????0??00??????3???????01????
Zhuchengceratops 1????????????????????1???1?????????????????
111111????????????????????10??1100001011100001?101???
02211011011???010?????????????????????0???0?????101??00??
Liaoceratops ?100111111010110000101100100-
010100110011000001110101100110 10?10011?0011001100001?
01010010100000100?10001????????????????0????????????000110?000?
0-???00-0
Aquilops ?1001111?1?1101?000??0100011??0101????????????????
0????????????????11?1110??10??????????????00000?01010?
01???????????????????????????0??0???00??????0?-?
Archaeoceratops 11001111110110110000??0100011100 1?110??01110?00?
11?010???11101?010011101?101010??01?11100?10100000101?000?1000??
0100?0?????????00??0000011000 00?0-00000-0
Auroraceratops ?1001???11011011000??010?01110010?00?101110?00?11?
0101??11101?0100?1102?1010100001?11110010100?00101?100?
1????????????????????????????000?10?000?0-???00-0
Unescoceratops ??
?????????????????2100000??11????????????00?2?10110?
1???21??
Gryphoceratops ??
?????????????????????000????????????????00????0??
0??1??

```
Ajkaceratops   ?????1111????01?0???
     1???????????????????????????????????????????????1?
     0???????????????????????????????????????????????????????????????
     ????????1????????-0
Diabloceratops 1110111111011001121??
     00011211111021101111211111100221111?
     1101110112???????????????????????013-111201?1???
     1???????????????????????????0?000??001??-?????01
Helioceratops  ??????????????????????????????????????????????????????
     ?????????????????1101010???1?????????????00101?000?
     1??????????????????????????????????????0-???00-?
Turanoceratops ?????????????????????0????????11??????????????????
     1????????????????1??2????????????????????????1112111??
     11???????????????????????????????????????????????
Chasmosaurus

     1110111111001001121110001121111102110111121111110022111121101110
     112110211110?0101210110001013-11120111011?
     11121203101110101111111111100000000001 0-1100010
;


proc /;
comments 0
;
```

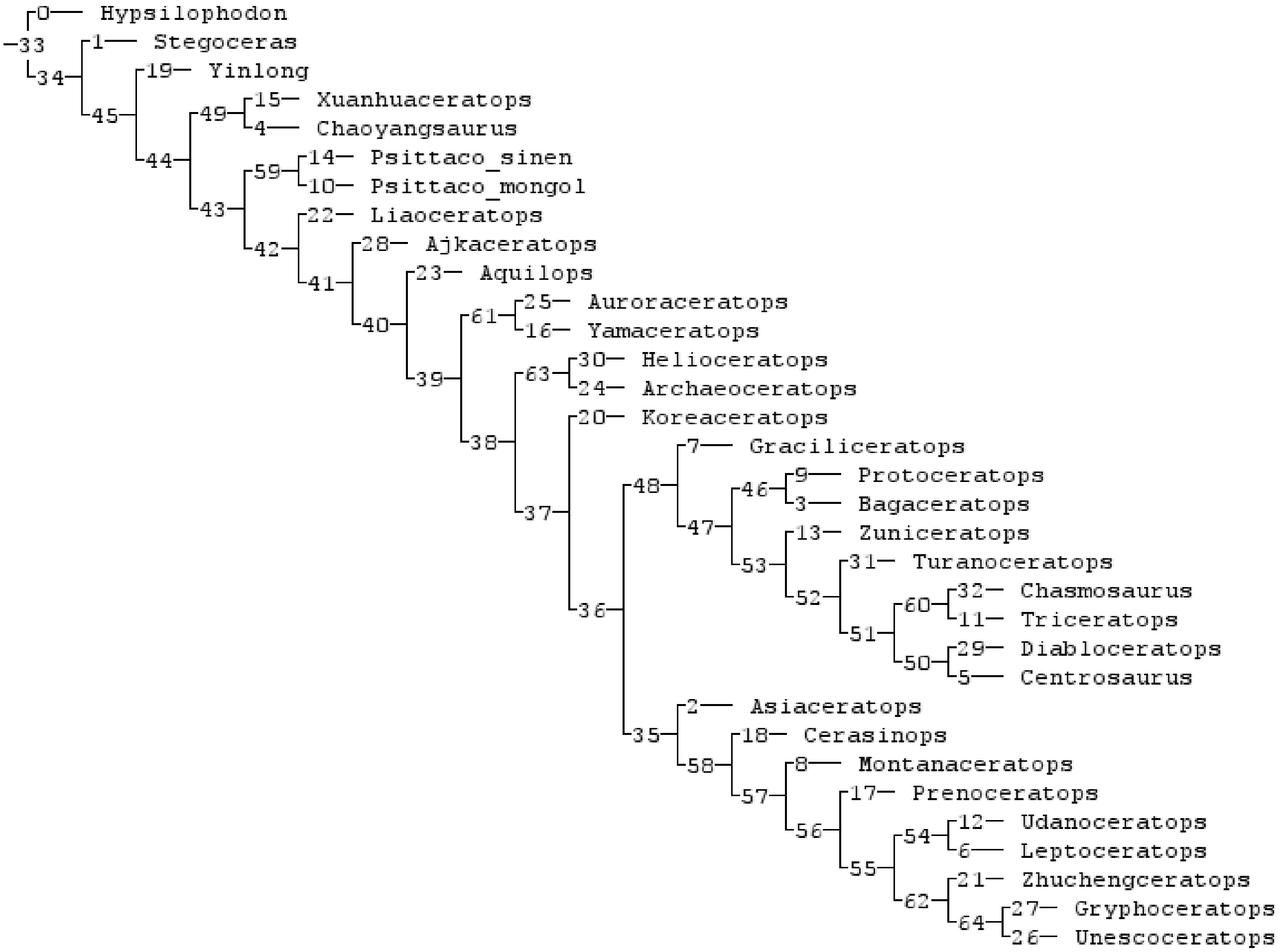

Figure S1. Most parsimonious tree, recovering *Ajkaceratops* as a basal neoceratopsian, with synapomorphy list.

Node 34: No synapomorphies
Node 35 (Leptoceratopsidae): 61(0)
Node 36: 118(1)
Node 37: 117(1), 149(1)
Node 38: 113(1)
Node 39: 29(1), 105(1)
Node 40: 71(1)
Node 41: No synapomorphies
Node 42 (Neoceratopsia): 7(1), 8(1), 23(1), 34(1), 37(1), 38(1), 41(1), 42(1), 59(1), 61(1), 77(1), 85(1), 98(1), 102(1), 106(1)
Node 43: 13(1), 27(1), 82(1), 111(1), 134(0), 135(0)
Node 44: 1(1), 15(1), 28(1), 58(1), 73(1)
Node 45 (Ceratopsia): 2(1), 5(1), 55(1), 67(1), 72(1), 90(1), 92(1), 138(1)
Node 46 (Protoceratopsidae): 18(1), 40(2), 70(1), 80(1), 89(1)
Node 47: 133(1)
Node 48 (Coronosauria): 36(1), 37(0), 39(1), 148(1)
Node 49: 78(1), 91(1), 93(1)
Node 50: 153(1)
Node 51 (Ceratopsidae): 51(2)
Node 52: 95(1), 101(1)
Node 53 (Ceratopsoidea): 17(1), 31(1), 71(2), 96(1), 97(1), 116(0), 129(1)
Node 54: No synapomorphies
Node 55: 22(1)
Node 56: 84(0)
Node 57: 59(2), 99(1), 145(1)
Node 58: 60(1), 97(2), 103(1)
Node 59 (*Psittacosaurus*): 14(1), 15(2), 69(0), 92(0), 93(3), 122(1), 136(1), 139(1)
Node 60: 12(0), 152(1)
Node 61: No synapomorphies
Node 62: 77(0), 81(1)
Node 63: 102(0)
Node 64: 151(1)

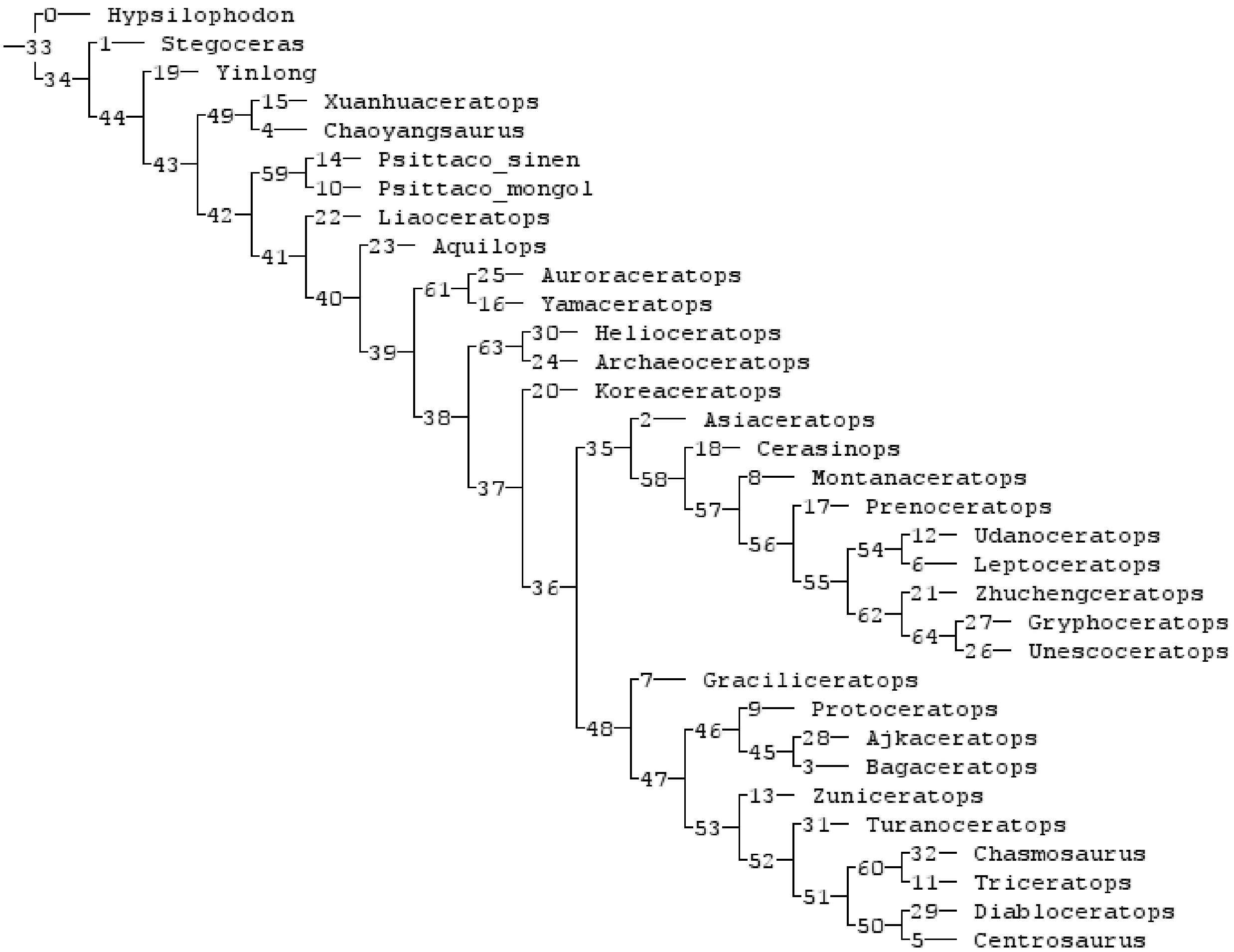

Figure S2. Most parsimonious tree, recovering *Ajkaceratops* as sister to *Bagaceratops*, with synapomorphy list.

Node 34: No synapomorphies
Node 35 (Leptoceratopsidae): 61(0)
Node 36: 118(1)
Node 37: 117(1), 149(1)
Node 38: 113(1)
Node 39: 29(1), 105(1)
Node 40: 12(1), 71(1), 100(1)
Node 41 (Neoceratopsia): 7(1), 8(1), 23(1), 34(1), 37(1), 38(1), 41(1), 42(1), 59(1), 61(1), 77(1), 85(1), 98(1), 102(1), 106(1)
Node 42: 13(1), 27(1), 82(1), 111(1), 134(0), 135(0)
Node 43: 1(1), 15(1), 28(1), 58(1), 73(1)
Node 44 (Ceratopsia): 2(1), 5(1), 55(1), 67(1), 72(1), 90(1), 92(1), 138(1)
Node 45: 143(1)
Node 46 (Protoceratopsidae): 18(1), 40(2), 70(1), 80(1), 89(1)
Node 47: 133(1)
Node 48 (Coronosauria): 36(1), 37(0), 39(1), 148(1)
Node 49: 78(1), 91(1), 93(1)
Node 50: 153(1)
Node 51 (Ceratopsidae): 51(2)
Node 52: 95(1), 101(1)
Node 53 (Ceratopsoidea): 17(1), 31(1), 71(2), 96(1), 97(1), 116(0), 129(1)
Node 54: No synapomorphies
Node 55: 22(1)
Node 56: 84(0)
Node 57: 59(2), 99(1), 145(1)
Node 58: 60(1), 97(2), 103(1)
Node 59 (*Psittacosaurus*): 14(1), 15(2), 69(0), 92(0), 93(3), 122(1), 136(1), 139(1)
Node 60: 12(0), 152(1)
Node 61: No synapomorphies
Node 62: 77(0), 81(1)
Node 63: 102(0)
Node 64: 151(1)

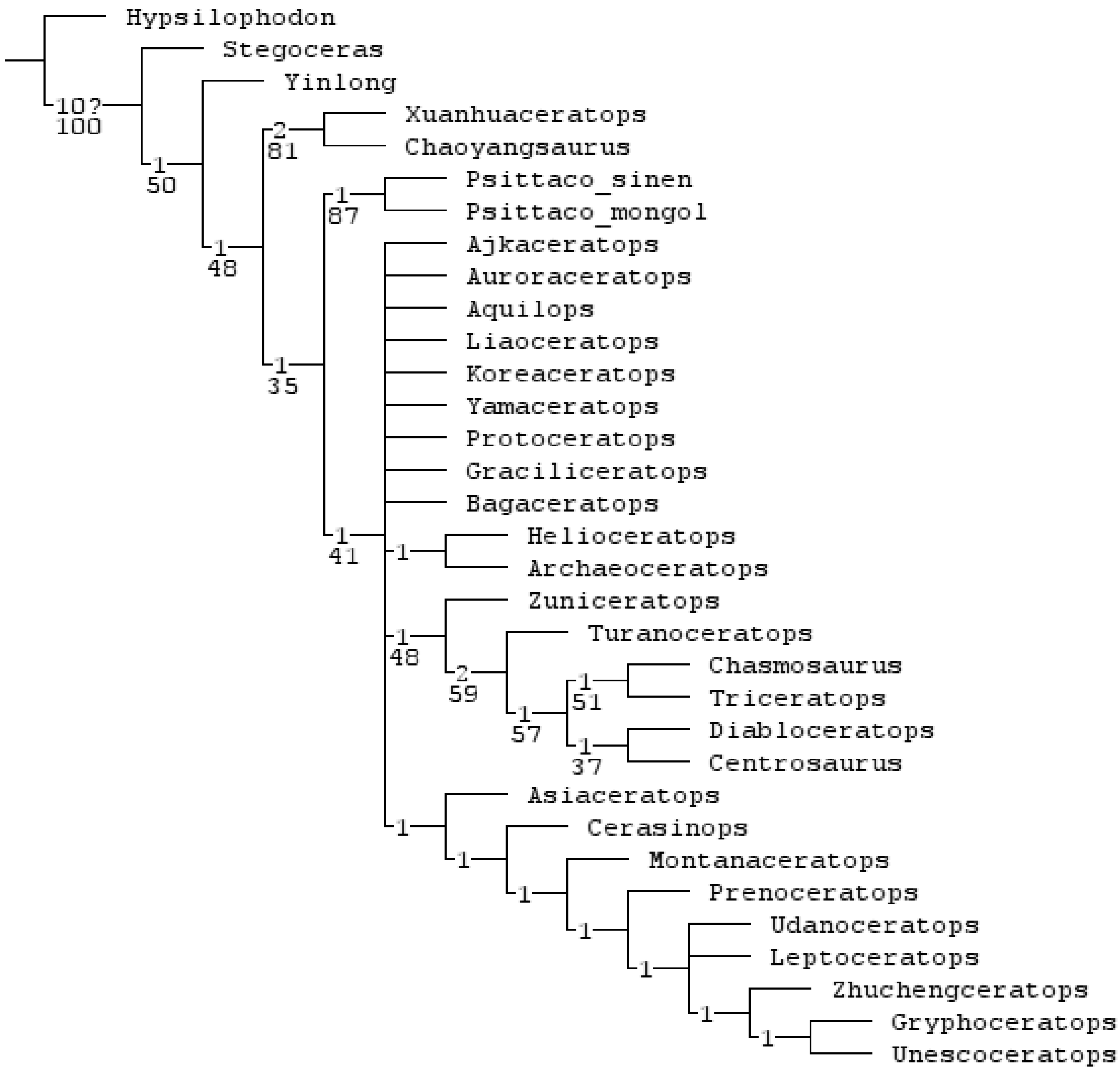

Figure S3. Branch support for the phylogenetic hypothesis recovered here, superimposed on the strict consensus of two equally most parsimonious trees. Bremer support values are indicated as the top value on each branch; bootstrap values (10,000 replicates, sampling with replacement) over 25% are indicated as the bottom value.

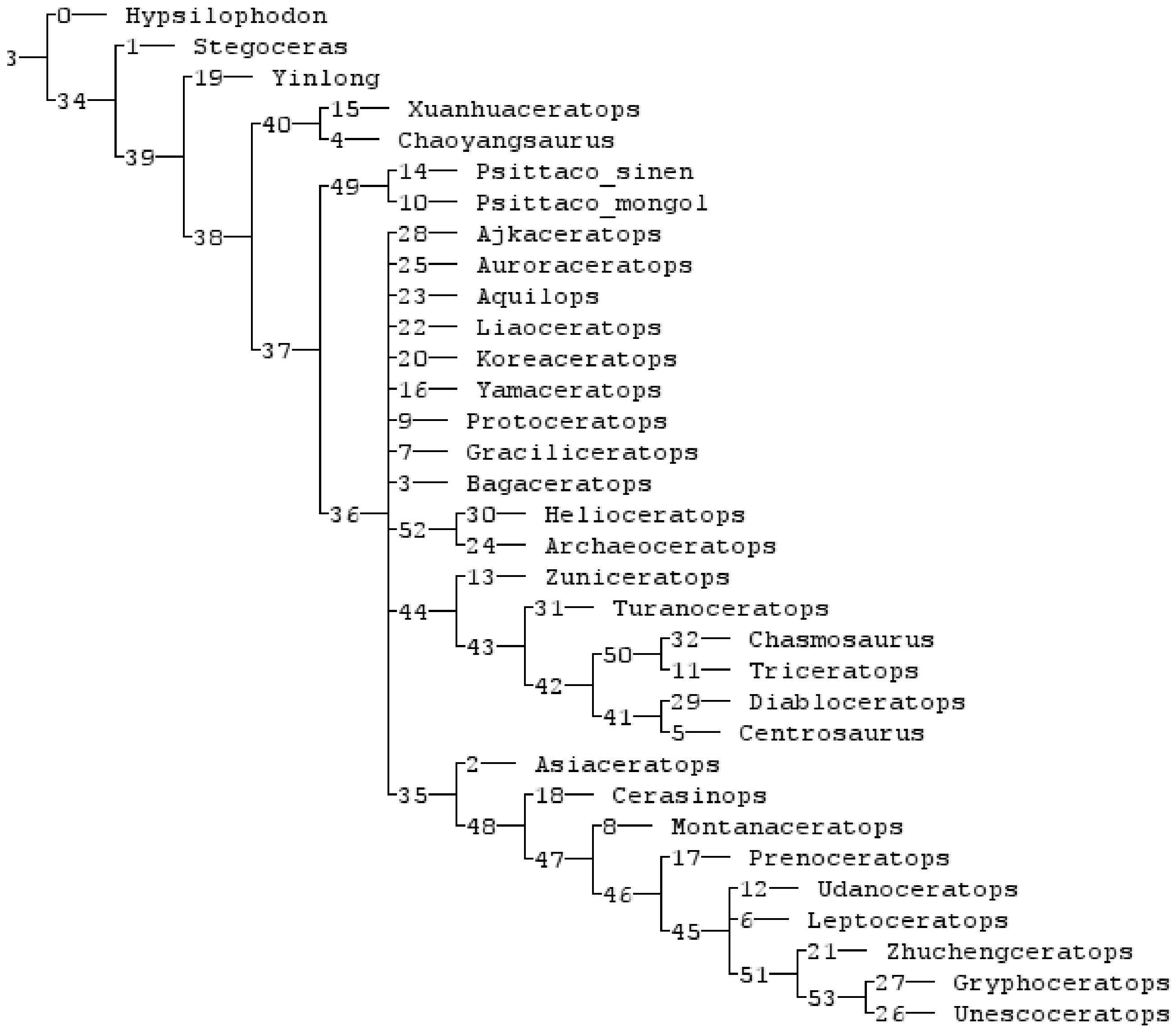

Figure S4. Strict consensus of 18 equally parsimonious trees, with selected ontogenetic-variant characters for *Aquilops americanus* scored as unknown, assuming that the holotype is a juvenile.

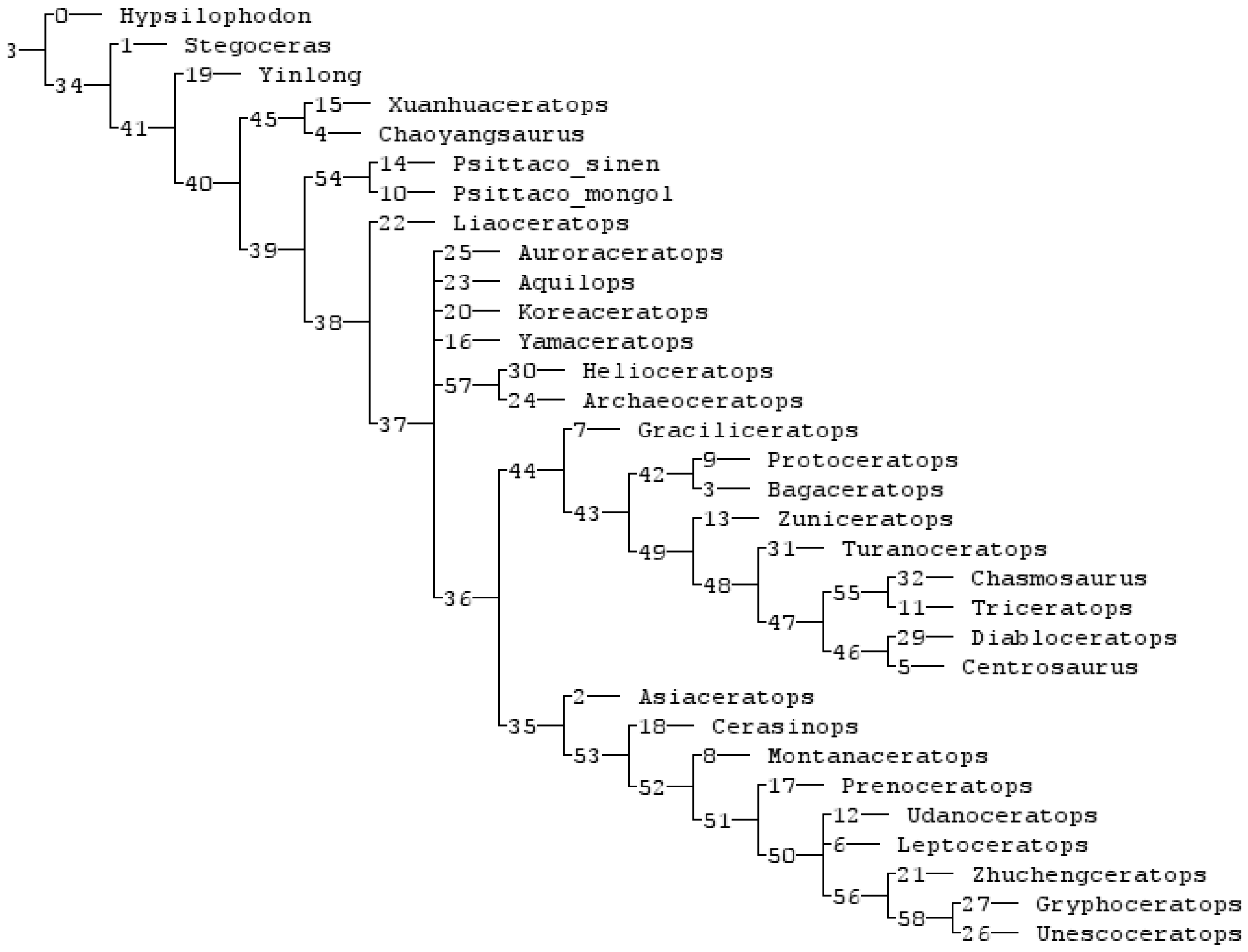

Figure S5. Strict consensus of 18 equally parsimonious trees, with selected ontogenetic-variant characters for *Aquilops americanus* scored as unknown, assuming that the holotype is a juvenile. In this version of the tree, the wildcard taxon *Ajkaceratops* has been pruned *post hoc*.

```
                                               ----+ [NA] Centrosaurus
                                           --N2+
                                           :   ----+ [NA] Diabloceratops
                                       --N6+
                                       :   :   ----+ [NA] Chasmosaurus
                                    -N8+   --N5+
                                    :  :       ----+ [NA] Triceratops
                                 N10+  :
                                 :  :  ------------+ [A] Turanoceratops
                                 :  :
                              N16+  ---------------+ [NA] Zuniceratops
                              :  :
                              :  :           ------+ [A] Bagaceratops
                              :  :     ---N13+
                           N18+  ---N15+     ------+ [E] Ajkaceratops
                           :  :        :
                           :  :        ------------+ [A] Protoceratops
                           :  :
                           :  ---------------------+ [A] Graciliceratops
                           :
                           :
                           :                 ------+ [A] Zhuchengceratops
                           :              N23+
                           :              :  :  ---+ [NA] Unescoceratops
                        N36+              :  N22+
                        :  :           N27+     ---+ [NA] Gryphoceratops
                        :  :           :  :
                        :  :           :  :   -----+ [NA] Leptoceratops
                        :  :        N29+  -N26+
                        :  :        :  :      -----+ [A] Udanoceratops
                        :  :     N31+  :
                     N38+  :     :  :  ------------+ [NA] Prenoceratops
                     :  :  :  N33+  :
                     :  :  :  :  :  ---------------+ [NA] Montanaceratops
                     :  :  N35+  :
                     :  :     :  ------------------+ [NA] Cerasinops
                  N42+  :     :
                  :  :  :     ---------------------+ [A] Asiaceratops
                  :  :  :
                  :  :  ---------------------------+ [A] Koreaceratops
                  :  :
               N46+  :              ---------------+ [A] Helioceratops
               :  :  ------------N41+
               :  :                 ---------------+ [A] Archaeoceratops
               :  :
            N48+  :               -----------------+ [A] Auroraceratops
            :  :  -------------N45+
            :  :                  -----------------+ [A] Yamaceratops
         N50+  :
         :  :  ------------------------------------+ [NA] Aquilops
         :  :
      N54+  ---------------------------------------+ [A] Liaoceratops
      :  :
      :  :                    ---------------------+ [A] Psittaco_sinen
   N58+  ------------------N53+
   :  :                       ---------------------+ [A] Psittaco_mongol
   :  :
N60+  :                     -----------------------+ [A] Xuanhuaceratops
   :  -------------------N57+
   :                        -----------------------+ [A] Chaoyangsaurus
   :
   ------------------------------------------------+ [A] Yinlong
```

Figure S6. Phylogenetic tree recovering *Ajkaceratops* as sister to *Bagaceratops*, used for DEC modeling, with nodal numbers referenced in results (Table S1).

Table S1. Temporal calibrations and geographic areas used for DEC analysis. Abbreviations: A (Asia), E (Europe), NA (North America). All ages are given in Ma and calibrated relative to the 2012 Geological Time Scale (Gradstein et al., 2012).

Taxon	Area	Age	Lower Bound	Upper Bound	Midpoint	Date Assigned	Source
Ajkaceratops	E	Santonian	86.26	83.64	84.95	85.0	Ősi et al., 2010
Aquilops	NA	middle – late Albian	108.5	104	106.25	106.3	this paper
Archaeoceratops	A	Albian	112.95	100.5	106.725	106.7	Tang et al., 2001
Asiaceratops	A	early Cenomanian	100.5	96.24	98.37	98.4	Nessov 1995
Auroraceratops	A	Albian	112.95	100.5	106.725	106.7	Tang et al., 2001
Bagaceratops	A	Campanian	83.64	72.05	77.845	77.9	Dingus et al. 2005
Centrosaurus	NA	late Campanian	76.4	70.6	73.5	75.8	Roberts et al. 2013
Cerasinops	NA	early Campanian	80	76.5	78.25	78.3	Chinnery and Horner 2007
Chaoyangsaurus	A	Bathonian	168.28	166.07	167.175	167.2	Zhao et al. 1999
Chasmosaurus	NA	late Campanian	76.4	70.6	73.5	75.8	Roberts et al. 2013
Diabloceratops	NA	middle Campanian	80.63	77.0	78.815	78.8	Roberts et al. 2013; Jinnah 2013
Graciliceratops	A	Cenomanian – Santonian	100.5	83.64	92.07	92.1	Hicks et al. 1999
Gryphoceratops	NA	late Santonian	85.23	83.64	84.435	84.4	Ryan et al. 2012
Helioceratops	A	Albian – early Turonian	112.95	92.9	102.925	102.9	Wan et al. 2013; Wang et al. 2013
Koreaceratops	A	Albian (earlier than 103 Ma)	112.95	103	107.975	108.0	Lee et al. 2011
Leptoceratops	NA	late Maastrichtian	68	66.04	67.02	68.0	Hicks et al. 2002
Liaoceratops	A	late Barremian – early Aptian	129.7	122.9	126.3	126.3	Sun et al. 2011; Chang et al. 2009
Montanaceratops	NA	early Maastrichtian	69.4	69.2	69.3	69.8	Eberth et al. 2013 (recalibrated by subtracting 0.5 Ma)
Prenoceratops	NA	late Campanian	75.0	75.0	75.0	75.0	Roberts et al. 2013
Protoceratops andrewsi	A	late Campanian	76.38	72.05	74.215	74.2	Dashzeveg et al. 2005
Psittacosaurus mongoliensis	A	Aptian / Albian	126.3	100.5	113.4	113.4	Sereno 2010
Psittacosaurus sinensis	A	Barremian	130.77	126.3	128.535	128.5	Gao and Cheng 1999
Triceratops	NA	late Maastrichtian	68	66.04	67.02	68.0	Hicks et al. 2002
Turanoceratops	A	mid- to late Turonian	92.9	89.77	91.335	91.3	Sues and Averianov 2009
Udanoceratops	A	late Campanian	76.38	72.05	74.215	74.2	Dashzeveg et al. 2005
Unescoceratops	NA	late Campanian	75.8	75.8	75.8	75.8	Roberts et al. 2013
Xuanhuaceratops	A	Late Jurassic	152.57	145.01	148.79	148.8	Zhao et al. 2006; Sullivan et al. 2009
Yamaceratops	A	Santonian – Campanian	86.26	72.05	79.155	79.2	Eberth et al. 2009
Yinlong	A	Oxfordian	163.47	157.25	160.36	160.4	Xu et al. 2006
Zhuchengceratops	A	?late Campanian – Maastrichtian	76.38	66.04	71.21	71.2	Loewen et al. 2013
Zuniceratops	NA	mid- to late Turonian	92.9	89.77	91.335	91.3	Wolfe & Kirkland 1998

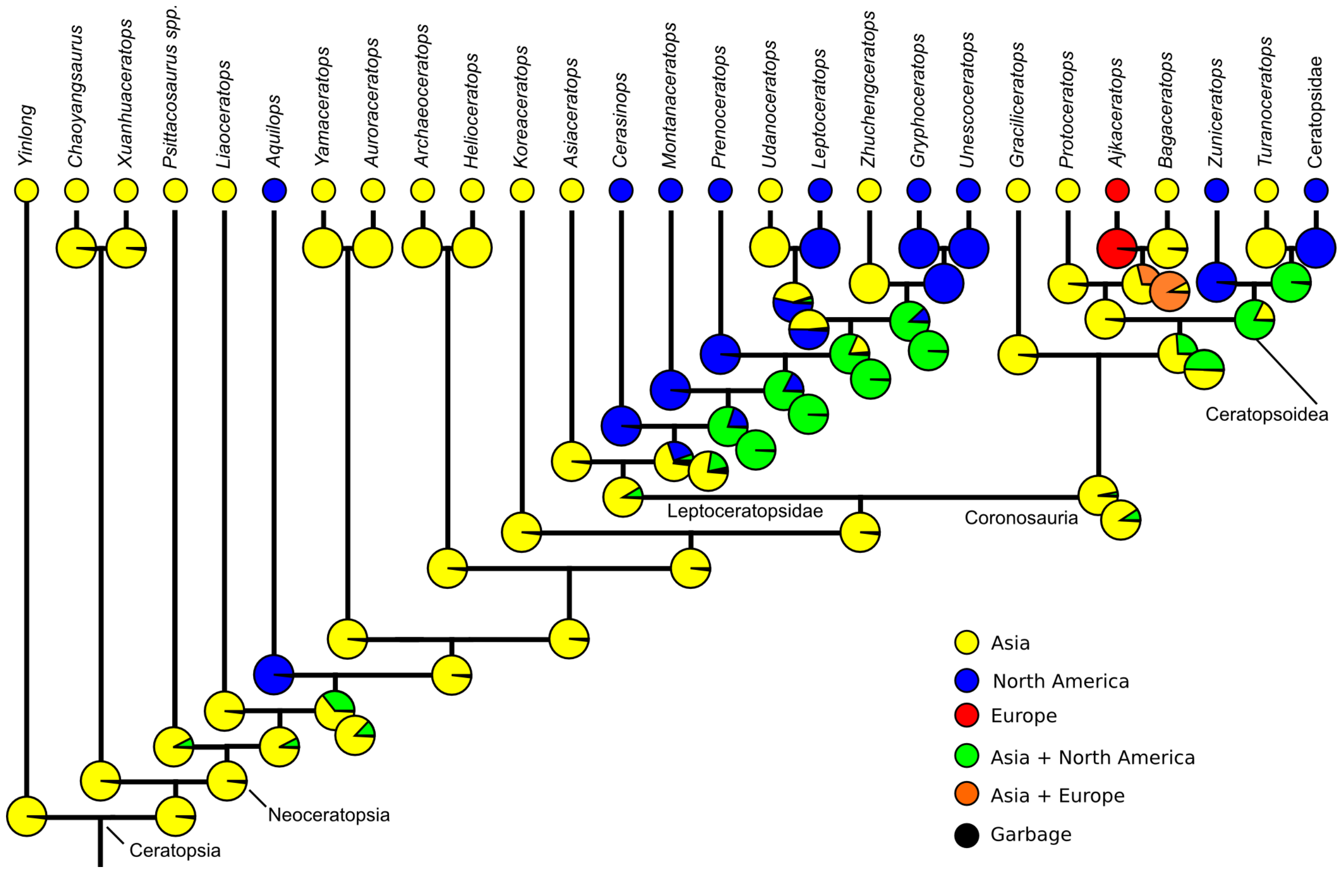

Figure S7. Hypothesis of phylogeny and biogeography of Ceratopsia, summarizing DEC modeling results. Some terminal taxa have been combined into supraspecific units; a full tree is provided in Figure S6. Localities and reconstructed ancestral ranges from DEC are plotted. The relative probabilities for the ancestral area for each branch are indicated by the colored circles. To simplify visualization, when the relative probabilities for both the "smoothed" and "strict" temporal calibrations were within with 0.05 units of each other, they are represented by a single point. When they differ by more than 0.05 units, the one at top left depicts the "smoothed" results and the one at right depicts the "strict" results. Complete results, with a precise listing of the relative probabilities for each reconstruction, are contained in Table S2.

Table S2. Node-by-node results for DEC analysis. Node numbers are indicated in Figure S6; temporal calibrations (smoothed and strict) are explained in the main text. Branch letters indicate the taxa that branch off from each node.

Node	Calibration	Branch A (Upper)	A Area	Branch B	B Area	lnL	Relative Probability
60	smoothed	*Centrosaurus*	Asia	*Yinlong*	Asia	-38.95	0.983
60	strict	*Centrosaurus*	Asia	*Yinlong*	Asia	-37.93	0.9855
58	smoothed	*Centrosaurus*	Asia	*Chaoyangsaurus*	Asia	-38.95	0.983
58	strict	*Centrosaurus*	Asia	*Chaoyangsaurus*	Asia	-37.93	0.9854
57	smoothed	*Xuanhuaceratops*	Asia	*Chaoyangsaurus*	Asia	-38.93	1
57	strict	*Xuanhuaceratops*	Asia	*Chaoyangsaurus*	Asia	-37.92	1
54	smoothed	*Centrosaurus*	Asia	*Psittaco. mongoliensis*	Asia	-39.02	0.9141
54	smoothed	*Centrosaurus*	Asia+NA	*Psittaco. mongoliensis*	Asia	-41.51	0.07583
54	strict	*Centrosaurus*	Asia	*Psittaco. mongoliensis*	Asia	-38.05	0.8755
54	strict	*Centrosaurus*	Asia+NA	*Psittaco. mongoliensis*	Asia	-40.02	0.1218
53	smoothed	*Psittaco. sinensis*	Asia	*Psittaco. mongoliensis*	Asia	-38.93	1
53	strict	*Psittaco. sinensis*	Asia	*Psittaco. mongoliensis*	Asia	-37.92	1
50	smoothed	*Centrosaurus*	Asia	*Liaoceratops*	Asia	-39.4	0.6295
50	smoothed	*Centrosaurus*	Asia+NA	*Liaoceratops*	Asia	-40	0.3449
50	strict	*Centrosaurus*	Asia	*Liaoceratops*	Asia	-38.08	0.8506
50	strict	*Centrosaurus*	Asia+NA	*Liaoceratops*	Asia	-39.96	0.1297
48	smoothed	*Centrosaurus*	Asia	*Aquilops*	NA	-38.95	0.9845
48	strict	*Centrosaurus*	Asia	*Aquilops*	NA	-37.93	0.9905
46	smoothed	*Centrosaurus*	Asia	*Yamaceratops*	Asia	-38.95	0.984
46	strict	*Centrosaurus*	Asia	*Yamaceratops*	Asia	-37.93	0.9905
45	smoothed	*Auroraceratops*	Asia	*Yamaceratops*	Asia	-38.93	1
45	strict	*Auroraceratops*	Asia	*Yamaceratops*	Asia	-37.92	1
42	smoothed	*Centrosaurus*	Asia	*Archaeoceratops*	Asia	-38.95	0.9826
42	strict	*Centrosaurus*	Asia	*Archaeoceratops*	Asia	-37.93	0.9904
41	smoothed	*Helioceratops*	Asia	*Archaeoceratops*	Asia	-38.93	1
41	strict	*Helioceratops*	Asia	*Archaeoceratops*	Asia	-37.92	1
38	smoothed	*Centrosaurus*	Asia	*Koreaceratops*	Asia	-38.96	0.9786
38	strict	*Centrosaurus*	Asia	*Koreaceratops*	Asia	-37.93	0.9901
36	smoothed	*Centrosaurus*	Asia	*Asiaceratops*	Asia	-39.08	0.8665
36	smoothed	*Centrosaurus*	Asia	*Asiaceratops*	Asia+NA	-41.55	0.07306
36	smoothed	*Centrosaurus*	Asia+NA	*Asiaceratops*	Asia	-42.32	0.03372
36	strict	*Centrosaurus*	Asia	*Asiaceratops*	Asia	-38.11	0.8253
36	strict	*Centrosaurus*	Asia+NA	*Asiaceratops*	Asia	-40.11	0.112
36	strict	*Centrosaurus*	Asia	*Asiaceratops*	Asia+NA	-41.2	0.03766
35	smoothed	*Zhuchengceratops*	Asia	*Asiaceratops*	Asia	-39.21	0.7572
35	smoothed	*Zhuchengceratops*	NA	*Asiaceratops*	Asia	-40.63	0.1841
35	smoothed	*Zhuchengceratops*	Asia+NA	*Asiaceratops*	Asia	-41.77	0.05875
35	strict	*Zhuchengceratops*	Asia	*Asiaceratops*	Asia	-37.97	0.9435
35	strict	*Zhuchengceratops*	Asia+NA	*Asiaceratops*	Asia	-41.37	0.03167
33	smoothed	*Zhuchengceratops*	Asia+NA	*Cerasinops*	NA	-39.16	0.7992
33	smoothed	*Zhuchengceratops*	NA	*Cerasinops*	NA	-40.65	0.1802
33	strict	*Zhuchengceratops*	Asia+NA	*Cerasinops*	NA	-37.94	0.978
31	smoothed	*Zhuchengceratops*	Asia+NA	*Montanaceratops*	NA	-39.14	0.8169
31	smoothed	*Zhuchengceratops*	NA	*Montanaceratops*	NA	-40.69	0.1725
31	strict	*Zhuchengceratops*	Asia+NA	*Montanaceratops*	NA	-37.94	0.9782
29	smoothed	*Zhuchengceratops*	Asia+NA	*Prenoceratops*	NA	-39.11	0.8363

29	smoothed	*Zhuchengceratops*	NA	*Prenoceratops*	NA	-40.83	0.1506
29	strict	*Zhuchengceratops*	Asia+NA	*Prenoceratops*	NA	-37.94	0.9762
27	smoothed	*Zhuchengceratops*	Asia+NA	*Udanoceratops*	Asia	-39.8	0.4192
27	smoothed	*Zhuchengceratops*	Asia+NA	*Udanoceratops*	NA	-39.8	0.4192
27	smoothed	*Zhuchengceratops*	NA	*Udanoceratops*	NA	-41.37	0.0872
27	smoothed	*Zhuchengceratops*	NA	*Udanoceratops*	Asia+NA	-42.38	0.032
27	strict	*Zhuchengceratops*	Asia+NA	*Udanoceratops*	Asia	-38.64	0.4854
27	strict	*Zhuchengceratops*	Asia+NA	*Udanoceratops*	NA	-38.64	0.4854
26	smoothed	*Leptoceratops*	NA	*Udanoceratops*	Asia	-38.93	1
26	strict	*Leptoceratops*	NA	*Udanoceratops*	Asia	-37.92	1
23	smoothed	*Zhuchengceratops*	Asia	*Gryphoceratops*	NA	-38.93	1
23	strict	*Zhuchengceratops*	Asia	*Gryphoceratops*	NA	-37.92	1
22	smoothed	*Unescoceratops*	NA	*Gryphoceratops*	NA	-38.93	1
22	strict	*Unescoceratops*	NA	*Gryphoceratops*	NA	-37.92	1
18	smoothed	*Centrosaurus*	Asia	*Graciliceratops*	Asia	-39.27	0.7147
18	smoothed	*Centrosaurus*	Asia+NA	*Graciliceratops*	Asia	-40.23	0.2739
18	strict	*Centrosaurus*	Asia+NA	*Graciliceratops*	Asia	-38.6	0.5033
18	strict	*Centrosaurus*	Asia	*Graciliceratops*	Asia	-38.64	0.4844
16	smoothed	*Centrosaurus*	Asia+NA	*Protoceratops*	Asia	-39.15	0.8082
16	smoothed	*Centrosaurus*	Asia	*Protoceratops*	Asia	-40.68	0.1738
16	strict	*Centrosaurus*	Asia+NA	*Protoceratops*	Asia	-38.15	0.7921
16	strict	*Centrosaurus*	Asia	*Protoceratops*	Asia	-39.58	0.19
15	smoothed	*Bagaceratops*	Asia	*Protoceratops*	Asia	-39.28	0.7073
15	smoothed	*Bagaceratops*	Asia+Europe	*Protoceratops*	Asia	-40.18	0.2893
15	strict	*Bagaceratops*	Asia+Europe	*Protoceratops*	Asia	-38	0.9194
15	strict	*Bagaceratops*	Asia	*Protoceratops*	Asia	-40.45	0.0797
13	smoothed	*Bagaceratops*	Asia	*Ajkaceratops*	Europe	-38.93	1
13	strict	*Bagaceratops*	Asia	*Ajkaceratops*	Europe	-37.92	1
10	smoothed	*Centrosaurus*	Asia+NA	*Zuniceratops*	NA	-38.94	0.9956
10	strict	*Centrosaurus*	Asia+NA	*Zuniceratops*	NA	-37.92	0.9943
8	smoothed	*Centrosaurus*	NA	*Turanoceratops*	Asia	-38.93	1
8	strict	*Centrosaurus*	NA	*Turanoceratops*	Asia	-37.92	1
6	smoothed	*Centrosaurus*	NA	*Triceratops*	NA	-38.93	1
6	strict	*Centrosaurus*	NA	*Triceratops*	NA	-37.92	1
5	smoothed	*Chasmosaurus*	NA	*Triceratops*	NA	-38.93	1
5	strict	*Chasmosaurus*	NA	*Triceratops*	NA	-37.92	1
2	smoothed	*Centrosaurus*	NA	*Diabloceratops*	NA	-38.93	1
2	strict	*Centrosaurus*	NA	*Diabloceratops*	NA	-37.92	1

Literature Cited

Chang, S., H. Zhang, P. R. Renne, and Y. Fang. 2009. High-precision 40Ar/39Ar age for the Jehol Biota. Palaeogeography, Palaeoclimatology, Palaeoecology 280:94–104.

Chinnery, B. J., and J. R. Horner. 2007. A new neoceratopsian dinosaur linking North American and Asian taxa. Journal of Vertebrate Paleontology 27:625–641.

Dashzeveg, D., L. Dingus, D. B. Loope, C. C. Swisher, T. Dulam, and M. R. Sweeney. 2005. New stratigraphic subdivision, depositional environment, and age estimate for the Upper Cretaceous Djadokhta Formation, Southern Ulan Nur Basin, Mongolia. American Museum Novitates 3498:1–31.

Dingus, L., D. B. Loope, D. Dashzeveg, C. C. Swisher, C. Minjin, M. J. Novacek, and M. Norell. 2008. The geology of Ukhaa Tolgod (Djadokhta Formation, Upper Cretaceous, Nemegt Basin, Mongolia). American Museum Novitates 3616:1–40.

Eberth, D. A., Y. Kobayashi, Y.-N. Lee, O. Mateus, F. Therrien, D. K. Zelenitsky, and M. A. Norell. 2009. Assignment of *Yamaceratops dorngobiensis* and associated redbeds at Shine Us Khudag (eastern Gobi, Dorngobi Province, Mongolia) to the redescribed Javkhlant Formation (Upper Cretaceous). Journal of Vertebrate Paleontology 29:295–302.

Eberth, D. A., D. C. Evans, D. B. Brinkman, F. Therrien, D. H. Tanke, and L. S. Russell. 2013. Dinosaur biostratigraphy of the Edmonton Group (Upper Cretaceous), Alberta, Canada: evidence for climate influence. Canadian Journal of Earth Sciences 50:701–726.

Gao, K., and Z. Cheng. 1999. A new lizard from the Lower Cretaceous of Shandong, China. Journal of Vertebrate Paleontology 19:456–465.

Gradstein, F. M., J. G. Ogg, M. Schmitz, and G. Ogg (eds.). 2012. The Geologic Time Scale 2012. Elsevier, 1176 pp.

Hicks, J. F., D. L. Brinkman, D. J. Nichols, and M. Watabe. 1999. Paleomagnetic and palynologic analyses of Albian to Santonian strata at Bayn Shireh, Burkhant, and Khuren Dukh, eastern Gobi Desert, Mongolia. Cretaceous Research 20:829–850.

Hicks, J. F., K. R. Johnson, J. D. Obradovich, L. Tauxe, and D. Clark. 2002. Magnetostratigraphy and geochronology of the Hell Creek and basal Fort Union Formations of southwestern North Dakota and a recalibration of the age of the Cretaceous-Tertiary boundary. Geological Society of America Special Papers 361:35–55.

Lee, Y.-N., M. Ryan, and Y. Kobayashi. 2011. The first ceratopsian dinosaur from South Korea. Naturwissenschaften 98:39–49.

Loewen, M. A., R. B. Irmis, J. J. W. Sertich, P. J. Currie, and S. D. Sampson. 2013. Tyrant dinosaur evolution tracks the rise and fall of Late Cretaceous oceans. PLoS ONE 8:e79420.

Makovicky, P., and M. A. Norell. 2006. *Yamaceratops dorngobiensis*, a new primitive ceratopsian (Dinosauria: Ornithischia) from the Cretaceous of Mongolia. American Museum Novitates 3530:1–42.

Makovicky, P. J. 2010. A redescription of the *Montanoceratops cerorhynchus* holotype, with a review of referred material; pp. 68–82 in M. J. Ryan, B. J. Chinnery-Allgeier, and D. A. Eberth (eds.), New Perspectives on Horned Dinosaurs: The Royal Tyrrell Museum Ceratopsian Symposium. Indiana University Press, Bloomington, Indiana.

Nessov, L. A. 1995. Dinozavri Severnoi Yevrazii: Novye Dannye O Sostave Kompleksov, Ekologii I Paleobiogeografii [Dinosaurs of Northern Eurasia: New Data about Assemblages, Ecology, and Paleobiogeography]. Institute for Scientific Research on the Earth's Crust, St. Petersburg State University, St. Petersburg, St. Petersburg, pp.

Ősi, A., R. J. Butler, and D. B. Weishampel. 2010. A Late Cretaceous ceratopsian dinosaur from Europe with Asian affinities. Nature 465:466–468.

Roberts, E. M., S. D. Sampson, A. Deino, S. Bowring, and R. Buchwaldt. 2013. The Kaiparowits

Formation: a remarkable record of Late Cretaceous terrestrial environments, ecosystems and evolution in Western North America; pp. 85–106 in A. L. Titus and M. A. Loewen (eds.), At the Top of the Grand Staircase: The Late Cretaceous of Southern Utah. Indiana University Press, Bloomington, Indiana.

Ryan, M. J., D. C. Evans, P. J. Currie, C. M. Brown, and D. Brinkman. 2012. New leptoceratopsids from the Upper Cretaceous of Alberta, Canada. Cretaceous Research 35:69–80.

Sereno, P. C. 2010. Taxonomy, cranial morphology, and relationships of parrot-beaked dinosaurs (Ceratopsia: *Psittacosaurus*); pp. 21–58 in M. Ryan, B. J. Chinnery-Allgeier, and D. A. Eberth (eds.), New Perspectives on Horned Dinosaurs: The Royal Tyrrell Museum Ceratopsian Symposium. Indiana University Press, Bloomington.

Sues, H.-D., and A. Averianov. 2009. *Turanoceratops tardabilis*-the first ceratopsid dinosaur from Asia. Die Naturwissenschaften 96:645–652.

Sullivan, C., D. W. E. Hone, T. D. Cope, Y. Liu, and J. Liu. 2009. A new occurrence of small theropod tracks in the Houcheng (Tuchengzi) Formation of Hebei Province, China. Vertebrata PalAsiatica 37:45–52.

Sun, G., D. L. Dilcher, H. Wang, and Z. Chen. 2011. A eudicot from the Early Cretaceous of China. Nature 471:625–628.

Tang, F., Z.-X. Luo, Z.-H. Zhou, H.-L. You, J. A. Georgi, Z.-L. Tang, and X.-Z. Wang. 2001. Biostratigraphy and palaeoenvironment of the dinosaur-bearing sediments in Lower Cretaceous of Mazongshan area, Gansu Province, China. Cretaceous Research 22:115–129.

Wan, X., J. Zhao, R. W. Scott, P. Wang, Z. Feng, Q. Huang, and D. Xi. 2013. Late Cretaceous stratigraphy, Songliao Basin, NE China: SK1 cores. Palaeogeography, Palaeoclimatology, Palaeoecology 385:31–43.

Wang, C., Z. Feng, L. Zhang, Y. Huang, K. Cao, P. Wang, and B. Zhao. 2013. Cretaceous paleogeography and paleoclimate and the setting of SKI borehole sites in Songliao Basin, northeast China. Palaeogeography, Palaeoclimatology, Palaeoecology 385:17–30.

Wolfe, D. G., and J. I. Kirkland. 1998. *Zuniceratops christopheri* n. gen. & n. sp., a ceratopsian dinosaur from the Moreno Hill Formation (Cretaceous, Turonian) of west-central New Mexico. Bulletin of the New Mexico Museum of Natural History and Science 14:303–317.

Xu, X., C. A. Forster, J. M. Clark, and J. Mo. 2006. A basal ceratopsian with transitional features from the Late Jurassic of northwestern China. Proceedings of the Royal Society B: Biological Sciences 273:2135–2140.

Zhao, X., Z. Cheng, and X. Xu. 1999. The earliest ceratopsian from the Tuchengzi Formation of Liaoning, China. Journal of Vertebrate Paleontology 19:681–691.

Zhao, X., C. Zhengwu, X. Xu, and P. J. Makovicky. 2006. A new ceratopsian from the Upper Jurassic Houcheng Formation of Hebei, China. Acta Geologica Sinica 80:467–473.
