# Supplementary figures and images for: A Ceratopsian Dinosaur from the Lower Cretaceous of Western North America, and the Biogeography of Neoceratopsia
Source: PLoS One. 2014 Dec 10;9(12):e112055. doi: 10.1371/journal.pone.0112055 (PMC4262212; doi:10.1371/journal.pone.0112055)

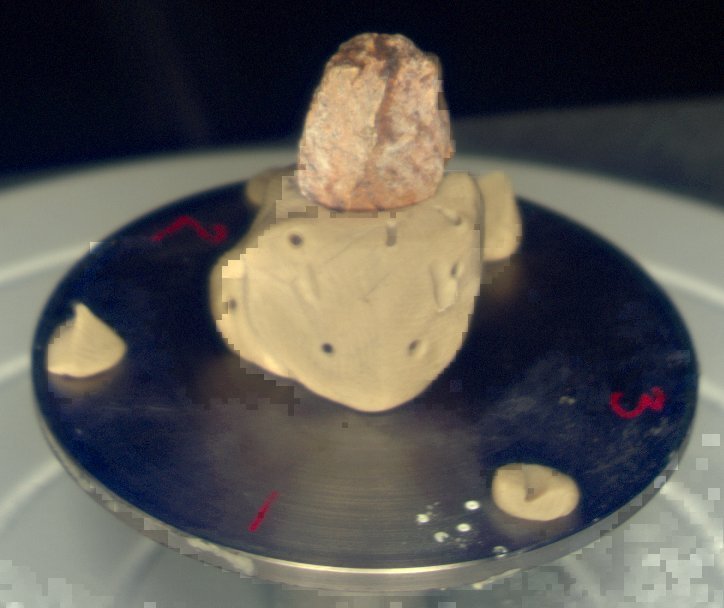

Supplement: File S7 — Surface scans of elements from the lower jaw of Aquilops americanus , OMNH 34557 (holotype). (ZIP) [file pone.0112055.s007.zip › Cranial Dentary/DentaryB_T4.jpg]

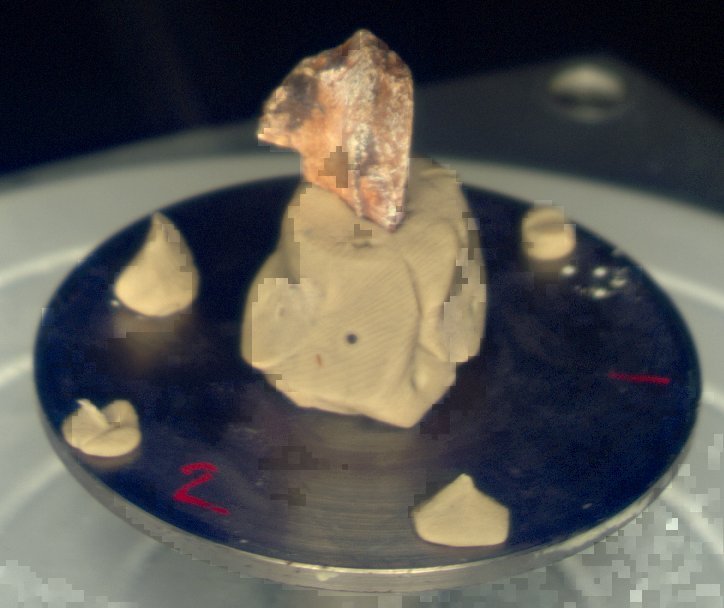

Supplement: File S7 — Surface scans of elements from the lower jaw of Aquilops americanus , OMNH 34557 (holotype). (ZIP) [file pone.0112055.s007.zip › Cranial Dentary/DentaryB_T2.jpg]

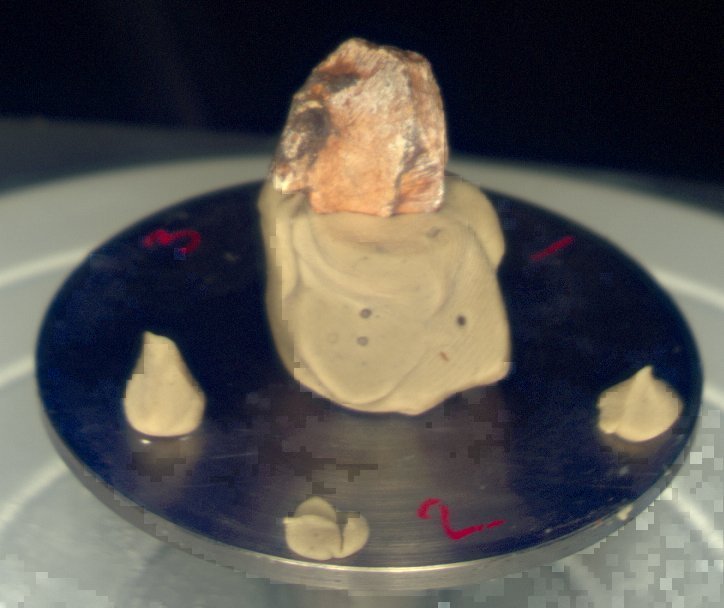

Supplement: File S7 — Surface scans of elements from the lower jaw of Aquilops americanus , OMNH 34557 (holotype). (ZIP) [file pone.0112055.s007.zip › Cranial Dentary/DentaryB_T1.jpg]

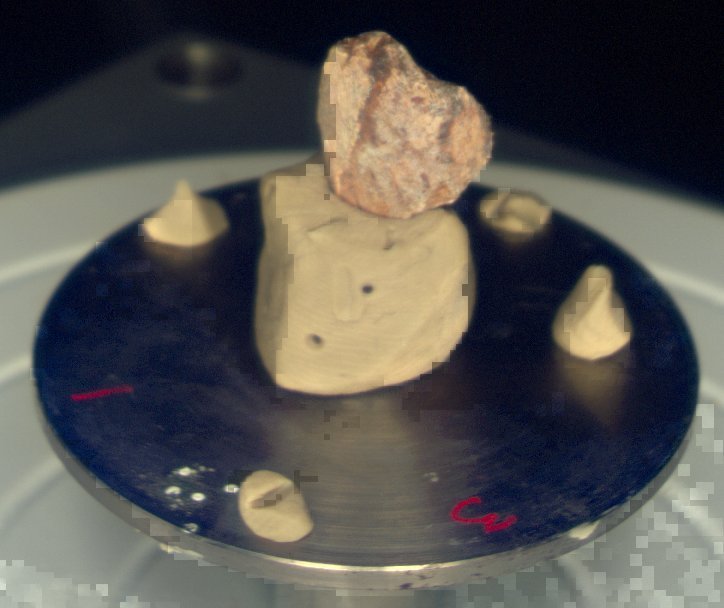

Supplement: File S7 — Surface scans of elements from the lower jaw of Aquilops americanus , OMNH 34557 (holotype). (ZIP) [file pone.0112055.s007.zip › Cranial Dentary/DentaryB_T5.jpg]

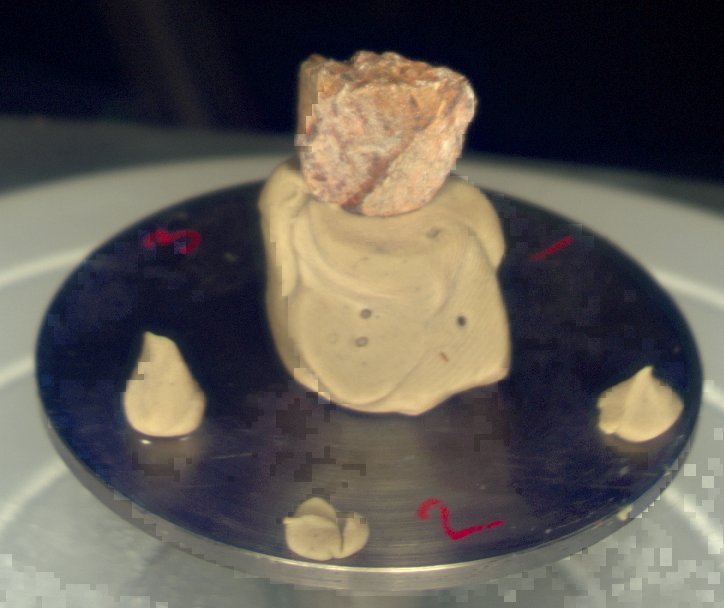

Supplement: File S7 — Surface scans of elements from the lower jaw of Aquilops americanus , OMNH 34557 (holotype). (ZIP) [file pone.0112055.s007.zip › Cranial Dentary/DentaryB_T8.jpg]

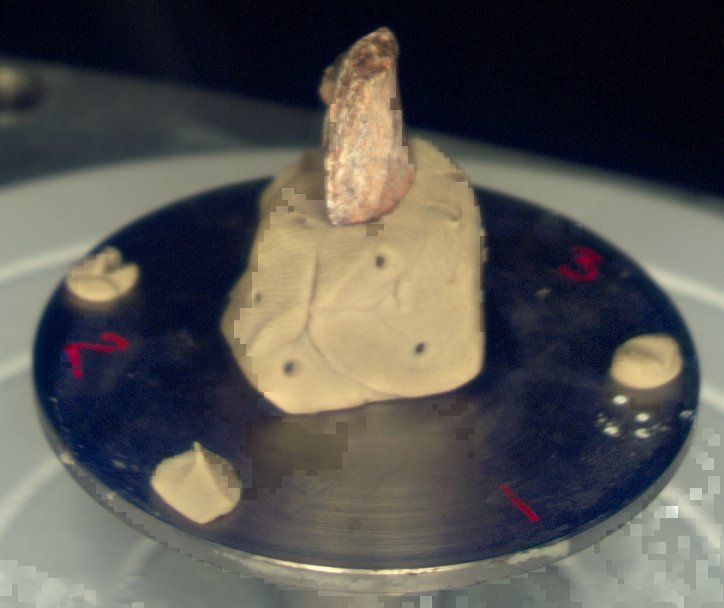

Supplement: File S7 — Surface scans of elements from the lower jaw of Aquilops americanus , OMNH 34557 (holotype). (ZIP) [file pone.0112055.s007.zip › Cranial Dentary/DentaryB_T3.jpg]

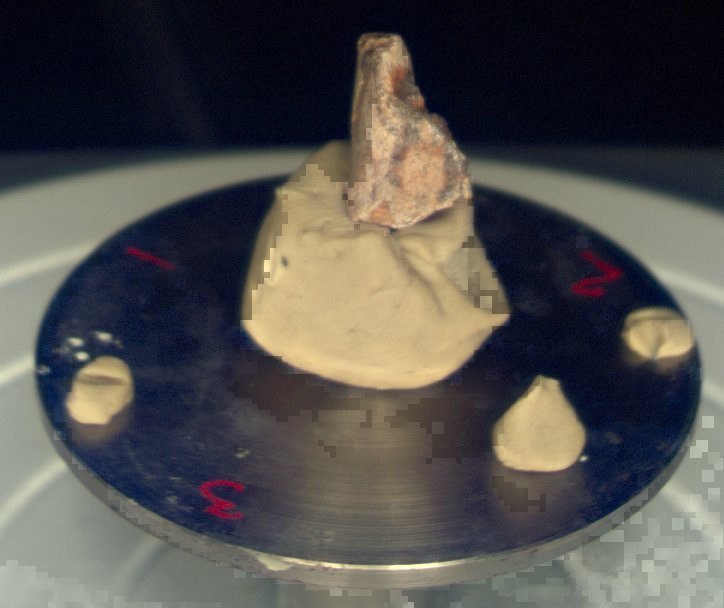

Supplement: File S7 — Surface scans of elements from the lower jaw of Aquilops americanus , OMNH 34557 (holotype). (ZIP) [file pone.0112055.s007.zip › Cranial Dentary/DentaryB_T6.jpg]

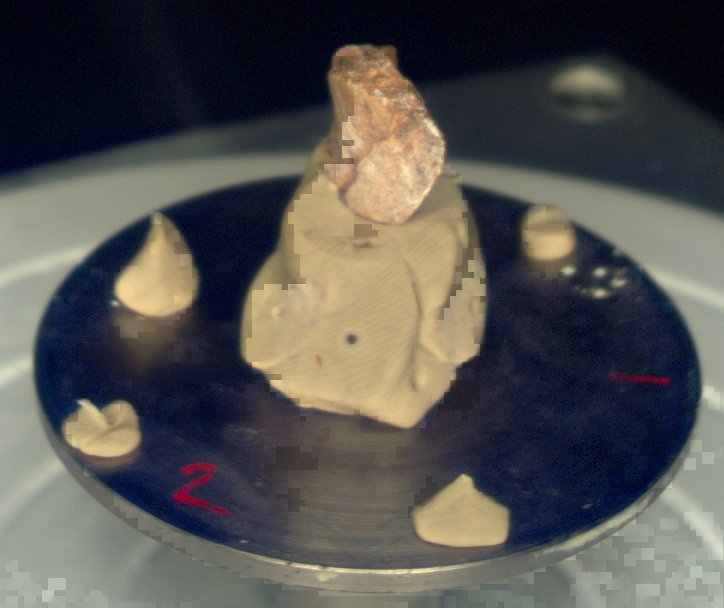

Supplement: File S7 — Surface scans of elements from the lower jaw of Aquilops americanus , OMNH 34557 (holotype). (ZIP) [file pone.0112055.s007.zip › Cranial Dentary/DentaryB_T9.jpg]

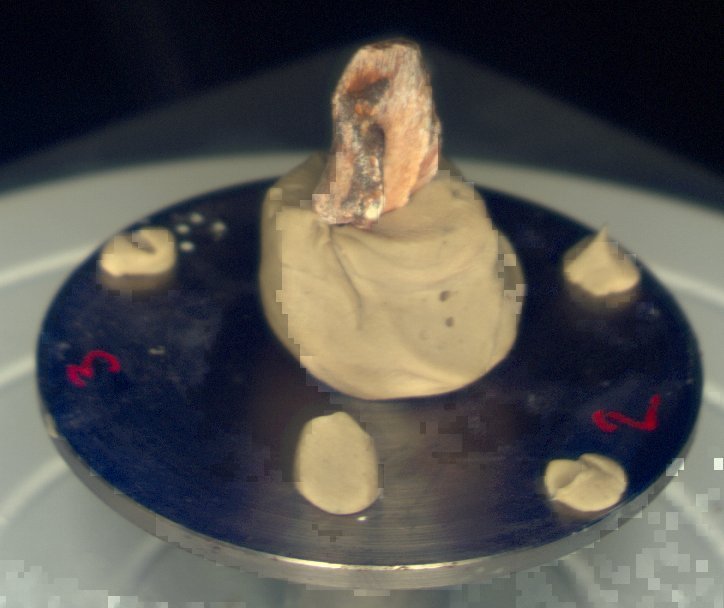

Supplement: File S7 — Surface scans of elements from the lower jaw of Aquilops americanus , OMNH 34557 (holotype). (ZIP) [file pone.0112055.s007.zip › Cranial Dentary/DentaryB_T7.jpg]

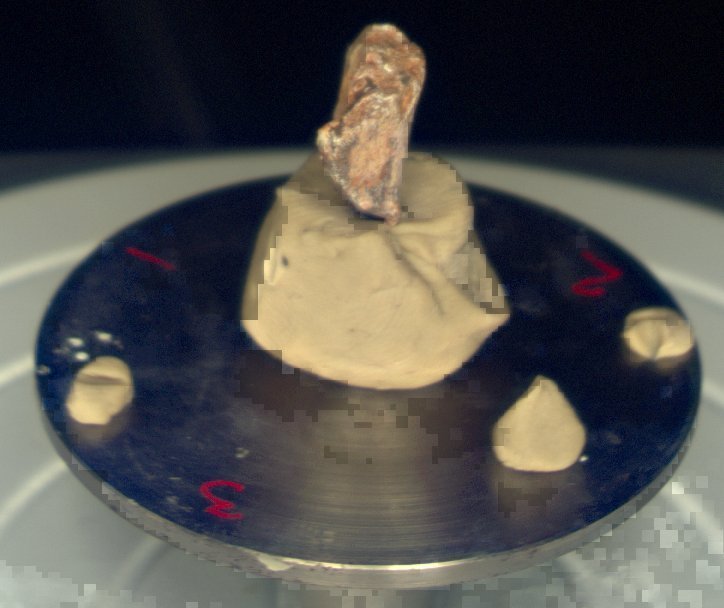

Supplement: File S7 — Surface scans of elements from the lower jaw of Aquilops americanus , OMNH 34557 (holotype). (ZIP) [file pone.0112055.s007.zip › Cranial Dentary/DentaryB_T13.jpg]

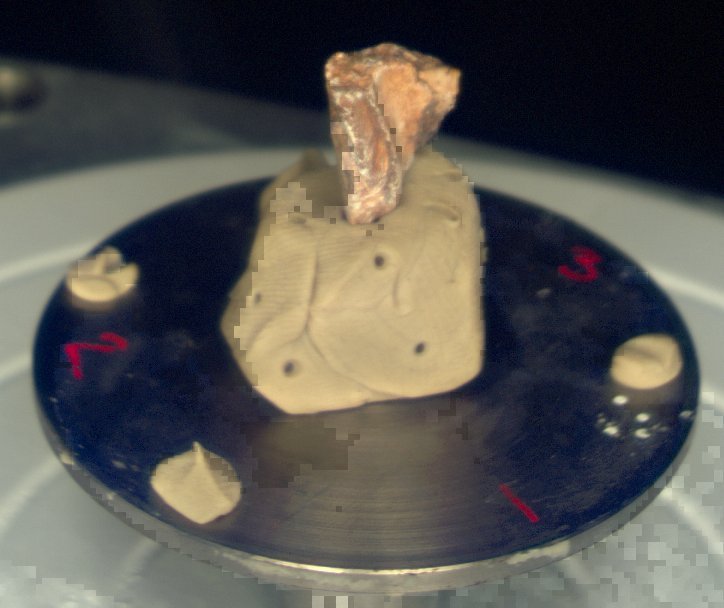

Supplement: File S7 — Surface scans of elements from the lower jaw of Aquilops americanus , OMNH 34557 (holotype). (ZIP) [file pone.0112055.s007.zip › Cranial Dentary/DentaryB_T10.jpg]

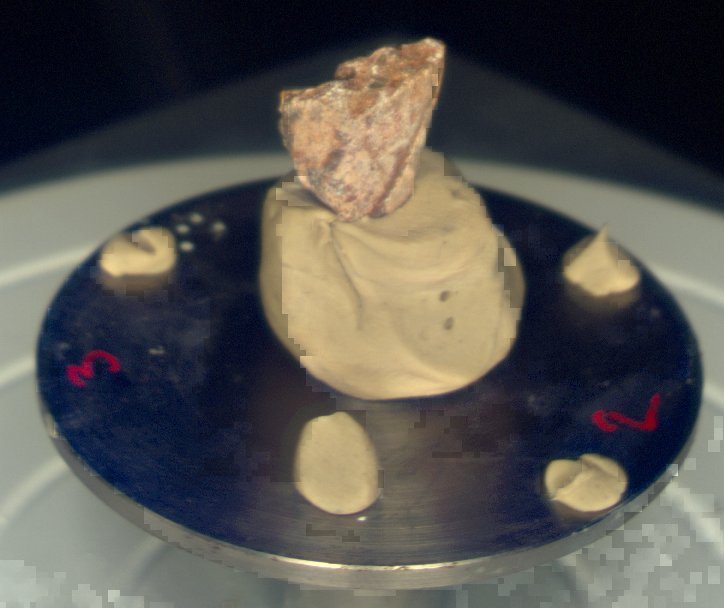

Supplement: File S7 — Surface scans of elements from the lower jaw of Aquilops americanus , OMNH 34557 (holotype). (ZIP) [file pone.0112055.s007.zip › Cranial Dentary/DentaryB_T14.jpg]

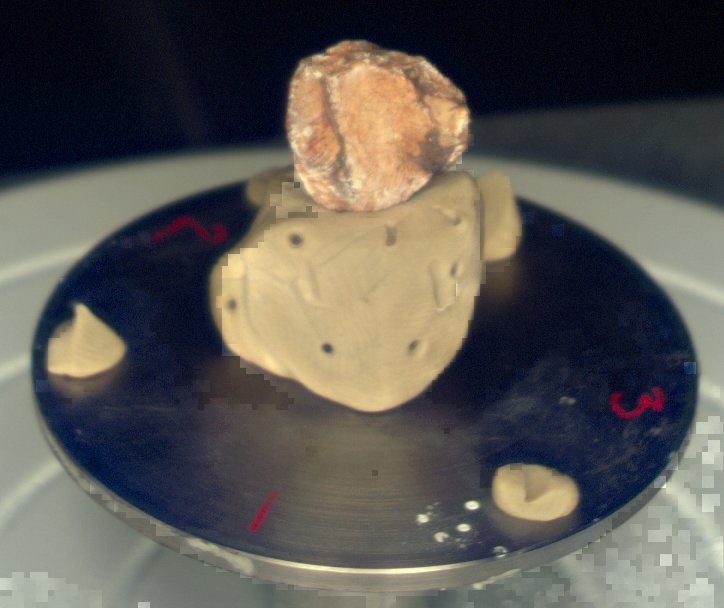

Supplement: File S7 — Surface scans of elements from the lower jaw of Aquilops americanus , OMNH 34557 (holotype). (ZIP) [file pone.0112055.s007.zip › Cranial Dentary/DentaryB_T11.jpg]

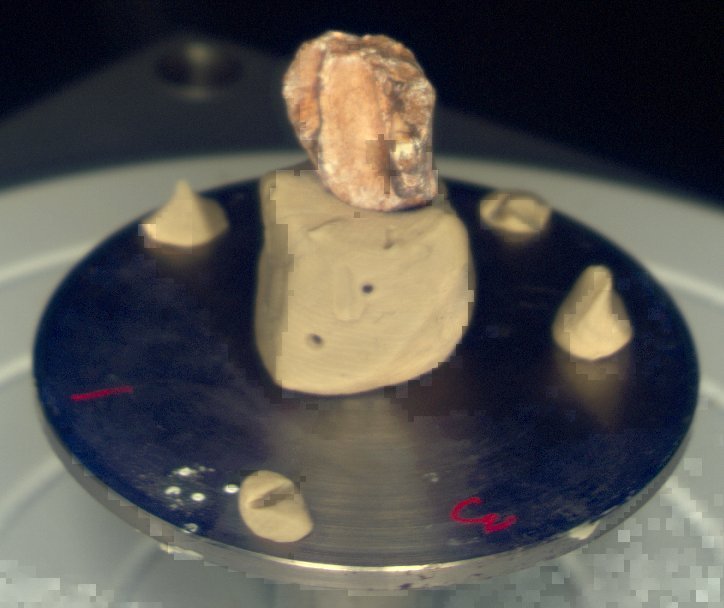

Supplement: File S7 — Surface scans of elements from the lower jaw of Aquilops americanus , OMNH 34557 (holotype). (ZIP) [file pone.0112055.s007.zip › Cranial Dentary/DentaryB_T12.jpg]

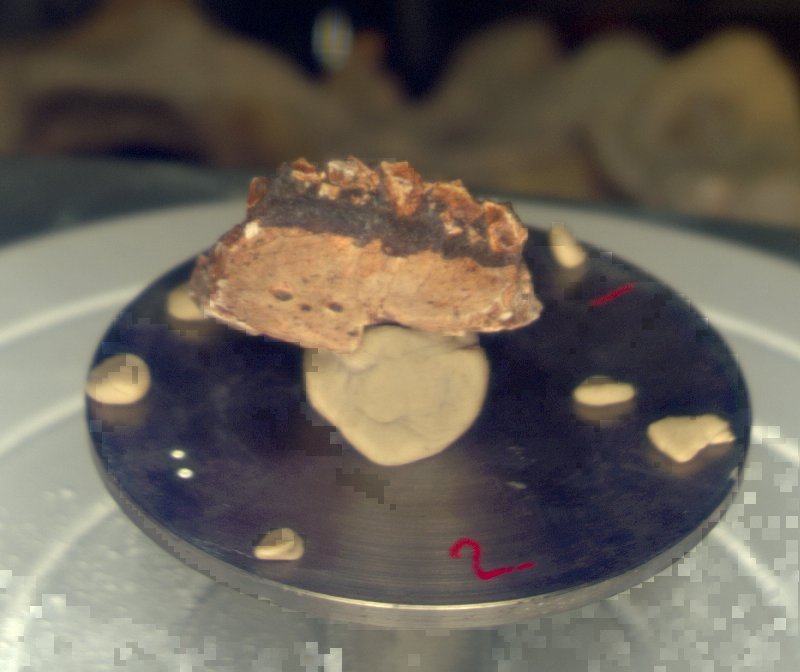

Supplement: File S7 — Surface scans of elements from the lower jaw of Aquilops americanus , OMNH 34557 (holotype). (ZIP) [file pone.0112055.s007.zip › Posterior Dentary/MainDentary_T9.jpg]

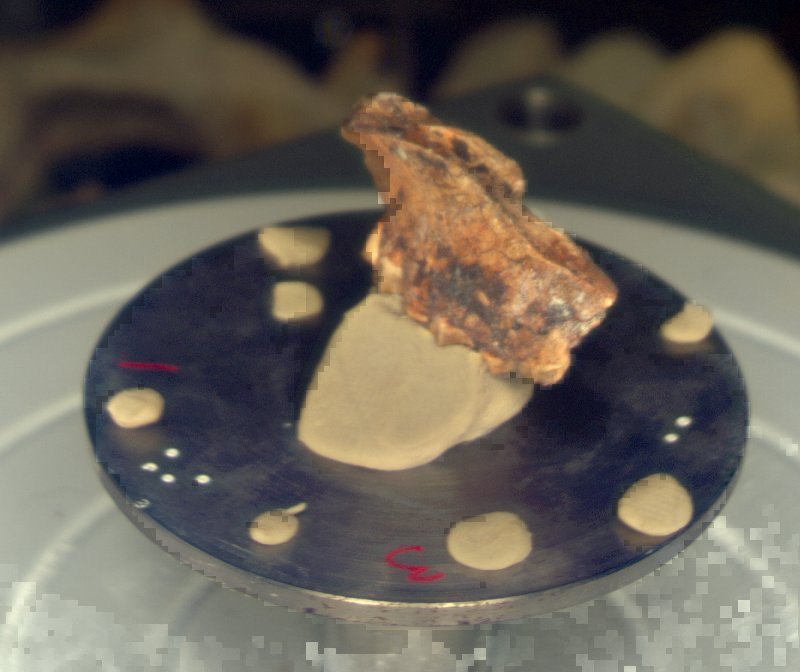

Supplement: File S7 — Surface scans of elements from the lower jaw of Aquilops americanus , OMNH 34557 (holotype). (ZIP) [file pone.0112055.s007.zip › Posterior Dentary/MainDentary_T6.jpg]

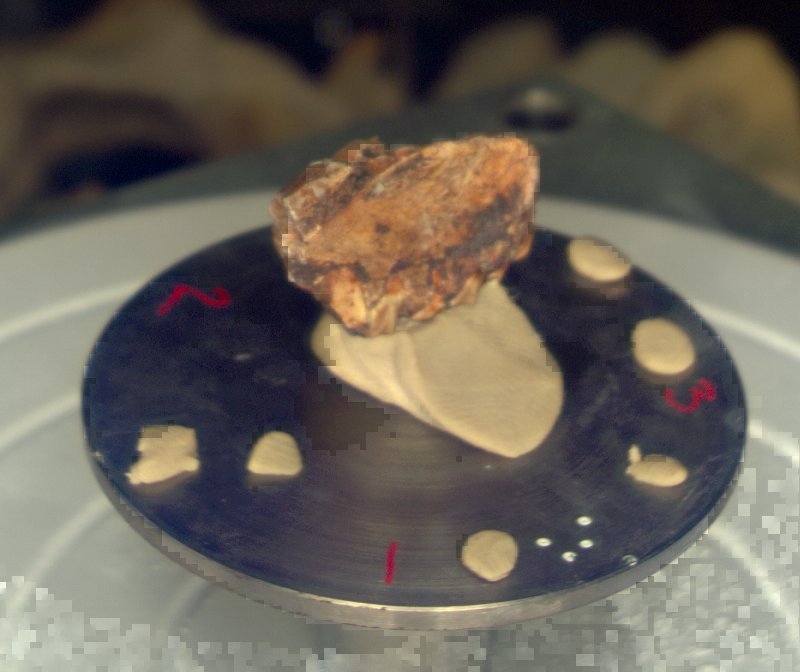

Supplement: File S7 — Surface scans of elements from the lower jaw of Aquilops americanus , OMNH 34557 (holotype). (ZIP) [file pone.0112055.s007.zip › Posterior Dentary/MainDentary_T4.jpg]

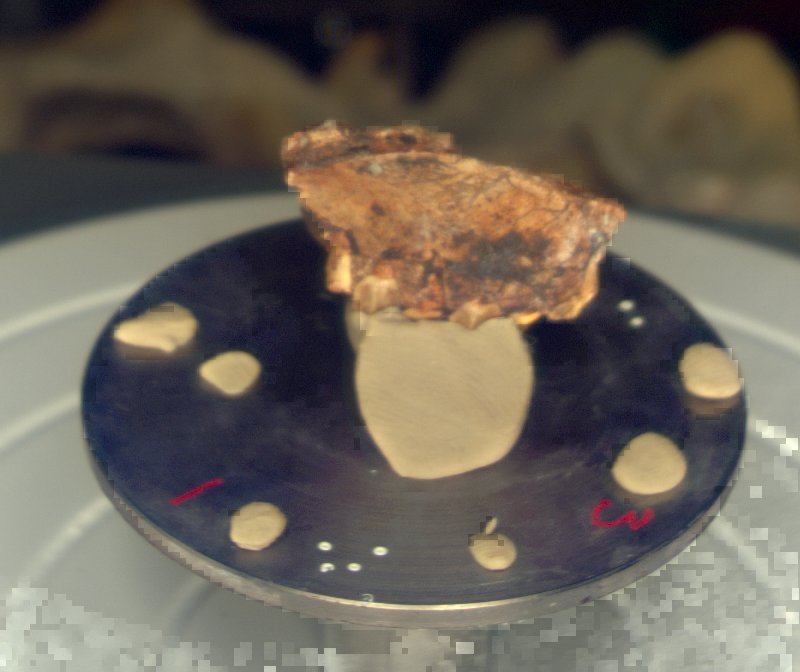

Supplement: File S7 — Surface scans of elements from the lower jaw of Aquilops americanus , OMNH 34557 (holotype). (ZIP) [file pone.0112055.s007.zip › Posterior Dentary/MainDentary_T5.jpg]

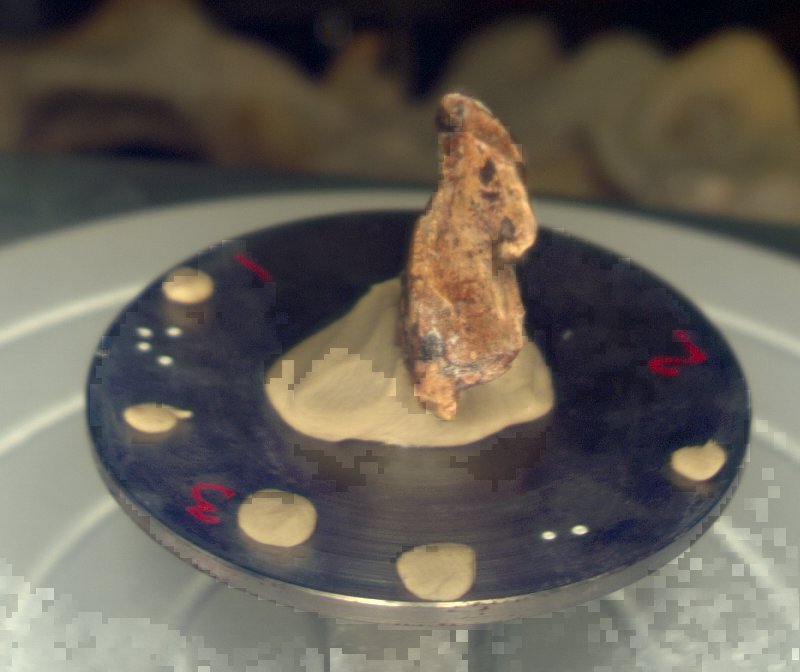

Supplement: File S7 — Surface scans of elements from the lower jaw of Aquilops americanus , OMNH 34557 (holotype). (ZIP) [file pone.0112055.s007.zip › Posterior Dentary/MainDentary_T7.jpg]

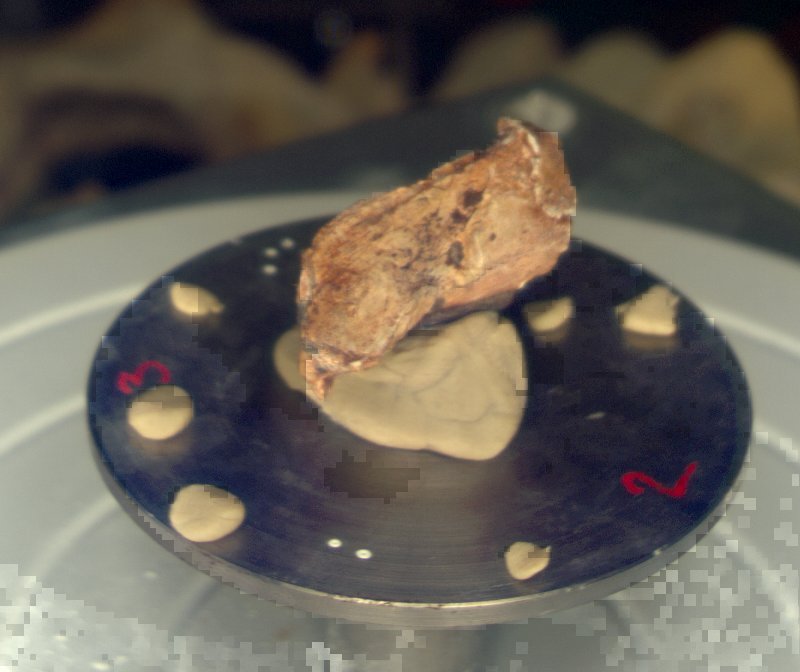

Supplement: File S7 — Surface scans of elements from the lower jaw of Aquilops americanus , OMNH 34557 (holotype). (ZIP) [file pone.0112055.s007.zip › Posterior Dentary/MainDentary_T8.jpg]

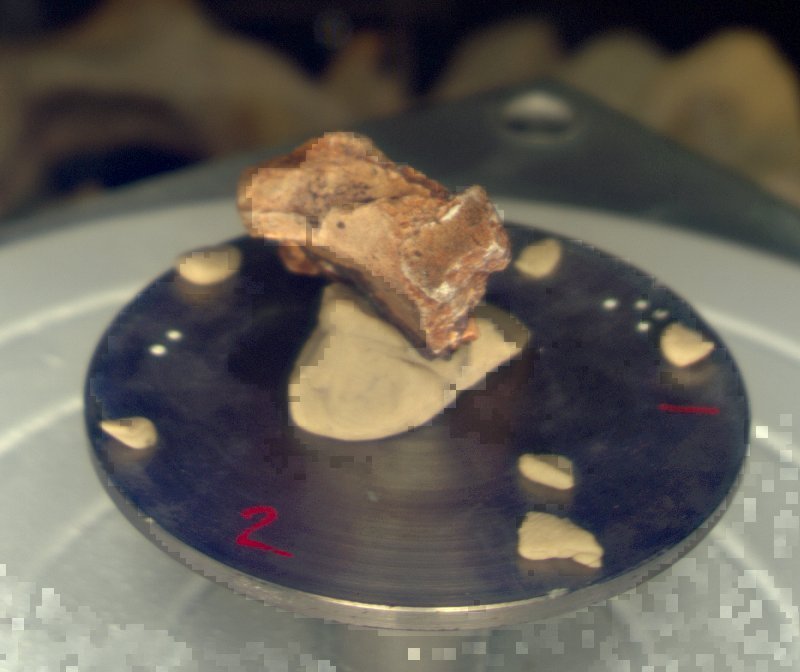

Supplement: File S7 — Surface scans of elements from the lower jaw of Aquilops americanus , OMNH 34557 (holotype). (ZIP) [file pone.0112055.s007.zip › Posterior Dentary/MainDentary_T2.jpg]

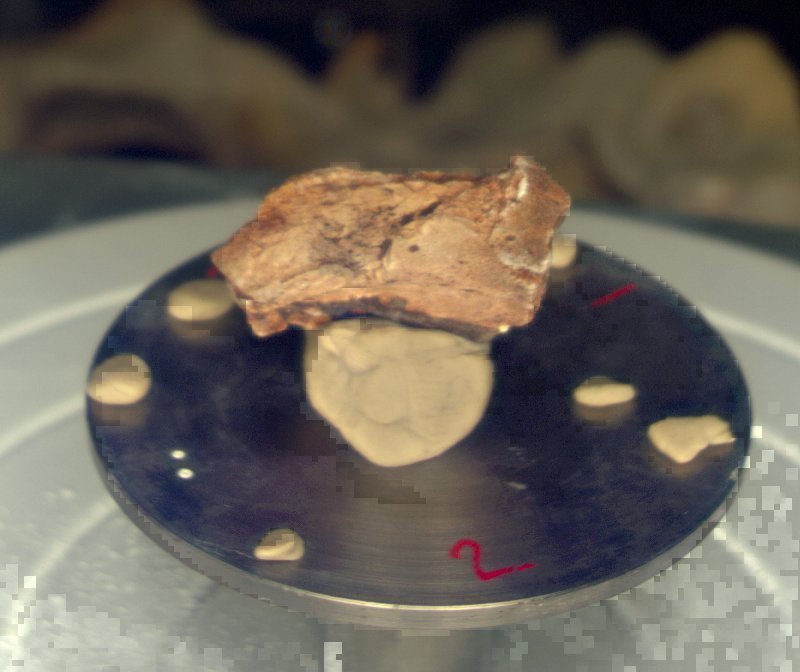

Supplement: File S7 — Surface scans of elements from the lower jaw of Aquilops americanus , OMNH 34557 (holotype). (ZIP) [file pone.0112055.s007.zip › Posterior Dentary/MainDentary_T1.jpg]

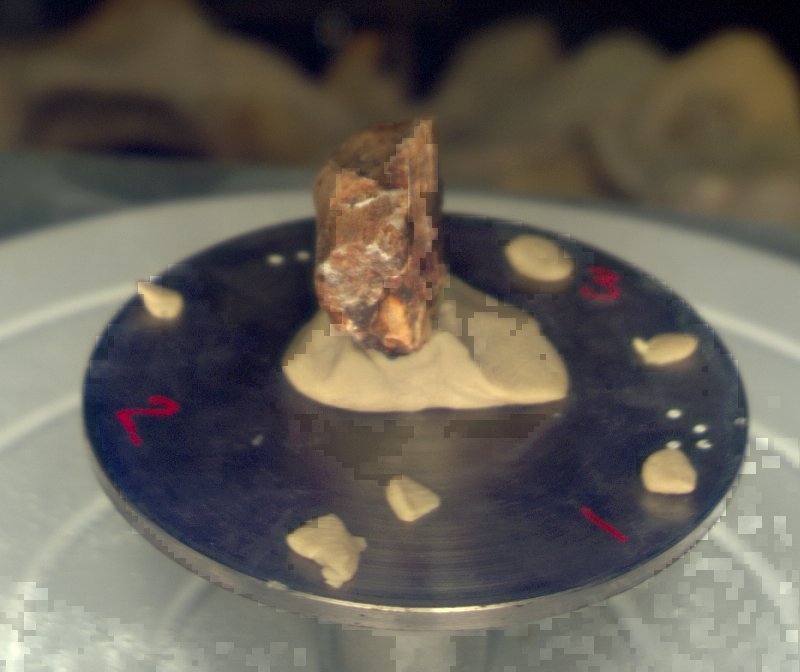

Supplement: File S7 — Surface scans of elements from the lower jaw of Aquilops americanus , OMNH 34557 (holotype). (ZIP) [file pone.0112055.s007.zip › Posterior Dentary/MainDentary_T3.jpg]

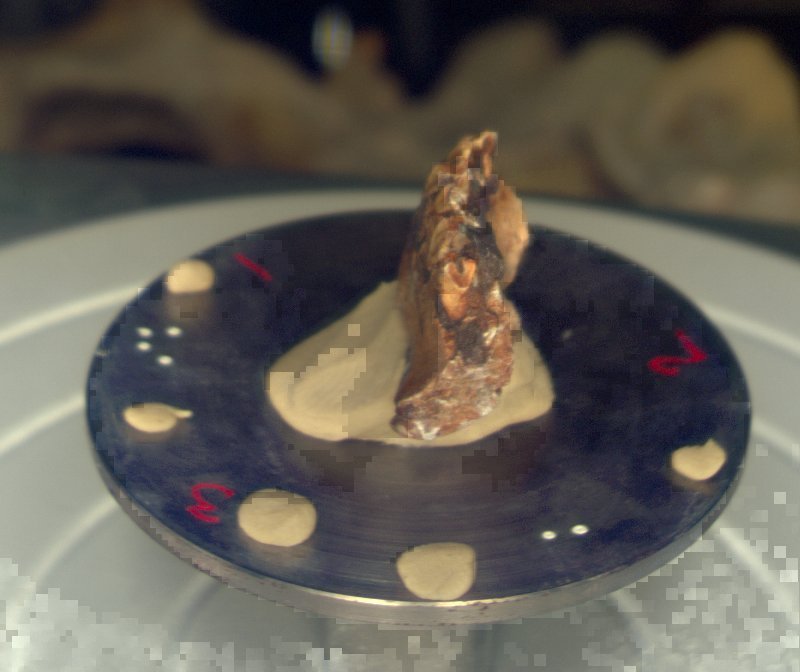

Supplement: File S7 — Surface scans of elements from the lower jaw of Aquilops americanus , OMNH 34557 (holotype). (ZIP) [file pone.0112055.s007.zip › Posterior Dentary/MainDentary_T15.jpg]

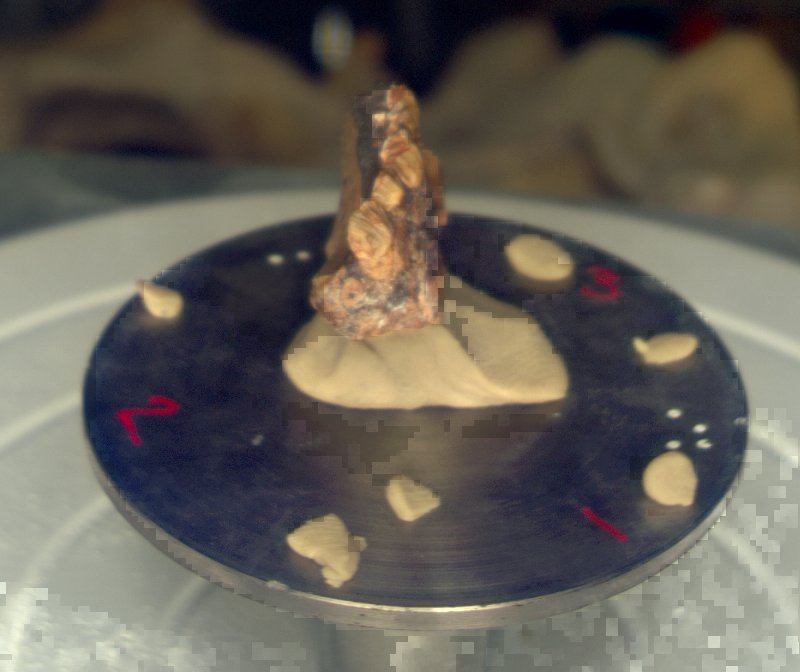

Supplement: File S7 — Surface scans of elements from the lower jaw of Aquilops americanus , OMNH 34557 (holotype). (ZIP) [file pone.0112055.s007.zip › Posterior Dentary/MainDentary_T11.jpg]

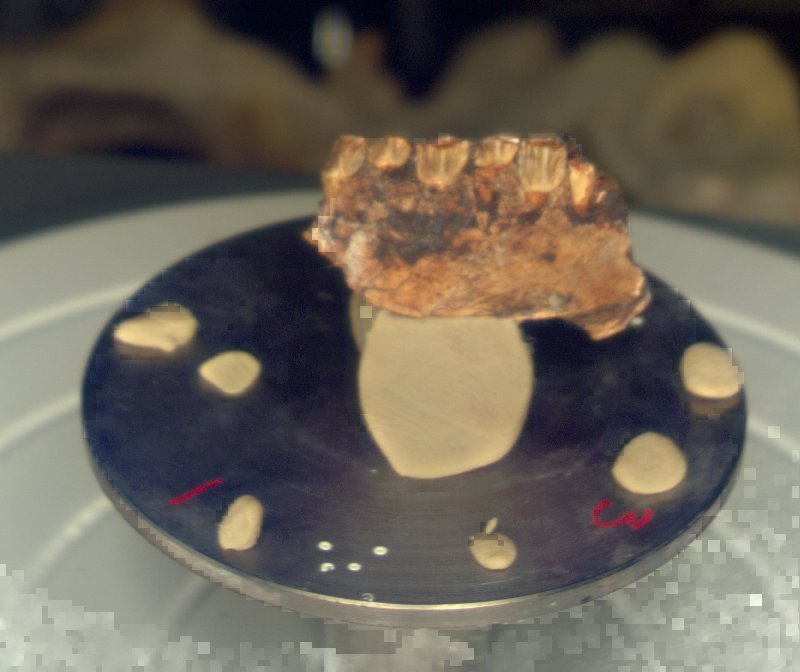

Supplement: File S7 — Surface scans of elements from the lower jaw of Aquilops americanus , OMNH 34557 (holotype). (ZIP) [file pone.0112055.s007.zip › Posterior Dentary/MainDentary_T13.jpg]

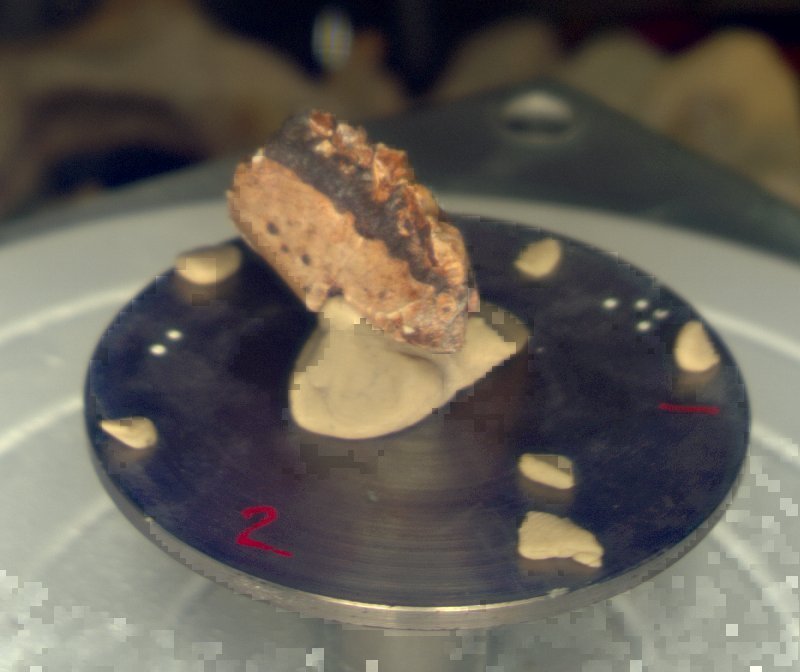

Supplement: File S7 — Surface scans of elements from the lower jaw of Aquilops americanus , OMNH 34557 (holotype). (ZIP) [file pone.0112055.s007.zip › Posterior Dentary/MainDentary_T10.jpg]

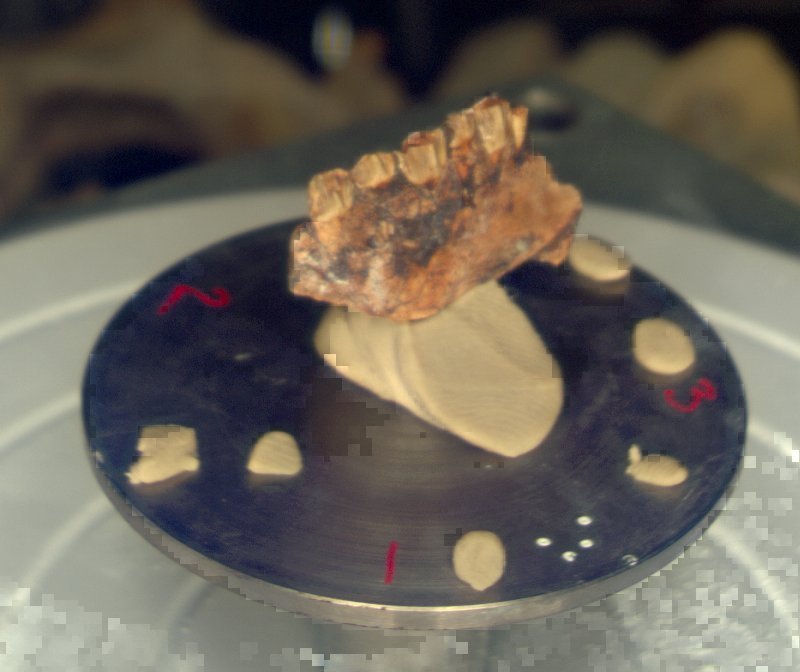

Supplement: File S7 — Surface scans of elements from the lower jaw of Aquilops americanus , OMNH 34557 (holotype). (ZIP) [file pone.0112055.s007.zip › Posterior Dentary/MainDentary_T12.jpg]

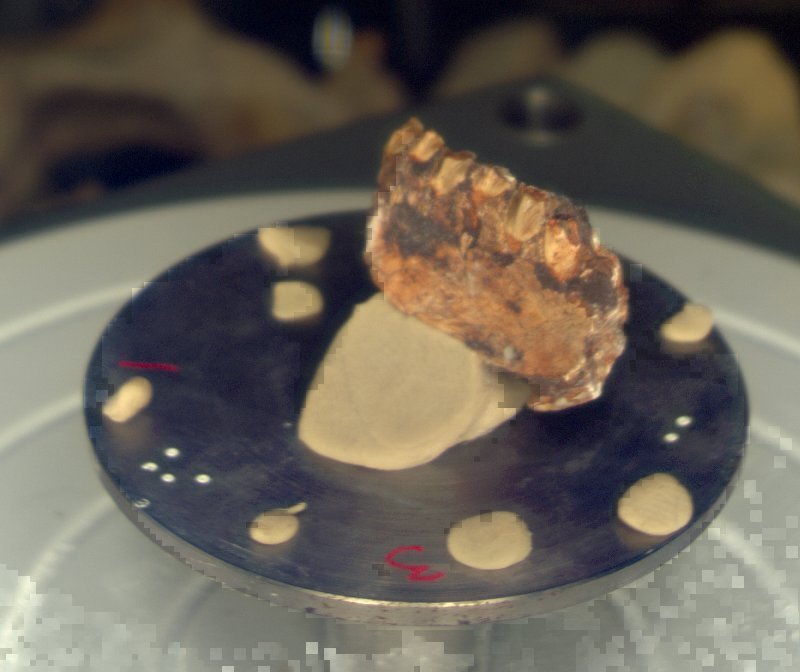

Supplement: File S7 — Surface scans of elements from the lower jaw of Aquilops americanus , OMNH 34557 (holotype). (ZIP) [file pone.0112055.s007.zip › Posterior Dentary/MainDentary_T14.jpg]

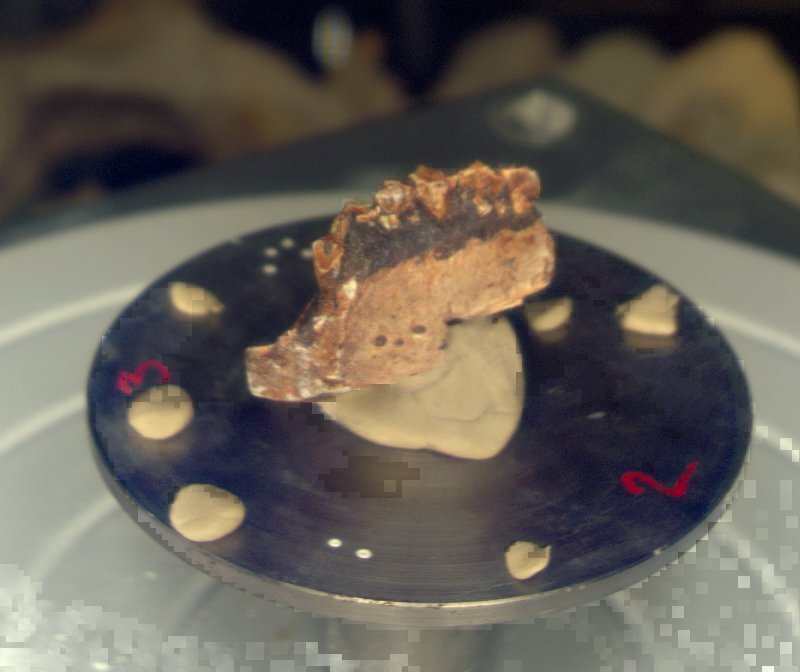

Supplement: File S7 — Surface scans of elements from the lower jaw of Aquilops americanus , OMNH 34557 (holotype). (ZIP) [file pone.0112055.s007.zip › Posterior Dentary/MainDentary_T16.jpg]

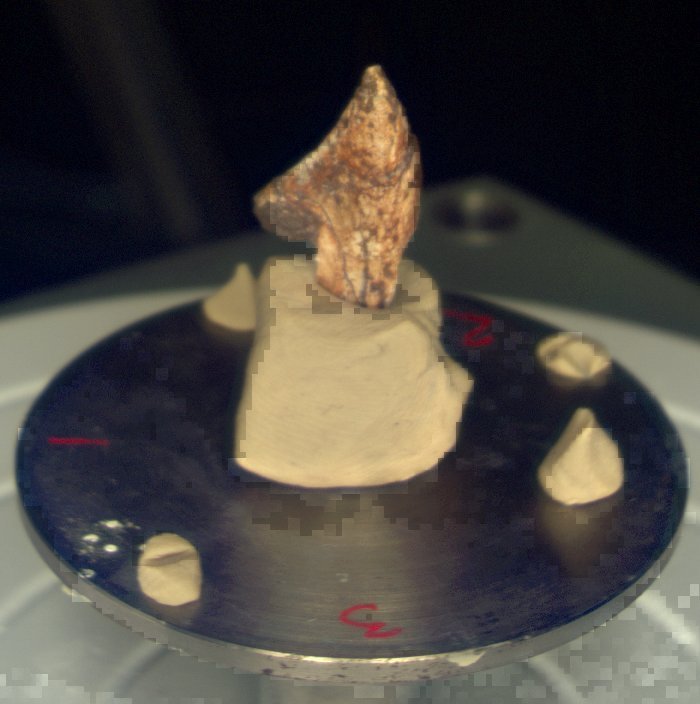

Supplement: File S7 — Surface scans of elements from the lower jaw of Aquilops americanus , OMNH 34557 (holotype). (ZIP) [file pone.0112055.s007.zip › Predentary/Predentary_T13.jpg]

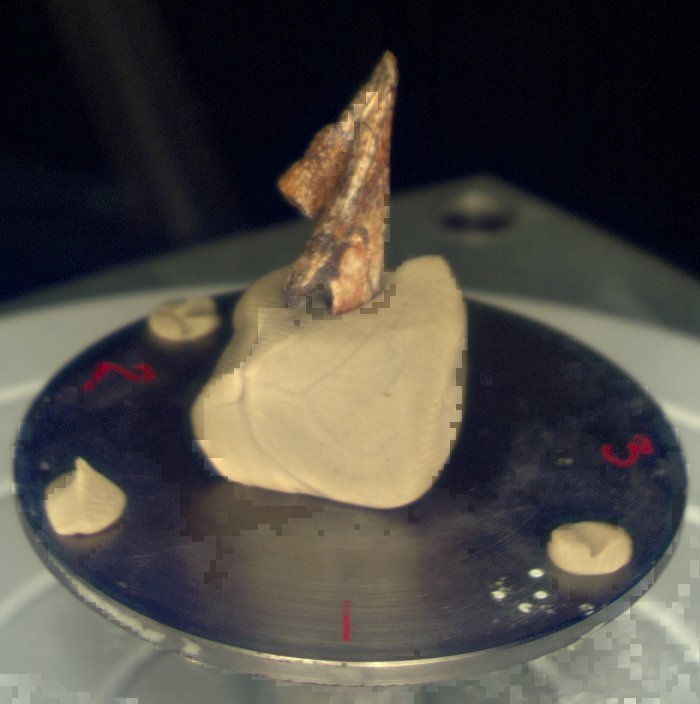

Supplement: File S7 — Surface scans of elements from the lower jaw of Aquilops americanus , OMNH 34557 (holotype). (ZIP) [file pone.0112055.s007.zip › Predentary/Predentary_T11.jpg]

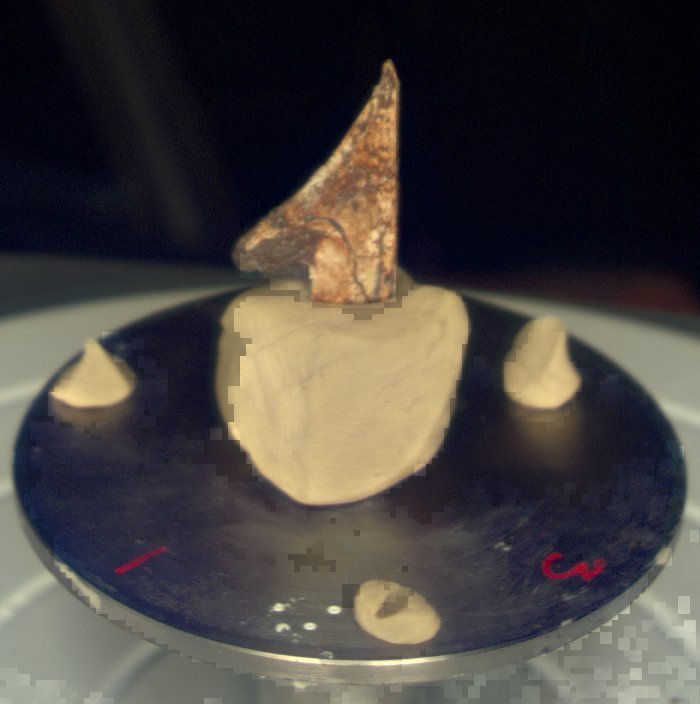

Supplement: File S7 — Surface scans of elements from the lower jaw of Aquilops americanus , OMNH 34557 (holotype). (ZIP) [file pone.0112055.s007.zip › Predentary/Predentary_T12.jpg]

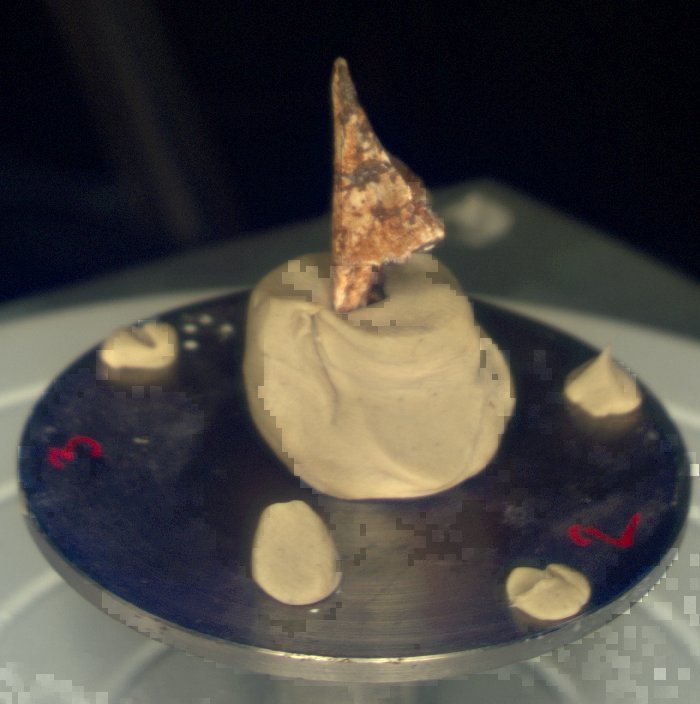

Supplement: File S7 — Surface scans of elements from the lower jaw of Aquilops americanus , OMNH 34557 (holotype). (ZIP) [file pone.0112055.s007.zip › Predentary/Predentary_T15.jpg]

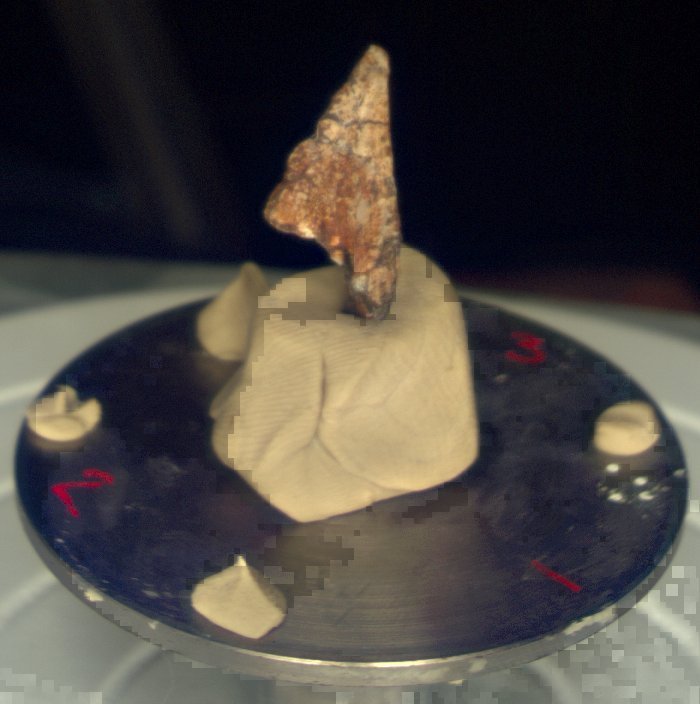

Supplement: File S7 — Surface scans of elements from the lower jaw of Aquilops americanus , OMNH 34557 (holotype). (ZIP) [file pone.0112055.s007.zip › Predentary/Predentary_T10.jpg]

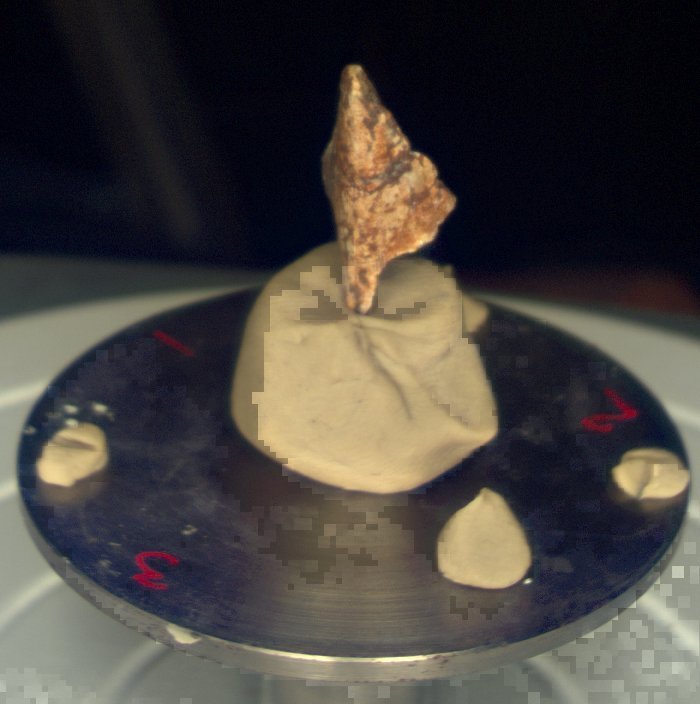

Supplement: File S7 — Surface scans of elements from the lower jaw of Aquilops americanus , OMNH 34557 (holotype). (ZIP) [file pone.0112055.s007.zip › Predentary/Predentary_T14.jpg]

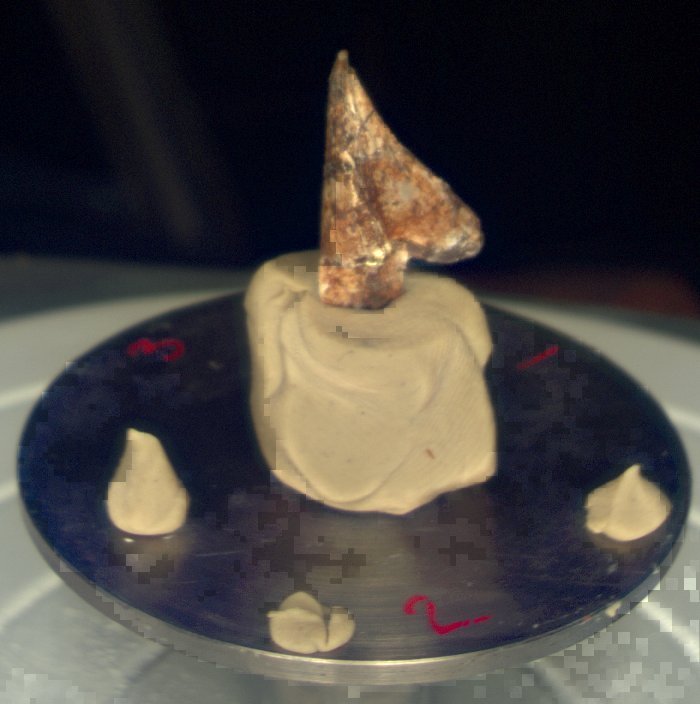

Supplement: File S7 — Surface scans of elements from the lower jaw of Aquilops americanus , OMNH 34557 (holotype). (ZIP) [file pone.0112055.s007.zip › Predentary/Predentary_T8.jpg]

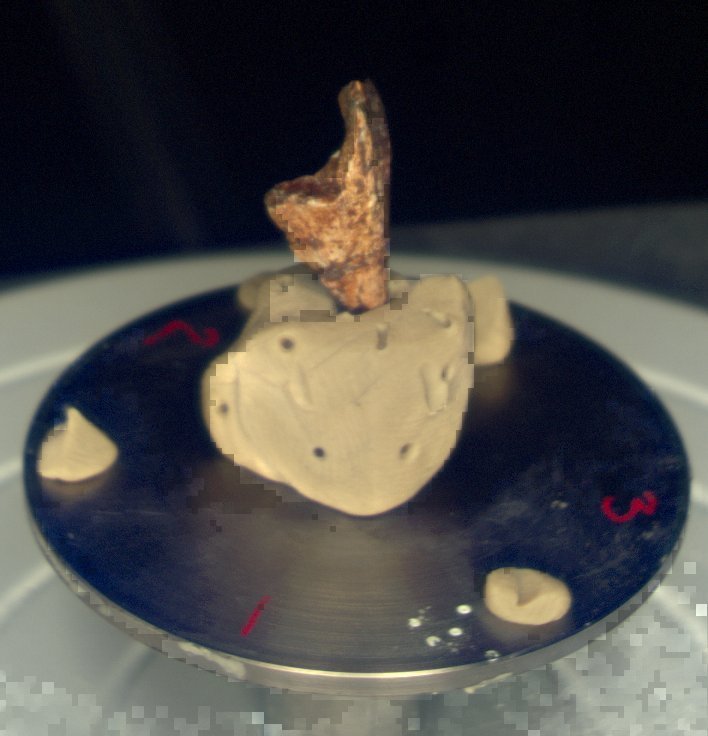

Supplement: File S7 — Surface scans of elements from the lower jaw of Aquilops americanus , OMNH 34557 (holotype). (ZIP) [file pone.0112055.s007.zip › Predentary/Predentary_T4.jpg]

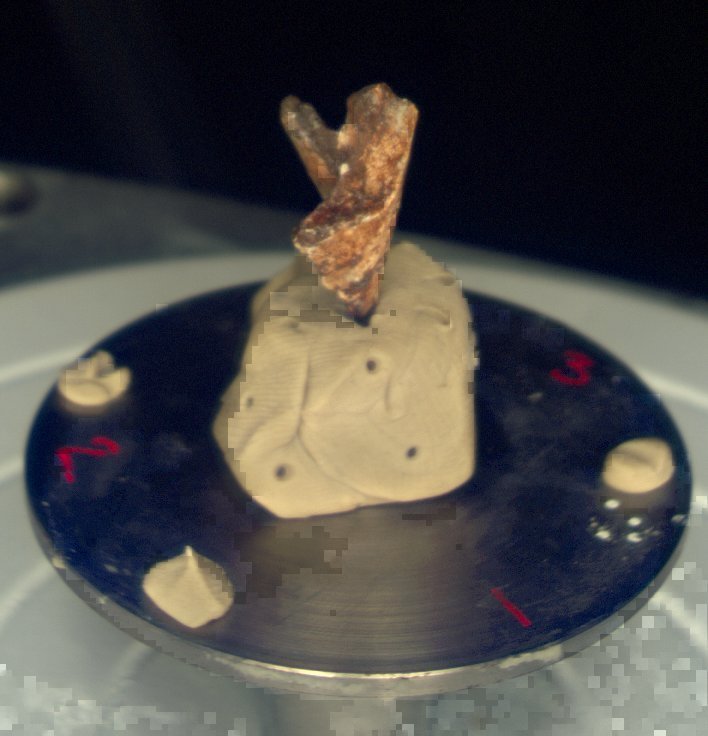

Supplement: File S7 — Surface scans of elements from the lower jaw of Aquilops americanus , OMNH 34557 (holotype). (ZIP) [file pone.0112055.s007.zip › Predentary/Predentary_T3.jpg]

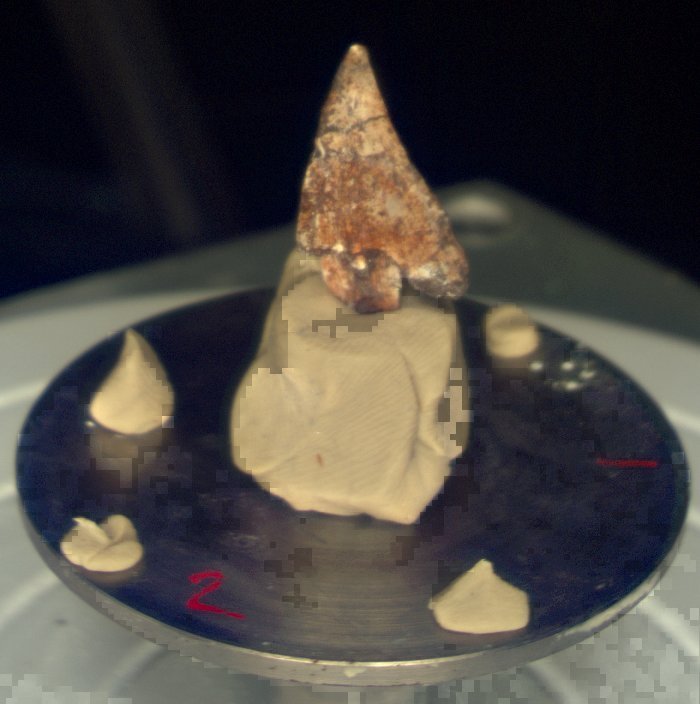

Supplement: File S7 — Surface scans of elements from the lower jaw of Aquilops americanus , OMNH 34557 (holotype). (ZIP) [file pone.0112055.s007.zip › Predentary/Predentary_T9.jpg]

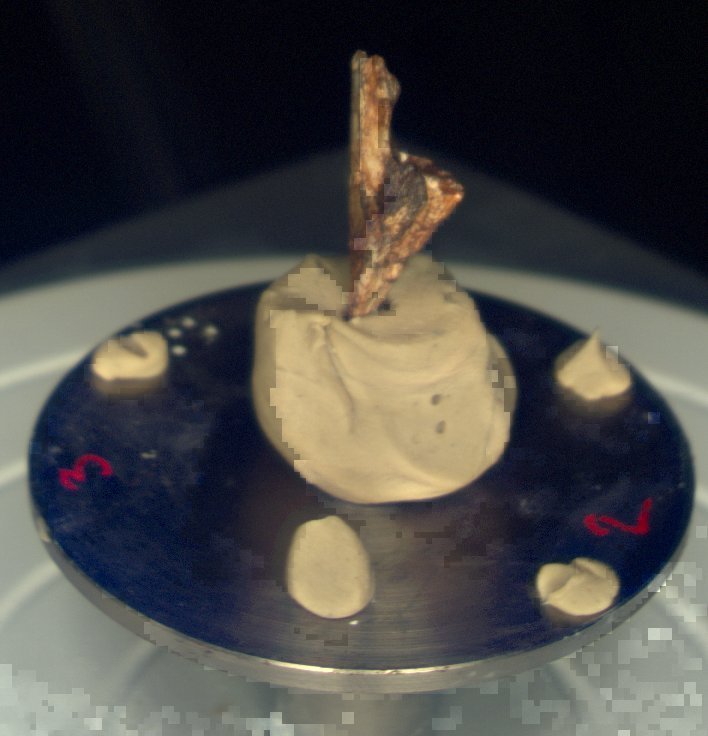

Supplement: File S7 — Surface scans of elements from the lower jaw of Aquilops americanus , OMNH 34557 (holotype). (ZIP) [file pone.0112055.s007.zip › Predentary/Predentary_T7.jpg]

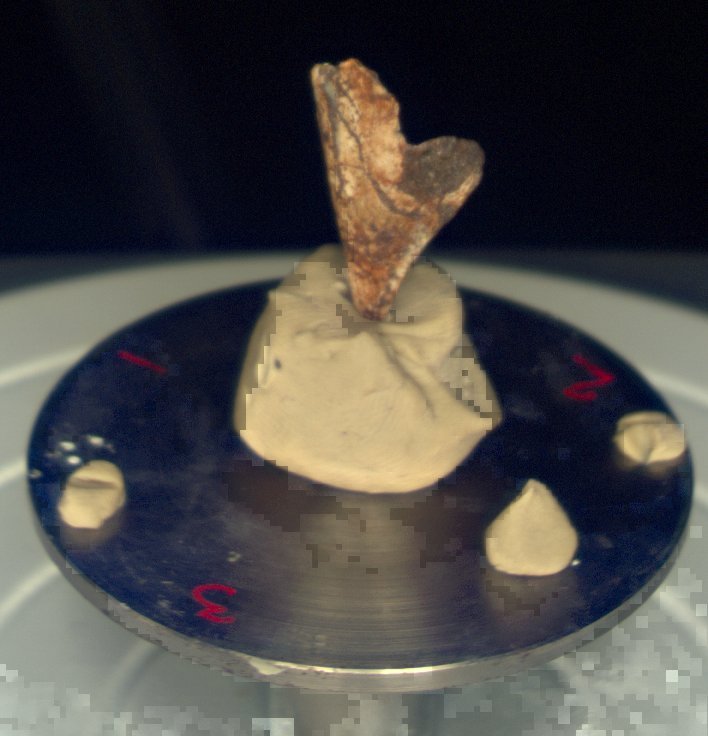

Supplement: File S7 — Surface scans of elements from the lower jaw of Aquilops americanus , OMNH 34557 (holotype). (ZIP) [file pone.0112055.s007.zip › Predentary/Predentary_T6.jpg]

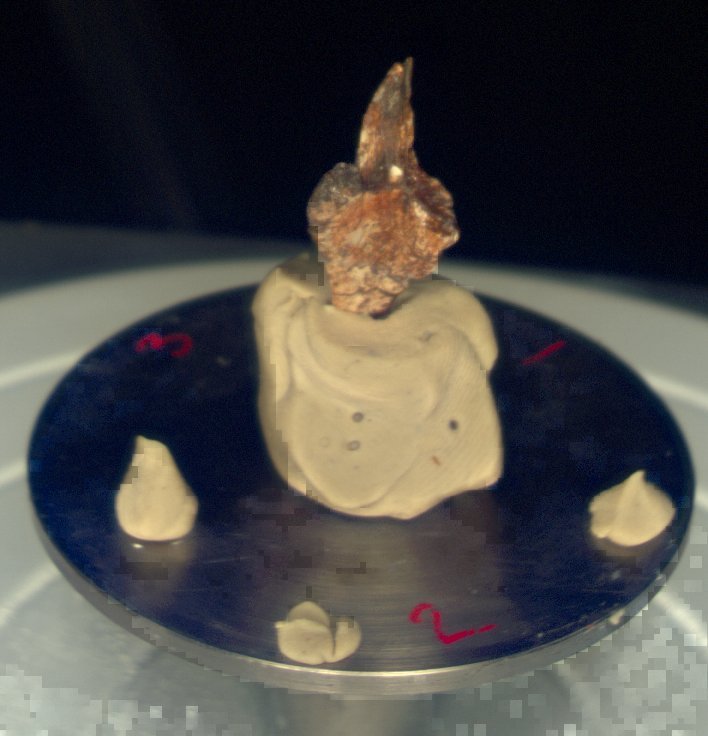

Supplement: File S7 — Surface scans of elements from the lower jaw of Aquilops americanus , OMNH 34557 (holotype). (ZIP) [file pone.0112055.s007.zip › Predentary/Predentary_T1.jpg]

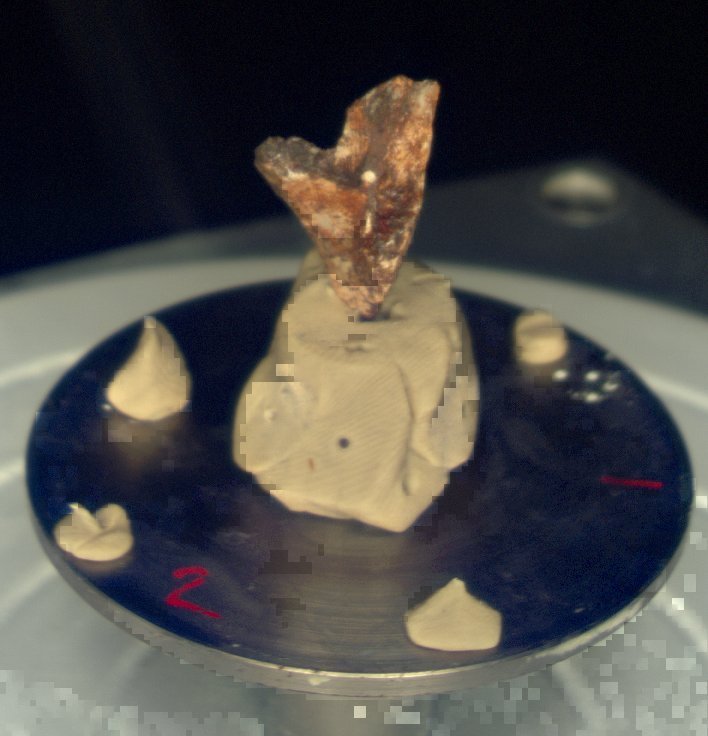

Supplement: File S7 — Surface scans of elements from the lower jaw of Aquilops americanus , OMNH 34557 (holotype). (ZIP) [file pone.0112055.s007.zip › Predentary/Predentary_T2.jpg]

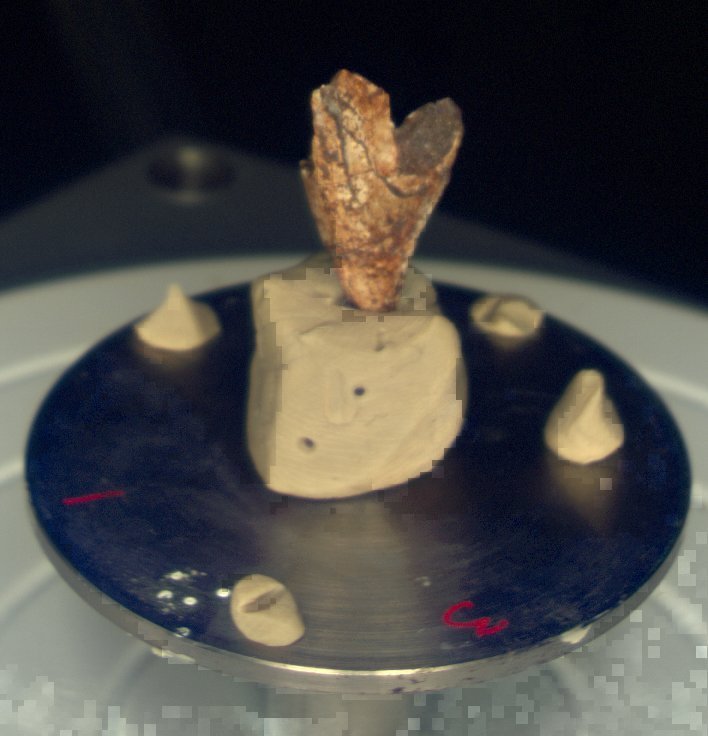

Supplement: File S7 — Surface scans of elements from the lower jaw of Aquilops americanus , OMNH 34557 (holotype). (ZIP) [file pone.0112055.s007.zip › Predentary/Predentary_T5.jpg]

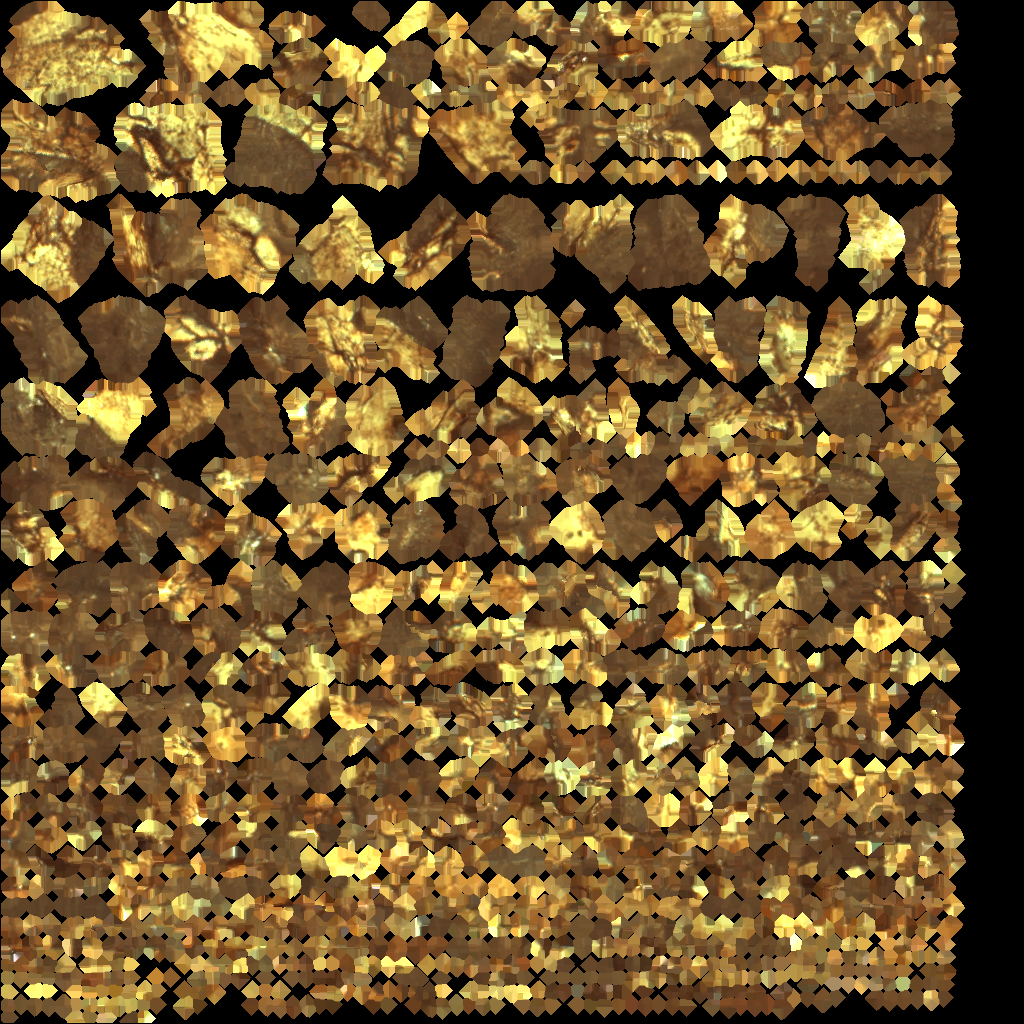

Supplement: File S9 — Color surface scan of the cranium of Aquilops americanus , OMNH 34557 (holotype). (ZIP) [file pone.0112055.s009.zip › 85_final_(4).bmp]

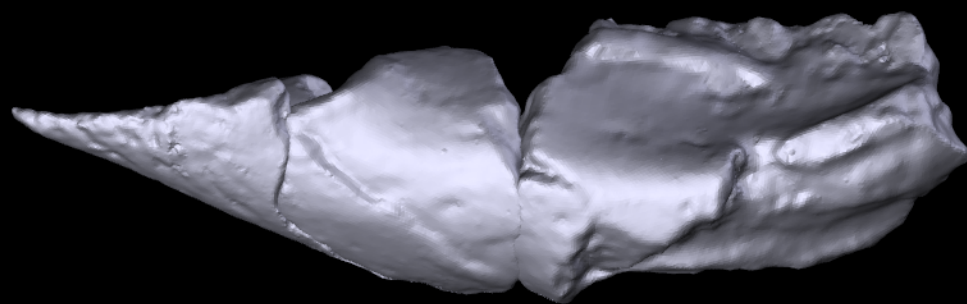

Supplement: File S10 — 3D PDF of the lower jaw of Aquilops americanus , OMNH 34557 (holotype), based on scans from File S7. (PDF) [file pone.0112055.s010.pdf]

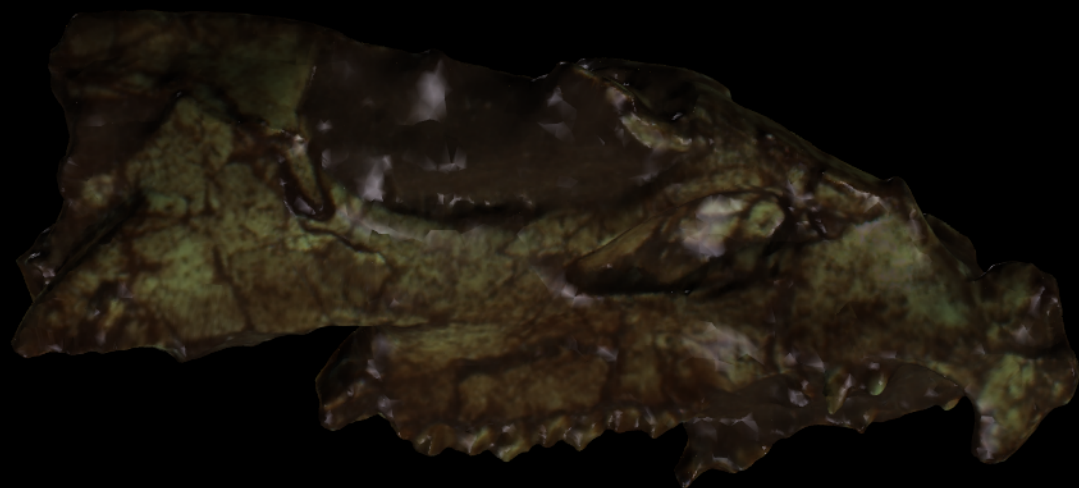

Supplement: File S11 — Color 3D PDF of the cranium of Aquilops americanus , OMNH 34557 (holotype), based on scans from File S9. (PDF) [file pone.0112055.s011.pdf]

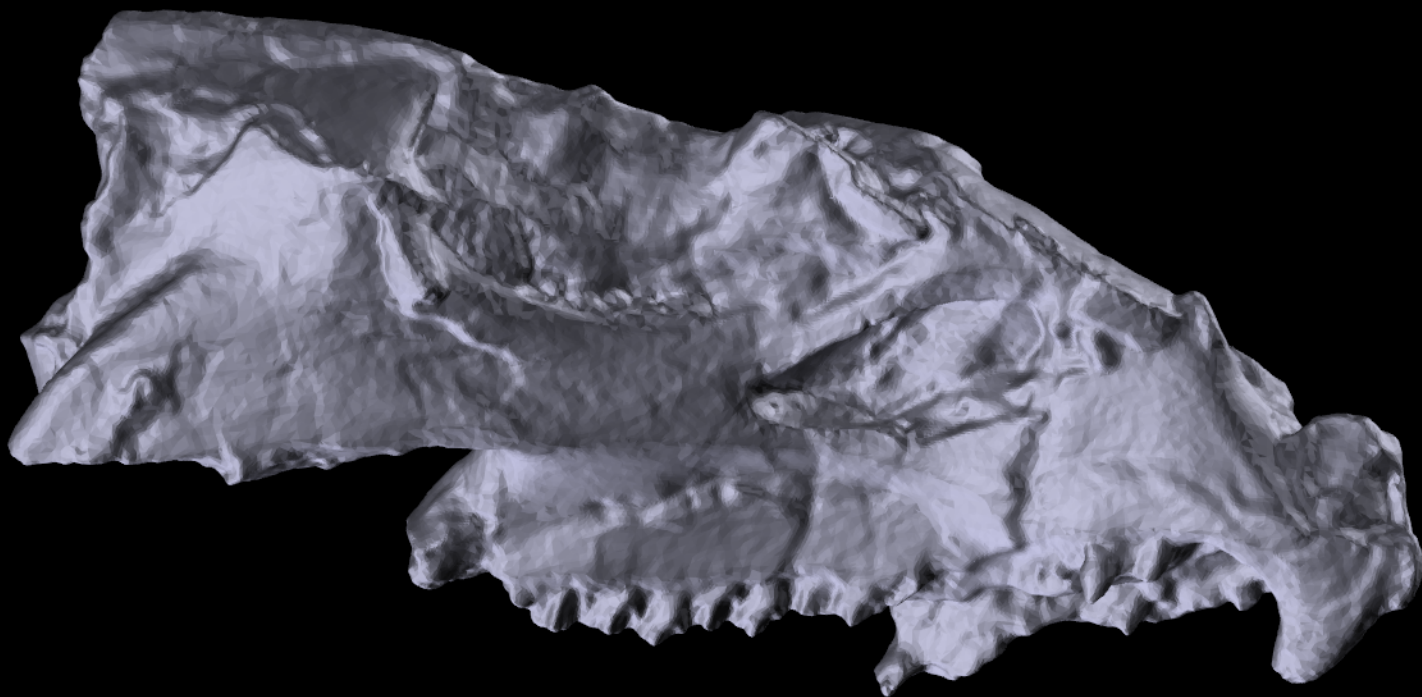

Supplement: File S12 — 3D PDF of the cranium of Aquilops americanus , OMNH 34557 (holotype), based on scans from File S8. (PDF) [file pone.0112055.s012.pdf]
